# LRBA deficiency impairs autophagy and contributes to enhanced antigen presentation and T-cell dysregulation

**DOI:** 10.1038/s44319-025-00504-7

**Published:** 2025-06-23

**Authors:** Elena Sindram, Marie-Celine Deau, Laure-Anne Ligeon, Pablo Sanchez-Martin, Sigrun Nestel, Sophie Jung, Stefanie Ruf, Pankaj Mishra, Michele Proietti, Stefan Günther, Kathrin Thedieck, Eleni Roussa, Angelika Rambold, Christian Münz, Claudine Kraft, Bodo Grimbacher, Laura Gámez-Díaz

**Affiliations:** 1 Institute for Immunodeficiency, Center for Chronic Immunodeficiency (CCI), Medical Center—University of Freiburg, Faculty of Medicine, University of Freiburg, Freiburg, Germany; 2 Spemann Graduate School of Biology and Medicine (SGBM), University of Freiburg, Freiburg, Germany; 3 Faculty of Biology, University of Freiburg, Freiburg, Germany; 4 Viral Immunobiology, Institute of Experimental Immunology, University of Zürich, Zürich, Switzerland; 5 Institute of Biochemistry and Molecular Biology, ZBMZ, Faculty of Medicine, University of Freiburg, Freiburg, Germany; 6 Institute of Anatomy and Cell Biology Department of Neuroanatomy, Faculty of Medicine, University of Freiburg, Freiburg, Germany; 7 Faculté de Chirurgie Dentaire, Université de Strasbourg—Pôle de Médecine et de Chirurgie Bucco-Dentaires, Hôpitaux Universitaires de Strasbourg, Strasbourg, France; 8 Laboratory of Pediatrics, Section Systems Medicine of Metabolism and Signaling, University of Groningen, University Medical Center Groningen, Groningen, The Netherlands; 9 Research group of Pharmaceutical Bioinformatics, Institute of Pharmaceutical Sciences, University of Freiburg, Freiburg, Germany; 10 Department of Rheumatology and Immunology, Hannover Medical School, Hannover, Germany; 11 RESIST—Cluster of Excellence 2155, Hanover Medical School, Satellite Center Freiburg, Freiburg, Germany; 12 Department for Neuroscience, School of Medicine and Health Sciences, Carl von Ossietzky University Oldenburg, Oldenburg, Germany; 13 Institute of Biochemistry and Center for Molecular Biosciences Innsbruck, University of Innsbruck, Innsbruck, Austria; 14 Institute of Anatomy and Cell Biology, Department of Molecular Embryology, Faculty of Medicine, University of Freiburg, Freiburg, Germany; 15 Laboratory for Structural Metabolism of Inflammation, Institute for Medical Biochemistry, Center for Molecular Biology of Inflammation, University of Münster, Münster, Germany; 16 CIBSS—Centre for Integrative Biological Signalling Studies, University of Freiburg, Freiburg, Germany; 17 Clinic of Rheumatology and Clinical Immunology, Center for Chronic Immunodeficiency (CCI), Medical Center—University of Freiburg, Faculty of Medicine, University of Freiburg, Freiburg, Germany; 18 DZFI—German Center for Infection Research, Satellite Center Freiburg, Freiburg, Germany

**Keywords:** Autophagy, FYCO1, Immune Dysregulation, LRBA, PIK3R4, Autophagy & Cell Death, Immunology, Molecular Biology of Disease

## Abstract

Reduced autophagy is associated with the aberrant humoral response observed in lipopolysaccharide-responsive beige-like anchor protein (LRBA) deficiency; however, the molecular mechanisms and their impact on T-cell responses remain poorly understood. We identify two novel LRBA interactors, phosphoinositide 3-kinase regulatory subunit 4 (PIK3R4) and FYVE And Coiled-Coil Domain Autophagy Adaptor 1 (FYCO1), which each play key roles in autophagy. PIK3R4 facilitates the production of phosphatidylinositol-3 phosphate (PI(3)P) that promotes autophagosome formation and autophagosome-lysosome fusion, whereas FYCO1 supports autophagosome movement. LRBA-knockout (KO) cells show impaired PI(3)P production, reduced autophagosome-lysosome fusion, accumulation of enlarged autophagosomes, and decreased cargo degradation. In line with the role of autophagy as a major degradation system for MHC-II loading and antigen presentation, we observe increased numbers of MHC class II and LC3 vesicles, along with enhanced antigen presentation in absence of LRBA, resulting in a higher production of proinflammatory cytokines from T cells in vitro. Our work suggests a novel biological role of LRBA controlling antigen presentation and T-cell responses by positively regulating autophagy, which may contribute to T-cell immune dysregulation observed in LRBA-deficient patients.

## Synopsis

LRBA regulates autophagy and antigen presentation, shedding light on the mechanism of immune dysregulation in LRBA deficiency.

- LRBA directly interacts with PIK3R4 and FYCO1, promoting autophagosome formation, transport, and fusion with lysosomes.
- LRBA knockout impairs PI(3)P production and autophagosome-lysosome fusion, leading to accumulation of enlarged autophagosomes with reduced cargo degradation.
- Defective cargo degradation in LRBA-deficient cells increases antigenic peptides in MIIC vesicles, enhancing antigen presentation and T-cell driven proinflammatory cytokine production.

## Abstract (web summary)

LRBA regulates autophagy and antigen presentation, shedding light on the mechanism of immune dysregulation in LRBA deficiency.

## Graphical abstract

**Figure:**
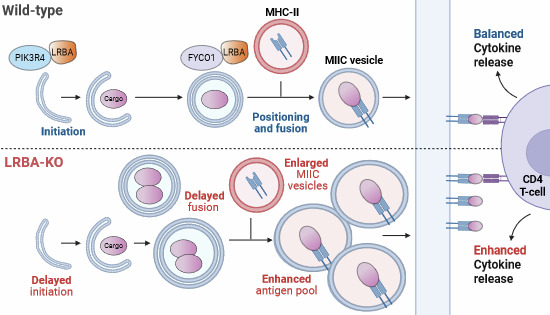

**Figure:**
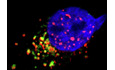

## Introduction

LRBA deficiency is a rare genetic immune disorder caused by deleterious biallelic mutations in *LRBA* (Lopez-Herrera et al, 2012). Clinically, it manifests with a broad spectrum of symptoms ranging from immunodeficiency to T-cell driven immune dysregulation, including inflammatory bowel disease (IBD) and autoimmune cytopenias (Alkhairy et al, 2016; Azizi et al, 2017a; Gamez-Diaz et al, 2016; Kostel Bal et al, 2017). Mechanistically, LRBA regulates the vesicular recycling of the cytotoxic T lymphocyte-associated protein 4 (CTLA-4) on regulatory T cells (Tregs), partly explaining the T-cell immune dysregulation upon loss of LRBA (Janman et al, 2021; Lo et al, 2015). Although the clinical pictures of LRBA deficiency and CTLA-4 insufficiency frequently overlap (Jamee et al, 2021; Lo et al, 2016), LRBA-deficient patients display an increased severity, poorer survival and earlier onset of symptoms than CTLA-4 insufficiency patients. These observations suggest that LRBA loss triggers additional disease mechanisms, beyond abnormal CTLA-4 trafficking.

We previously reported that B lymphocytes from LRBA-deficient patients exhibit defective autophagy leading to poor B cell survival (Lopez-Herrera et al, 2012). This defect pointed towards impaired fusion between autophagosomes and lysosomes and/or non-functional lysosomes; however, the mechanism remained unclear. Autophagy occurs in all eukaryotic cells, engulfing cytoplasmic material, including aberrant proteins or intracellular pathogens, in vesicles called autophagosomes, which subsequently fuse with lysosomes for cargo degradation (Mizushima, 2007). Beyond maintaining cellular homeostasis, autophagy regulates immune recognition and responsiveness. For instance, autophagy supports antigen processing and MHC-II-mediated antigen presentation in professional and non-professional antigen presenting cells (Cui et al, 2019; Dikic and Elazar, 2018; Schmid et al, 2007). Specifically, autophagosomes fuse with multivesicular MHC-II-loading compartments (MIIC), leading to the degradation of intracellular proteins and pathogens into peptides that can be loaded onto MHC-II molecules (Schmid et al, 2007). Whether aberrant autophagy arising from LRBA loss impacts antigen presentation is unknown. Moreover, IBD, a prominent symptom of LRBA deficiency characterized by chronic inflammation, is also linked to abnormal autophagy (Hampe et al, 2007; Shao et al, 2021).

In this study, we demonstrate that LRBA physically interacts with proteins that are essential for autophagy, including phosphoinositide-3-kinase regulatory subunit 4 (PIK3R4, also known as VPS15) and UV radiation resistance associated (UVRAG), two components of a phosphatidylinositol 3-kinase III (PI3K-III) complex. We show that LRBA loss diminished PI(3)P production, altered subcellular localization of PI(3)P-binding proteins, inhibited autophagosome-lysosome fusion, and led to autophagosome accumulation and abnormal autophagy flux in multiple cell types. In addition, we show that LRBA interacts with FYVE and Coiled-Coil Domain Autophagy Adaptor 1 (FYCO1) (Nieto-Torres et al, 2021), which links autophagosomes to microtubule plus-end-directed molecular motors, and regulates autophagosome movement (Pankiv et al, 2010; Pankiv and Johansen, 2010). Importantly, we provide evidence that LRBA regulates MHC-II-restricted antigen presentation via autophagy, thereby regulating stimulation of CD4+ T cells and curbing the release of T-cell proinflammatory cytokines. This novel function of LRBA in regulating antigen presentation may contribute to the T-cell immune dysregulation observed in LRBA-deficient patients.

## Results

### LRBA interacts with components of a phosphatidylinositol 3-kinase III (PI3K-III) complex

**Figure 1:**
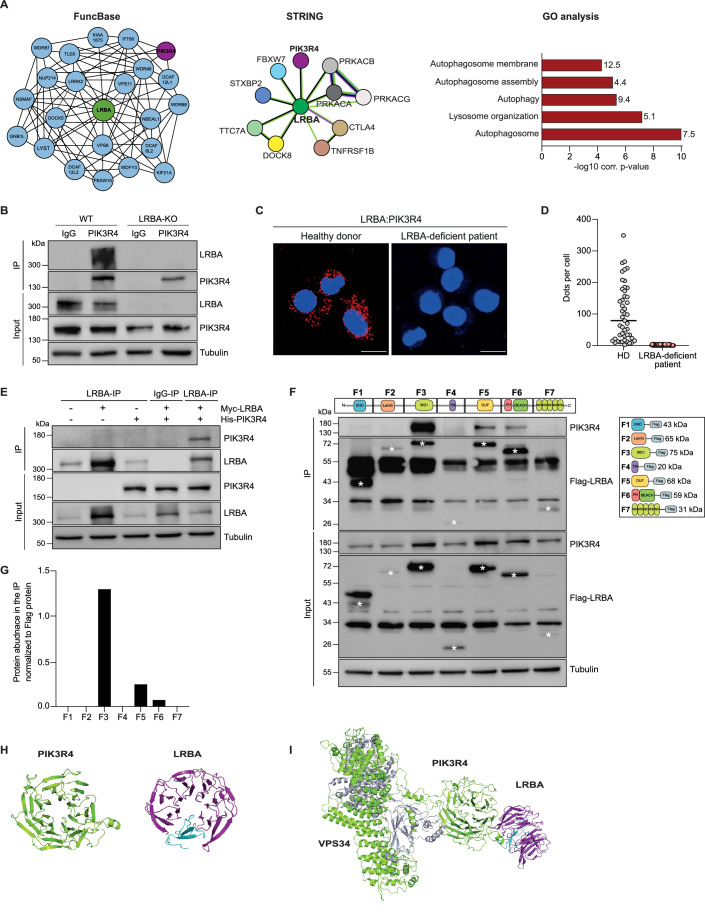
LRBA interacts with PIK3R4 through its WD40 domain. (**A**) In silico identification of LRBA protein interaction network using FuncBase (left) and STRING version 12 (middle). In STRING, colored lines indicate evidence of text mining (green), co-occurrence (blue), co-expression (black) and experimental evidence (purple). GO enrichment analyses (right) of 31 potential interactors of LRBA for the GO domain “cellular compartment”. The length of the bar represents the log10 Benjamini–Hochberg corrected *P* value. The numbers represent the percentage of associated genes for each biological process. (**B**) Co-IP analyses of endogenous LRBA and PIK3R4 interaction in WT and LRBA-KO HEK293T cells. PIK3R4 was immunoprecipitated with anti-PIK3R4. Western blot analysis of the immunoprecipitation and the input was performed with the indicated antibodies. Tubulin expression was used as an input control. (**C**) Representative fluorescent microscopy images of PLA signal in healthy donor (HD) and LRBA-deficient patient LCL cells. LRBA-PIK3R4 interaction is shown as a red signal and nuclear DAPI staining is shown in blue. Scale bar = 20 µm. (**D**) Dots (PLA signal) per cell were quantified in 50 cells per experiment from *n* = 2 biological replicates of HD (grey) and LRBA-deficient (red) LCL cells using Duolink Image tool. (**E**) HEK293T WT cells were co-transfected with full-length Myc-LRBA plasmid and/or full-length Myc-VPS34/His-PIK3R4 plasmid (VPS34 blots are shown in Fig. EV1C). Immunoprecipitation was performed with anti-LRBA or anti-IgG and immunoblotted for PIK3R4. (**F**) HEK293T WT cells were co-transfected with His-tagged full-length PIK3R4 and Flag-tagged LRBA protein domains (fragments 1 to 7). Flag-LRBA was immunoprecipitated with anti-Flag, and western blots were probed with anti-PIK3R4. Schematic representation of plasmids containing the different LRBA protein domains is shown on the top and right. Asterisks in the western blots indicate the LRBA protein domains. (**G**) Ratio of PIK3R4/LRBA fragment detected by co-IP was calculated for *n* = 1 biological replicates. (**H**) Models of the WD40 domains of PIK3R4 (light green) and LRBA (purple) using the model server WDSPdb 2.0. The inserted repeat propeller of LRBA is shown in cyan. (**I**) Model of the complex of LRBA (purple) with PIK3R4 (light green)/PIK3C3 (VPS34, silver). The inserted repeat propeller of LRBA-WD40 is shown in cyan. Source data are available online for this figure.

Using computational predictions based on STRING (v12) and FuncBase databases (Beaver et al, 2010; Szklarczyk et al, 2017), we identified 31 potential novel LRBA interactors that were enriched in autophagy and vesicle-trafficking events according to Gene Ontology (GO) term enrichment analyses (Fig. 1A; Table 1). PIK3R4, a component of the PI3K-III core complex that controls autophagy and endocytosis, was predicted to interact with LRBA in both databases. To investigate this potential interaction, we performed co-immunoprecipitation (co-IP) and proximity ligation assays (PLA). Endogenous LRBA interacted with PIK3R4 by co-IP in HEK293T cells (Fig. 1B) and by PLA in lymphoblastoid B cells (LCL) from a healthy donor (Fig. 1C,D). Similarly, we detected a physical interaction between overexpressed Myc-tagged LRBA and His-tagged PIK3R4 in HEK293T cells (Fig. 1E).

**Table 1:** Predicted LRBA interactors by computational predictions.

N°	Gen	Protein code	Protein	Function	Database
1	CTLA4	P16410	Cytotoxic T-lymphocyte associated protein	Inhibitory receptor acting as a major negative regulator of T-cell responses	STRING
2	DCAF12L1	Q5VU92	DDB1- and CUL4-associated factor 12-like protein 1	Unknown	FuncBase
3	DCAF12L2	Q5VW00	DDB1- and CUL4-associated factor 12-like protein 2	Unknown	FuncBase
4	DCAF8L2	P0C7V8	DDB1- and CUL4-associated factor 8-like protein 2	Unknown	FuncBase
5	DOCK5	Q9H7D0	Dedicator of cytokinesis protein 5	Epithelial cell migration	FuncBase
6	DOCK8	Q8NF50	Dedicator of cytokinesis protein 8	Migration	STRING
7	FBXW7	Q969H0	F-Box and WD repeat domain containing 7	Ubiquitination and subsequent proteasomal degradation of target proteins	STRING
8	FBXW10	Q5XX13	F-box/WD repeat-containing protein 10	Ubiquitination and subsequent proteasomal degradation of target proteins	FuncBase
9	GNB1L	Q9BYB4	Guanine nucleotide-binding protein subunit beta-like protein 1	Intracellular signal transduction	FuncBase
10	IFT80	Q9P2H3	Intraflagellar transport protein 80 homolog	Development and maintenance of motile and sensory cilia	FuncBase
11	KIAA1875	A6NE52	WD repeat-containing protein 97	Generation of catalytic spliceosome	FuncBase
12	KIF21A	Q7Z4S6	Kinesin-like protein KIF21A	Microtubule-based movement	FuncBase
**13**	**LRRK2**	**Q5S007**	**Leucine-rich repeat serine/threonine-protein kinase**	**Autophagy through the CaMKK/AMPK**	**FuncBase**
14	LYST	Q99698	Lysosomal-trafficking regulator	Sorting endosomal resident proteins into late multivesicular endosomes	FuncBase
15	NBEAL1	Q6ZS30	Neurobeachin-like protein 1	Phospholipid binding	FuncBase
16	NSMAF	Q92636	Protein FAN	Ceramide metabolic process	FuncBase
17	NUP214	P35658	Nuclear pore complex protein Nup214	Nuclear export signal receptor activity	FuncBase
**18**	**PIK3R4**	**Q99570**	**Phosphoinositide 3-kinase regulatory subunit 4**	**Macroautophagy and late endosome to vacuole transport**	**FuncBase** STRING
19	PRKACA	P17612	Protein kinase cAMP-activated catalytic subunit alpha	Phosphorylation of substrates in the cytoplasm and the nucleus	STRING
20	PRKACB	P22694	Protein kinase cAMP-activated catalytic subunit beta	Phosphorylation of substrates in the cytoplasm and the nucleus	STRING
21	PRKACG	P22612	Protein kinase cAMP-activated catalytic subunit gamma	Phosphorylation of substrates in the cytoplasm and the nucleus	STRING
22	STXBP2	Q15833	Syntaxin binding protein 2	Intracellular vesicle trafficking and vesicle fusion with membranes	STRING
23	TLE6	Q9H808	Transducin-like enhancer protein 6	Positive regulation of neuron differentiation	FuncBase
24	TNFRSF1B	P20333	TNF receptor superfamily member 1B	Calcineurin- dependent activation of NF-AT, NF-kappa-B and AP-1	STRING
25	TTC7A	Q9ULT0	Tetratricopeptide repeat domain 7A	Regulator of phosphatidylinositol 4-phosphate (PtdIns(4)P) synthesis	STRING
**26**	**VPS8**	**Q8N3P4**	**Vacuolar protein sorting-associated protein 8 homolog**	**Endosomal vesicle fusion**	**FuncBase**
**27**	**VPS11**	**Q9H270**	**Vacuolar protein sorting-associated protein 11 homolog**	**Vesicle-mediated protein trafficking**	**FuncBase**
28	WDR88	Q6ZMY6	WD repeat-containing protein 88	Unknown	FuncBase
29	WDFY3	Q8IZQ1	WD repeat- and FYVE-containing protein 3	Aggrephagy	FuncBase
30	WDR46	O15213	WD repeat-containing protein 46	Scaffold component of the nucleolar structure	FuncBase
31	WDR87	Q6ZQQ6	WD repeat-containing protein 87	Unknown	FuncBase

List of the 31 potential LRBA interactors identified by FuncBase and/or STRING. Proteins are listed alphabetically. Proteins highlighted in bold were selected for validation by co-IP or PLA shown in Fig. 1 or EV1.

**Figure EV1:**

LRBA interacts with UVRAG but not with other members of the PIKIII complex. (**A**) Co-IP analysis revealed an interaction between LRBA and UVRAG in HEK293T WT cells transfected with Myc-LRBA and GFP-UVRAG plasmids. Immunoprecipitation was performed with anti-LRBA and immunoblotted for GFP. (**B**) PLA of LRBA and VPS34 (top) or Beclin-1 (bottom) in LCL cells from a healthy donor (HD) under resting conditions, revealing a lack of proximity. Nuclear DAPI staining is shown in blue. Scale bar=20 µm. (**C**) HEK293T WT cells were transfected with Myc-LRBA plasmid and/or Myc-VPS34/His-PIK3R4 plasmid (PIK3R4 blots are shown in Fig. 1E). Immunoprecipitation was performed with anti-LRBA and immunoblotted for VPS34. VPS34 and PIK3R4 were detected in the same experiment, therefore images of LRBA input and IP are similar as in Fig. 1E. (**D**) HEK293T WT cells were transfected with Myc-LRBA plasmid and GFP-ATG14L plasmid. Immunoprecipitation was performed with anti-LRBA and immunoblotted for GFP. (**E**) PLA showing the absence of proximity of LRBA with PI3Kdelta in LCL cells from a HD under resting conditions. Nuclear DAPI staining is shown in blue. Scale bar = 20 µm.

LRBA contains several different domains that are involved in protein-protein interactions (Fig. 1F, top) (Cullinane et al, 2013; Martinez Jaramillo and Trujillo-Vargas, 2018). To determine the domains of LRBA that interact with PIK3R4, we co-expressed Flag-tagged fragments of LRBA (F1 to F7) with His-tagged full-length PIK3R4 in HEK293T cells (Fig. 1F, top and right panel). By co-IP, we detected a strong interaction between His-PIK3R4 and fragment 3 (F3) of LRBA, which contains the central WD40 domain of LRBA (Fig. 1F,G).

To explore the LRBA-PIK3R4 interaction further, we used High Ambiguity Driven protein-protein DOCKing (HADDOCK), an integrative platform for the modeling of biomolecular complexes (Dominguez et al, 2003; van Zundert et al, 2016). Homology modelling of the individual WD40 domains of LRBA and PIK3R4 predicted the well-established β-propeller structure. The WD40 domain of PIK3R4 was predicted to form a single helix on the surface, which served as an interacting constraint for the docking protocol (Fig. 1H). In the best model, the exposed ɑ-helix of PIK3R4 interacts with the upper surface of the LRBA β-propeller via hydrophobic and charged interactions (Fig. 1I).

Beyond PIK3R4, the PI3K-III core complex contains VPS34 and Beclin-1 (McKnight et al, 2014). This core complex interacts with various adaptor proteins, such as ATG14L and UVRAG (Ohashi et al, 2019; Yan et al, 2009). Our modelling predicted that PIK3R4 could indirectly bridge LRBA to VPS34 and, in turn, the entire PI3K-III core complex (Fig. 1I). To test this, we performed co-IP experiments and found that Myc-LRBA interacts with GFP-UVRAG in HEK293T cells (Fig. EV1A), although we did not detect physical interactions between LRBA and VPS34, Beclin-1, or ATG14L (Fig. EV1B–D). In addition, endogenous LRBA did not interact with the PI3K family member PI3K-delta (Fig. EV1E), an essential protein for immune responses (Nunes-Santos et al, 2019). Altogether, these results suggest that LRBA interacts with PIK3R4 and UVRAG, which positively regulate the PI3K-III complex (Abrahamsen et al, 2012; Liang et al, 2008).

### LRBA deficiency reduces PI3K-III activity and WIPI2 and DFCP1 punctae

**Figure 2:**
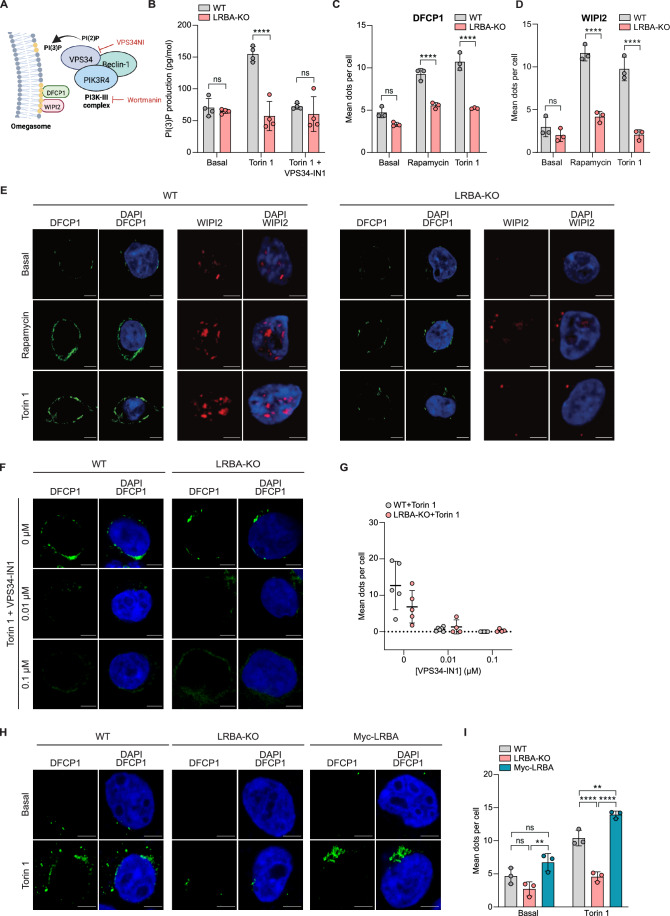
LRBA deficiency reduces PI(3)P production and WIPI2 and DFCP1 punctae. (**A**) Schematic representation of PI3K-III complex activity and the initiation of autophagosome formation (omegasome). (**B**) PI(3)P levels were measured in lipid extracts of WT (grey) and LRBA-KO (red) HEK293T cells under basal conditions, or upon treatment with 333 nM of Torin 1 alone, or in combination with 1 nM of VPS34-IN1 (PI3K-III complex inhibitor) for 4 h. Each dot represents technical replicates (*n* = 2) from one experiment, while bars represent the mean ± SD of *n* = 4 independent biological experiments. (**C**, **D**) Quantification of (**C**) DFCP1 and (**D**) WIPI2 signal in WT (grey) and LRBA-KO (red) HEK293T cells immunostained with either (**C**) anti-DFCP-1 or (**D**) anti-WIPI2 antibody after incubation for 1 h with either control medium, 50 µM Rapamycin, or 333 nM of Torin 1. Each dot represents the mean of one experiment while the bars represent the mean ± SD of *n* = 3 independent biological experiments. Total cells analyzed in (**C**, **D**) were: basal conditions, WT = 27/59, KO = 21/35; Rapamycin, WT = 44/70, KO = 49/91 and Torin 1, WT = 52/51, KO = 49/41. (**E**) Representative confocal microscopy images of WT (left) and LRBA-KO (right) HEK293T cells immunostained with anti-DFCP1 (green) or anti-WIPI2 (red) under the indicated conditions. Scale bar = 5 µm. (**F**) Representative confocal microscopy images (Scale bar=5 µm) and (**G**) quantification of DFCP1 signal in WT (grey) and LRBA-KO (red) HEK293T cells immunostained with anti-DFCP1 after treatment with the indicated concentrations of VPS34-IN1 for 1 h followed by 1 h incubation with 333 nM Torin 1. Each dot represents one image analyzed and the mean ± SD was calculated from n = 2 independent biological experiments. Total cells analyzed in WT: 0 μM = 46, 0.01 μM = 42, 0.1 μM = 42 and in KO: 0 μM = 29, 0.01 μM = 110, 0.1 μM = 94. Scale bar = 5 µm. (**H**, **I**) LRBA reconstitution restores DFCP1 recruitment. (**H**) Representative confocal microscopy images and (**I**) quantification of DFCP-1 expression in WT (grey), LRBA-KO (red) and Myc-LRBA (teal) HEK293T cells after stimulation for 1 h with control medium or 333 nM Torin 1. Each dot represents the mean of one experiment while bars represent the mean ± SD of *n* = 3 independent biological replicates. Total cells analyzed in WT (basal/Torin): 158/244 cells; LRBA-KO: 269/294 cells and Myc-LRBA: 152/273. Statistical analyses of (**B**–**D**) was performed using a two-way ANOVA with Bonferroni’s multiple comparisons test and for (**I**) a two-way ANOVA with Tukey’s multiple comparisons test, **P < 0.01 ((**I**): *P* = 0.0014, Basal LRBA-KO vs Myc-LRBA), ((**I**): *P* = 0.0037, Torin1 WT vs Myc-LRBA), ****P < 0.0001. Source data are available online for this figure.

The PI3K-III complex promotes the initiation and expansion of autophagosomes by generating PI(3)P, which is recognized by WIPI2 (WD-repeat protein Interacting with PhosphoInositides 2) and DFCP1 (Double FYVE domain-containing protein 1) proteins (Axe et al, 2008; Backer, 2016; Funderburk et al, 2010; Nascimbeni et al, 2017) (Fig. 2A). Given our observation that LRBA interacts with PIK3R4, we considered that LRBA deficiency would affect autophagy by altering PI3K-III complex activity. To test this, we induced autophagy by inhibiting mTORC1 with Torin 1 in wild-type (WT) and LRBA-KO HEK293T cells (Kim and Guan, 2015). Autophagy induction led to increased levels of PI(3)P in lipid extracts from WT but not from LRBA-KO HEK293T cells (Fig. 2B). As a positive control for PI3K-III complex inhibition, we used VPS34-IN1 to block VPS34 activity. This treatment effectively prevented the Torin 1-induced increase in PI(3)P levels in WT HEK293T cells, mimicking the PI3-III complex inhibition observed in LRBA-KO cells (Fig. 2B). Overall, these data suggest that LRBA deficiency reduces PI(3)P production during autophagy.

PI(3)P supports the translocation of the PI(3)P-binding proteins DFCP1 and WIPI2 into punctate compartments during autophagosome formation (Fig. 2A) (Axe et al, 2008; Palamiuc et al, 2020). WIPI2 and DFCP-1 accumulated in puncta in WT HEK293T cells after inducing autophagy by Torin 1 or another mTORC1 inhibitor, Rapamycin (Fig. 2C–E), as expected. In contrast, the number of DFCP1 and WIPI2 puncta were reduced in LRBA-KO cells compared to WT cells treated with mTORC1 inhibitors (Fig. 2C–E). VPS34-IN1 treatment dramatically reduced the number of DFCP1 puncta in WT HEK293T cells treated with Torin 1, consistent with PI3K-III inhibition, lower PI(3)P levels and reduced autophagy (Fig. 2F,G) (Jaber et al, 2012). VPS34-IN1 treatment had a smaller impact on DFCP1 puncta in LRBA-KO HEK293T cells treated with Torin 1. Importantly, DFCP1 puncta were restored in LRBA-KO cells transfected with Myc-LRBA (Figs. 2H,I and EV2A). Notably, expression levels of PIK3R4, VPS34, WIPI2 and DFCP1 were similar in WT and LRBA-KO HEK293T cells, irrespective of cells genotype or Torin 1 treatment (Fig. EV2B–F), confirming that loss of LRBA affects PIK3R4 activity and WIPI2/DFCP1 punctae but not protein expression.

**Figure EV2:**

Diminished DFCP1/WIPI2 punctae under starvation. (**A**) Representative immunoblot (left) and densitometry analyses (right) of LRBA expression in relation to Tubulin in WT (grey), LRBA-KO (red) and Myc-LRBA reconstituted (teal) HEK293T cells. Each dot represent one blot while bars represent the mean ± SD of *n* = 4 independent biological replicates. (**B**–**F**) Loss of LRBA does not affect the protein expression of PIK3R4, VPS34, DFCP-1 and WIPI-2. Representative immunoblot (**B**) and densitometry analysis of the protein levels of (**C**) PIK3R4, (**D**) VPS34, (**E**) DFCP1 and (**F**) WIPI2 in relation to Tubulin in the presence and absence of Torin 1, with or without Bafilomycin A1, for WT and LRBA-KO HEK293T cells. Each dot represents the densitometry analysis of one blot while bars represent the mean ± SD from *n* = 3–6 independent biological replicates. (**G**–**I**) (**G**) Quantification of WIPI2 and (**H**) DFCP1 expression in WT (grey) and LRBA-KO (red) HEK293T cells cultured for 1 h with EBSS or 100 nM Wortmannin. Each dot represents the mean of one experiment while bars represent the mean ± SD of *n* = 3 independent biological replicates. Total cells analyzed (WIPI2/DFCP1) for WT EBSS: 14/60 cells and KO = 28/42 cells. For WT=Wortmannin 29/57 cells and KO = 26/83 cells. (**I**) Representative confocal microscopy images of DFCP1 (green) and WIPI2 (red) signal upon staining with anti-DFCP1 and anti-WIPI2 antibodies. Scale bar=5 µm. Statistical analyses of (**A**) was performed using a one-way ANOVA with Tukey’s multiple comparisons test and for (**C**–**H**) a two-way ANOVA with Bonferroni’s multiple comparisons test, **P < 0.01 ((**G**): *P* = 0.0052), ***P < 0.001 ((**A**): *P* = 0.0006), ****P < 0.0001.

In addition, we observed severe reduction of WIPI2/DFCP1 punctae in LRBA-KO cells treated with mTORC1 inhibitors than under starvation (Figs. 2E and EV2G–I), suggesting that LRBA-KO cells were less sensitive to mTOR inhibition than WT cells. Indeed, phosphorylation of mTOR targets p70-S6K1 and S6RP were elevated in cells lacking LRBA compared to controls (Fig. EV3A–D), suggesting that mTOR activity is higher in the absence of LRBA. Reconstitution of LRBA-KO HEK293T cells with Myc- LRBA restored S6RP phosphorylation to WT cells levels (Fig. EV3E–G).

**Figure EV3:**

Enhanced mTOR signaling in LRBA-deficient cells. (**A**) Representative histogram of pS6K-MFI detected by flow cytometry in LCL from HD (grey) and LRBA-deficient patients (red) at basal levels. (**B**) Bar graphs representing the percentage of cells positive for pS6K in HD (grey) and two LRBA-deficient patients (red). Each dot represents the mean of *n* = 2 technical replicates while bars present the mean ± SD of *n* = 4 independent biological replicates. (**C**) Representative immunoblot analyses of LRBA, S6K total, S6K pT389 and GAPDH performed in shControl and shLRBA-HeLa cells at basal conditions. (**D**) Bar graphs represent densitometry analyses of S6K total and S6K pT389 expression from HeLa cells. Quantifications of protein expression were previously normalized to GAPDH and to shControl (dotted line). The bars represent the means ± SD from *n* = 4 independent biological replicates. (**E**–**G**) pS6RP levels were rescued after LRBA reconstitution. (**E**) Representative histogram of pS6RP-MFI (**F**) representative dot plot of p6RP+ cells, and (**G**) percentage of cells positive for pS6RP that was detected by flow cytometry in WT (grey), LRBA-KO (red) and Myc-LRBA (teal) at basal levels. Each dot represents the mean of *n* = 2 technical replicates while bars present the mean ± SD of *n* = 3 independent biological replicates. Statistical analyses of (**B**, **D**) was performed using a unpaired Welch’s *t* test and for (**G**) a one-way ANOVA with Tukey’s multiple comparisons test, *P < 0.05 ((**G**): *P* = 0.0144), **P < 0.01 ((**B**): *P* = 0.0073), ((**G**): *P* = 0.001).

Overall, these data suggest that LRBA deficiency reduces PI3K-III activity, leading to decreased PI(3)P levels and impaired recruitment of PI(3)P-binding proteins WIPI2 and DFCP1, potentially compromising autophagy flux.

### LRBA-deficient cells have impaired autophagosome-lysosome fusion and p62 degradation

**Figure 3:**
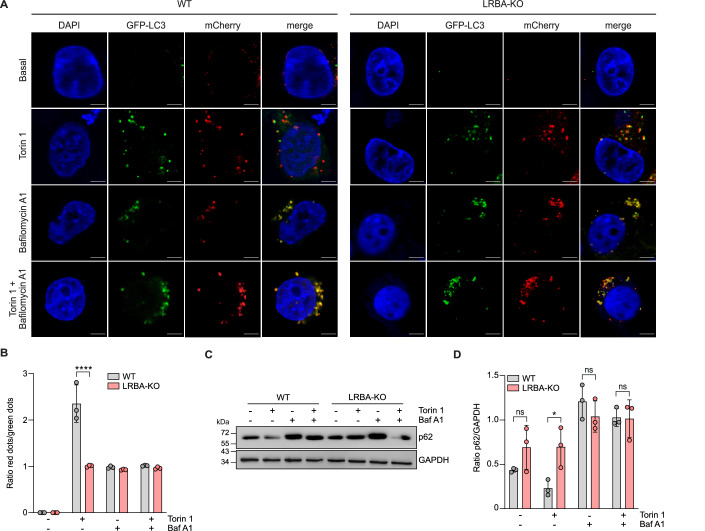
LRBA-deficient cells have impaired autophagosome-lysosome fusion and p62 degradation. (**A**) Representative confocal microscopy images of WT and LRBA-KO HEK293T cells stably transduced with mCherry-GFP-LC3 tandem vector after 4 h treatment with 333 nM Torin 1 alone, 200 nM Bafilomycin A1 alone, or in a combination of both. Scale bar = 5 µm. (**B**) Ratio red/green dots quantification from (**A**). Each dot represents the mean of one experiment while bars represent the mean ± SD of *n* = 3 independent biological replicates. Total cells analyzed in WT: Torin 1 = 87, Bafilomycin A1 = 86, Torin 1+Bafilomycin A1 = 86 cells; LRBA-KO: Torin 1 = 81, Bafilomycin A1 = 93, Torin 1+Bafilomycin A1 = 84 cells. (**C**) Representative immunoblot analyses of p62 expression in WT and LRBA-KO HEK293T cells at basal conditions or after 4 h stimulation with 333 nM Torin 1 alone, with 200 nM Bafilomycin A1 alone, or with a combination of both. GAPDH was used as housekeeping protein. (**D**) Densitometry analyses of p62 immunoblots in WT (grey) and LRBA-KO (red) HEK293T cells. Each dot represents the densitometry analysis of one blot while bars represent the mean ± SD from *n* = 3 independent biological replicates. Statistical analyses of (**B**, **C**) was performed using a two-way ANOVA with Bonferroni’s multiple comparisons test *P < 0.05 ((**D**): *P* = 0.0193), ****P < 0.0001. Source data are available online for this figure.

**Figure EV4:**
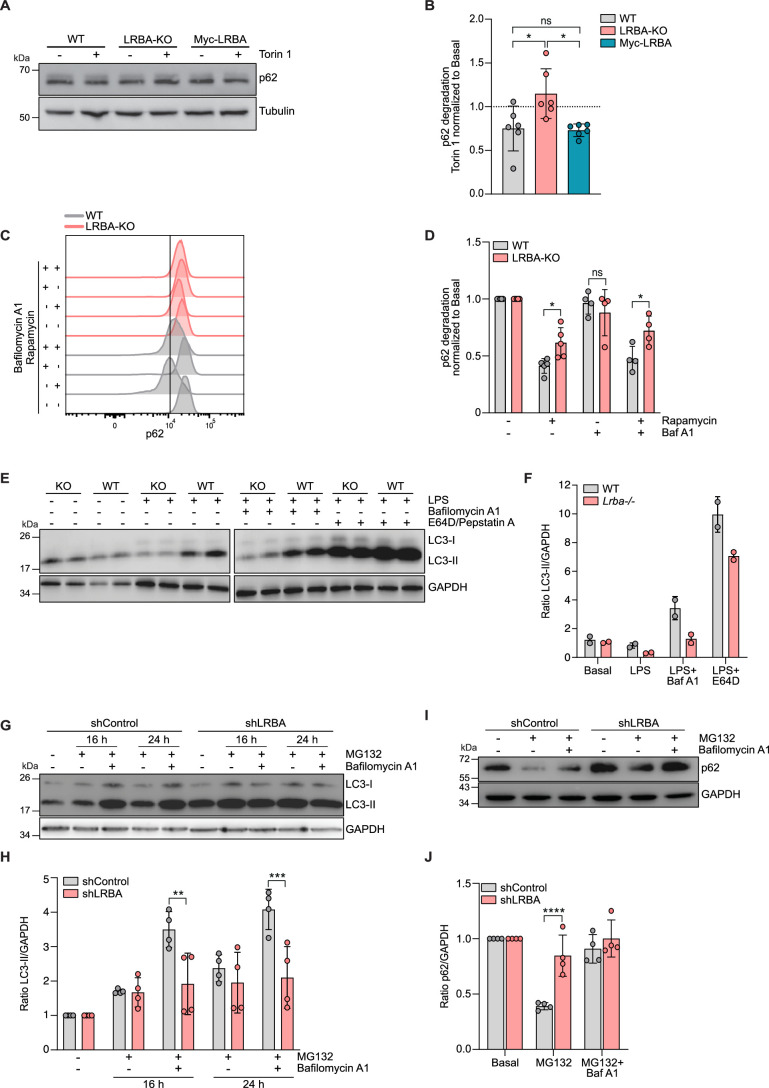
Abnormal autophagy flux in shLRBA HeLa cells and B cells from *Lrba−/−* mice. (**A**) Representative immunoblot analyses of p62 degradation in WT, LRBA-KO and Myc-LRBA HEK293T cells at basal conditions or after Torin 1 stimulation. (**B**) Densitometry analysis of (**A**) of WT (grey), LRBA-KO (red) and Myc-LRBA (teal) HEK293T cells. Each dot represents the densitometry analysis of one blot while bars represent the mean ± SD from *n* = 6 independent biological replicates. (**C**, **D**) Representative histograms of p62 degradation in WT (grey) and LRBA-KO (red) Ramos B cells in the presence and absence of Rapamycin, with or without Bafilomycin A1 and (**D**) fold change of p62 degradation normalized to basal levels. Each dot represents the fold change of one technical replicate while bars represent the mean ± SD from *n* = 4–5 independent biological replicates. (**E**, **F**) Reduced autophagy flux in splenic naive B cells from *Lrba*^*−/−*^ mice. (**E**) Representative immunoblot analyses of LC3-I and LC3-II processing and Tubulin in isolated splenocytes from WT and Lrba^−/−^ mice at day 0 or upon stimulation for 3 days with 20 µg/ml LPS alone or in the presence of 100 nM Bafilomycin A1 or protease inhibitors E64D and Pepstatin. (**F**) Densitometry analyses of LC3-II expression relative to Tubulin of splenic murine naive B cells from WT (grey) and *Lrba−/−* (red) mice. Each dot represents the densitometry analysis of one blot while bars represent the mean ± SD from *n* = 2 independent biological replicates. (**G**) Representative immunoblot analyses of the processing of endogenous unconjugated LC3-I to lipid-conjugated LC3-II in shControl and shLRBA HeLa cells at resting conditions or after 16 h or 24 h of MG132 alone or in the presence of 100 nM Bafilomycin A1. (**H**) Densitometry analyses of LC3-II expression relative to GAPDH of shControl (grey) and shLRBA (red) HeLa cells. Each dot represents the densitometry analysis of one blot while bars represent the mean ± SD from *n* = 4 independent biological replicates. (**I**) Representative immunoblot analyses of p62 and GAPDH in shControl and shLRBA HeLa cells at resting conditions or after 16 h treatment with MG132 alone or along with 100 nM Bafilomycin A1. (**J**) Densitometry analyses of p62 relative to GAPDH and normalized to basal conditions of shControl (grey) and shLRBA (red) HeLa cells. Each dot represents the densitometry analysis of one blot while bars represent the mean ± SD from *n* = 4 independent biological replicates. Statistical analysis for (**B**) was performed using a one-way ANOVA with Tukey’s multiple comparisons test and for (**D**, **H**, **I**) a two-way ANOVA with Bonferroni’s multiple comparisons test, *P < 0.05 (**B**: *P* = 0.02 WT vs KO and *P* = 0.0146 KO vs Myc-LRBA), (**D**: *P* = 0.0237 Rapamycin and *P* = 0.0132 Rapamycin+Bafilomycin A1), **P < 0.01 ((**H**): *P* = 0.0027), ***P < 0.001 ((**H**): *P* = 0.0002), ****P < 0.0001.

To determine whether LRBA is required for proper autophagic flux, we used HEK293T cells stably expressing the mCherry-GFP-LC3 tandem vector. This dual-tag system discerns early autophagosomes (green and red signal) from mature autolysosomes (red signal) (Lopez et al, 2018). Therefore, a high ratio of red foci/green foci reflects high autophagic flux. WT HEK293T cells expressing mCherry-GFP-LC3 and treated with Torin 1 showed a high ratio of red foci/green foci, which was reduced by inhibiting autophagic flux with Bafilomycin A1 treatment (Fig. 3A,B). In contrast, LRBA-KO HEK293T cells stimulated with Torin 1 displayed a low red foci/green foci ratio, which was unaffected by inhibiting autophagic flux with Bafilomycin A1 (Fig. 3A,B). Additionally, we monitored the levels of p62, a cargo protein that is degraded during autophagy (Lopez et al, 2018). We observed reduced levels of p62 in WT HEK293T cells following Torin 1 treatment, which was effectively blocked by treatment with Bafilomycin A1, as expected (Fig. 3C,D). In contrast, p62 levels did not change in LRBA-KO HEK293T cells treated with Torin-1 (Fig. 3C,D), and were rescued by LRBA reconstitution with Myc-LRBA (Fig. EV4A,B).

Abnormal autophagy flux was also observed in immune cells upon autophagy induction. Specifically, we observed reduced p62 degradation following Rapamycin treatment in LRBA-KO Ramos B cells (Fig. EV4C,D), and a lower LC3-II/GAPDH ratio after LPS stimulation in B cells from *Lrba−/−* mice, compared to their wild-type counterparts (Fig. EV4E,F). Similar results were obtained in human shLRBA HeLa cells upon autophagy induction with MG132 (Fig. EV4G–J) (Lagos et al, 2022). MG132 inhibits proteasome activity, while LPS induces B cell differentiation and enhances immunoglobulin production. Both, MG132 and LPS stimulation result in protein accumulation and cellular stress, which subsequently activate autophagy (Ding et al, 2007; He et al, 2021; Pengo et al, 2013).

Overall, these data set suggest that autophagic flux and autophagosome-lysosome fusion is reduced in LRBA-deficient cells, leading to an accumulation of autophagosomes.

### Loss of LRBA leads to accumulation of large autophagosomes

**Figure 4:**
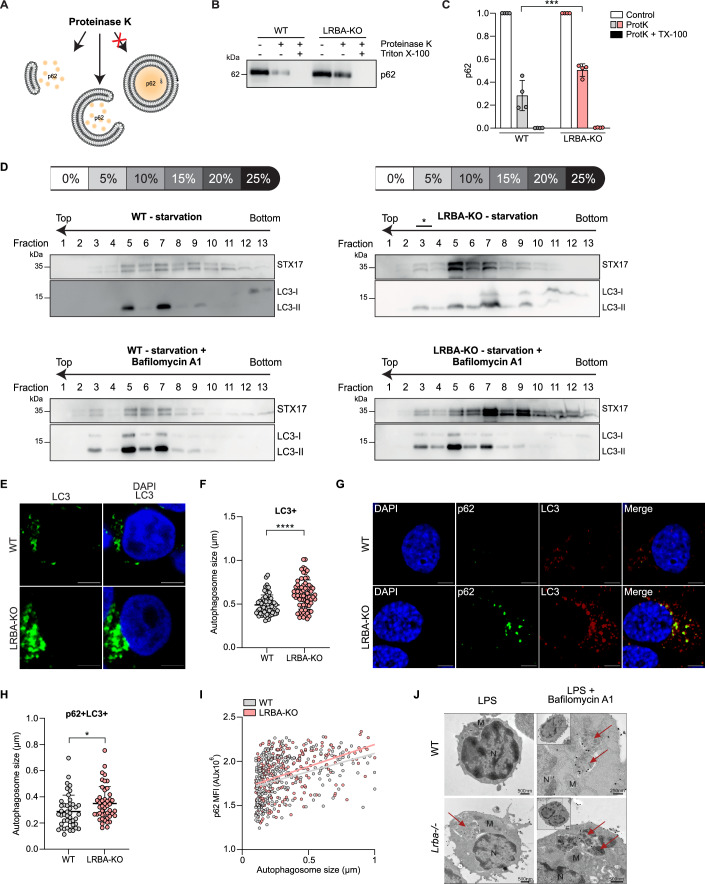
Loss of LRBA leads to abnormal accumulation of enlarged, sealed and unfused autophagosomes. (**A**) Schematic illustration of the protease protection assay. Cell homogenates are treated with proteinase K resulting in the degradation of exposed proteins but not membrane-enclosed proteins. (**B**) WT and LRBA-KO HEK293T cells were grown in EBSS medium with 300 nM Torin 1 for 2 h. After lysis, cell membranes were subjected to proteinase K and Triton X-100 treatment as indicated and analyzed by anti-p62 western blotting. (**C**) Quantification of (**B**) in WT (grey) and LRBA-KO (red) HEK293T cells. Each dot represents the densitometry analysis of one blot while bars represent the mean ± SEM from *n* = 4 independent biological replicates. (**D**) WT and LRBA-KO HEK293T cells were grown in EBSS medium with 300 nM Torin 1 for 3 h with or without 200 nM Bafilomycin A1, and cell homogenates were subjected to OptiPrep flotation analyses. The asterisk indicates the accumulation of autophagosomes in LRBA-KO homogenates. (**E**) Representative confocal microscopy images of LC3 signal in WT and LRBA-KO HEK293T cells stained with anti-LC3 after Bafilomycin A1 stimulation for 1 h. Scale bar = 5 µm. (**F**) Scatter plots showing the autophagosome size (in µm) of (**E**) in WT (grey) and LRBA-KO (red) HEK293T cells. Each dot represents one autophagosome from *n* = 3 independent biological replicates (total *n* = 68 autophagosomes), mean ± SD shown by the black line and error bars. (**G**) Representative confocal microscopy images of WT and LRBA-KO HaCat cells stimulated overnight with IFN-γ and treated with 20 µM chloroquine for 6 h. Fixed cells were stained for p62 (green), LC3 (red) and DAPI (blue) for the nuclei. Scale bar = 5 µm. (**H**) Scatter plots showing the autophagosome size (in µm) of (**G**) in WT (grey) and LRBA-KO (red) HaCat cells. Each dot represents the average size of all autophagosomes from one cell from *n* = 3 independent biological replicates with a total of at least 43 cells analyzed per condition, mean ± SD shown by the black line and error bar. (**I**) Correlation of autophagosome size with p62 MFI in WT (grey) and LRBA-KO (red) HaCat cells. Each dot represents one autophagosome from *n* = 3 independent biological replicates with a total of *n* = 262 (WT) and *n* = 225 (LRBA-KO) autophagosomes analyzed. (**J**) Electron microscopy images on LPS-stimulated B cells from WT and *Lrba*^−*/*−^ mice using immunogold labeling for LC3. Arrows point to autophagosomes, N: nucleus, M: mitochondria. Images represent higher magnification of the inset’s black-boxed area. A total of three mice per genotype were analyzed. Scale bars = 500 nm for LPS WT and *Lrba*^*−/−*^ and for *Lrba*^*−/−*^LPS+Bafilomycin A1, 250 nm for WT LPS+Bafilomycin A1. Statistical analyses of (**C**) was performed using a two-way ANOVA with Bonferroni’s multiple comparisons test, and for (**F**, **H**) a unpaired Welch’s *t* test, *P < 0.05 ((**H**): *P* = 0.0275), ***P < 0.001 ((**C**): *P* = 0.001), ****P < 0.0001. Source data are available online for this figure.

To test whether the autophagosomes that accumulated in LRBA-KO cells are mature and sealed, we monitored the sensitivity of p62 to proteinase K; p62 is protected from degradation when it is present in sealed autophagosomes (Abada et al, 2017) (Fig. 4A). We performed this protease protection assay on WT and LRBA-KO HEK293T cells treated with Torin 1. Interestingly, LRBA deficiency rendered p62 less sensitive to proteinase K treatment (Fig. 4B,C). These data suggest that autophagosomes in LRBA-deficient cells are sealed and accumulating cargo.

**Figure EV5:**
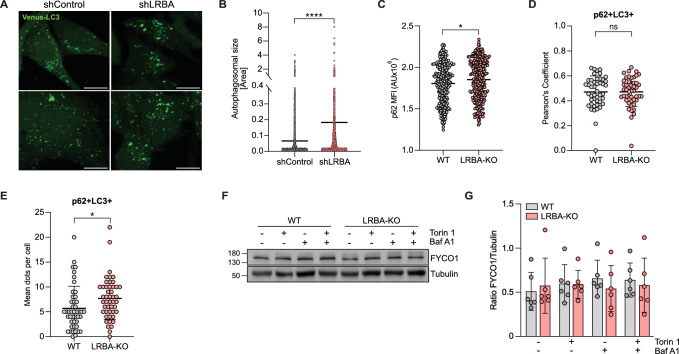
Loss of LRBA leads to enlarged autophagosomes. (**A**, **B**) shControl and shLRBA HeLa cells were transfected with GFP-LC3 plasmid and incubated for 2 h with 100 nM of Bafilomycin A1 for microscopy evaluation. (**A**) Representative confocal microscopy images of LC3-transfected shControl and shLRBA HeLa cells (green). Scale bar = 10 µm. (**B**) Size of GFP-LC3 punctae in shControl (grey) and shLRBA (red) HeLa cells was determined from binary images using the analyses particle module of FIJI, with a particle size from 0 to 20 µm^2^. Each dot represents an autophagosome from *n* = 3 fields across *n* = 3 independent biological replicates. Total autophagosomes for shControl = 961 and shLRBA= 1249. (**C**–**E**) Scatter plots showing the (**C**) p62 MFI in p62+LC3+ dots (**D**) co-localization of LC3 and p62 and (**E**) number of LC3+p62+ dots in WT (grey) and LRBA-KO (red) HaCat cells. Each dot for (**C**) represents one autophagosome from *n* = 3 independent biological replicates with a total of *n* = 262 (WT) and *n* = 225 (LRBA-KO) analyzed and for (**D**, **E**) one cell from *n* = 3 independent biological replicates with a total of at least 43 cells analyzed per condition, mean ± SD shown by the black line and error bars. (**F**) Representative immunoblot analyses and (**G**) densitometry analysis of FYCO1 and Tubulin expression upon basal conditions and stimulation with Torin 1 in presence or absence of Bafilomycin A1. Each dot represents the densitometry analysis of one blot while bars represent the mean ± SD from *n* = 6 independent biological replicates. Statistical analysis for (**B**–**E**) was performed using an unpaired Welch’s *t* test and for (**G**) two-way ANOVA with Bonferroni’s multiple comparisons test, *P < 0.05 ((**C**): *P* = 0.0258), ((**E**): *P* = 0.0304), ****P < 0.0001.

Additionally, we performed membrane flotation assays using OptiPrep gradients on starved HEK293T cells, (Matsui et al, 2018) and examined the gradients by detecting LC3-II on western blots. Autophagosomes typically float at 5% OptiPrep, whereas autophagic precursor membranes, endoplasmic reticulum (ER) and lysosomes float at higher densities. In starved LRBA-KO HEK293T cells, LC3-II was detected not only at the 5% and 7% fractions as in WT cells, but also in the 3% and 4% fractions, indicating an accumulation of enlarged autophagosomes (Fig. 4D). WT cells treated with Bafilomycin A1 also accumulated larger autophagosomes in the 3% and 4% fractions (Fig. 4D), phenocopying loss of LRBA. We obtained similar results by monitoring the autophagosomal protein STX17 (Fig. 4D). Enlarged and accumulating autophagosomes were also observed in LRBA-KO HEK293T and shLRBA HeLa cells versus their control cells by detecting LC3 signal (Figs. 4E,F and EV5A,B), and in LRBA-KO HaCat cells by LC3+p62+ co-staining (Fig. 4G,H). A positive correlation between autophagosome size and cargo content was observed, indicating that larger autophagosomes carry more cargo (Fig. 4I). Notably, LRBA-KO HaCat cells showed an overall higher p62 cargo compared to WT cells (Fig. EV5C). Although co-localization analysis for p62+LC3+ signals was similar between LRBA-KO and WT HaCat cells, we observed an increased accumulation of these vesicles in LRBA-deficient cells, supporting abnormal autophagosome-lysosome fusion (Fig. EV5D,E). In line with these results, LC3 immunogold labelling and electron microscopy (EM) also revealed larger autophagosomes in LPS-stimulated B cells from *Lrba*^−/−^ mice versus WT mice (Fig. 4J).

These data indicate that in the absence of LRBA, autophagosomes fail to fuse with lysosomes leading to the accumulation of large cargo-containing autophagosomes.

### LRBA binds to FYCO1 facilitating proper autophagosome movement and lysosome positioning

**Figure 5:**
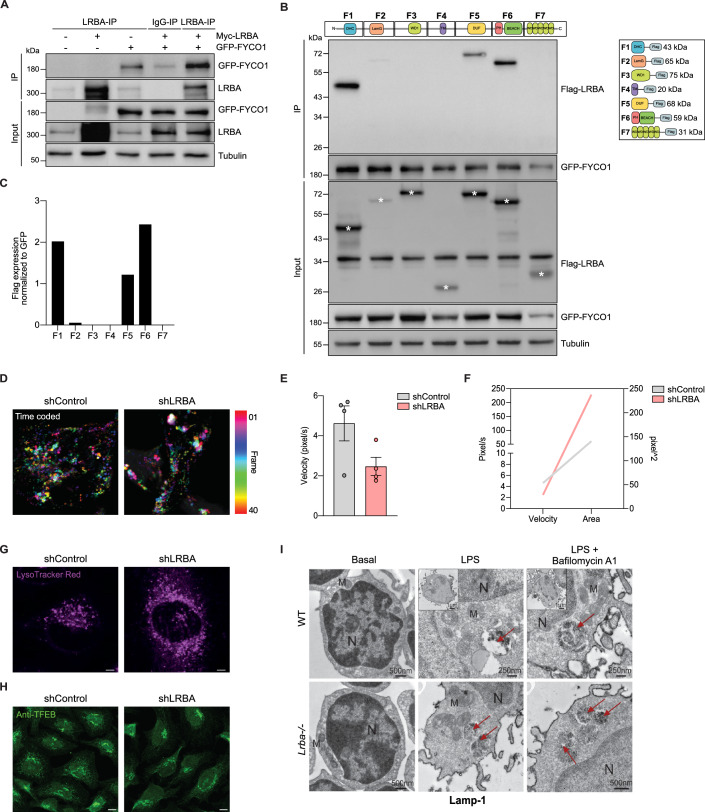
LRBA binds to FYCO1 facilitating proper autophagosome movement and lysosome positioning. (**A**) WT HEK293T cells were transfected with GFP-FYCO1 and/or Myc-LRBA plasmid. Pull down was performed with anti-LRBA and immunoblotted with anti-GFP. (**B**) WT HEK293T cells were co-transfected with GFP-FYCO1 plasmid and flag-tagged fragments 1 to 7 of LRBA protein domains. Flag-LRBA pull downs were immunoblotted with anti-GFP. Schematic representation of Flag-tagged plasmids containing different LRBA protein domains is shown on the right. (**C**) Ratio of Flag expression normalized to GFP expression of *n* = 2 independent biological replicates. (**D**) Mobility of autophagosomes was evaluated in LC3-GFP transfected shControl and shLRBA HeLa cells by time-lapse movies (Movies EV1 and EV2) acquired from individual frames, color-coded and projected into one image, using the temporal color-coding module of the Zeiss Zen Black software. (**E**) Quantification of autophagosomes velocity was performed using Manual Track under resting conditions. Each dot corresponds to one autophagosome tracked over 25 frames for shControl (grey) and shLRBA (red) HeLa cells transfected with LC3-GFP. Bars represent mean ± SD from *n* = 4 tracked autophagosome from *n* = 1 videos. (**F**) Correlation graph of velocity and area of the autophagosomes tracked in shControl (grey) and shLRBA (red) HeLa cells. (**G**) Lysosomes from shControl and shLRBA HeLa cells under resting conditions were stained with LysoTracker red. Scale bar = 5 µm. (**H**) Representative fluorescent microscopy images of shControl and shLRBA cells stained with anti-TFEB upon resting conditions. Scale bar = 5 µm. (**I**) EM images of LPS-stimulated B cells from WT and *Lrba*^−*/−*^ mice using immunoperoxidase labeling for Lamp-1. Arrows point to lysosomes. N nucleus, M mitochondria. Images represent higher magnification of the inset’s black-boxed area. A total of three mice per genotype were analyzed. Scale bar = 500 nm for WT Basal and *Lrba*^*−/−*^ Basal, and *Lrba*^*−/−*^ LPS and LPS+Bafilomycin A1. 250 nm for WT LPS and LPS+Bafilomycin A1. Source data are available online for this figure.

In addition to the interaction between LRBA and regulators of the PI3K-III complex that we discovered here, LRBA was predicted to interact with FYCO1 in previous studies (Behrends et al, 2010). FYCO1 supports autophagosome transport and maturation by interacting with kinesin motor proteins and with autophagosome components (Mackeh et al, 2013; Pankiv et al, 2010; Pankiv and Johansen, 2010). We validated the potential interaction of LRBA with FYCO1 by co-IP in HEK293T cells co-transfected with GFP-tagged FYCO1 and Myc-tagged LRBA (Fig. 5A). In addition, we identified three fragments of LRBA that are sufficient to interact with FYCO1 (Fig. 5B,C). Notably, FYCO1 protein levels remain unaltered in LRBA-KO HEK293T compared to WT HEK293T cells, both before and after stimulation with Torin 1 (Fig. EV5F,G), suggesting that loss of LRBA does not affect FYCO1 stability.

To investigate whether LRBA deficiency affects autophagosome transport, we performed live-microscopy of HeLa cells expressing GFP-LC3 treated with Bafilomycin A1. We found that autophagosomes have a reduced velocity in shLBRA HeLa cells compared to shControl HeLa cells (Fig. 5D,E; Movies EV1 and EV2). Velocity was directly proportional to the autophagosome size (Fig. 5F).

Interestingly, LysoTracker Red staining revealed that lysosomal positioning was also altered in shLRBA HeLa cells compared to shControl cells (Fig. 5G). However, the levels and localization of Transcription Factor EB (TFEB), the master regulator of lysosomal biogenesis, were unaltered in shLRBA HeLa cells (Fig. 5H), suggesting that lysosome biogenesis is unaffected. EM analysis of lysosomes via Lamp-1 immunogold labelling revealed an accumulation of lysosomes in LPS-stimulated B cells from *Lrba*^−/−^ mice compared to WT (Fig. 5I).

Our data indicate that LRBA interacts with the PI3K-III complex and FYCO1, coordinating autophagosome maturation and transport, and supporting efficient autophagic flux.

### LRBA deficiency enhances MHC class II restricted antigen presentation and T-cell responses

Autophagy has long been associated with immune recognition and responsiveness (Crotzer and Blum, 2009). In particular, it has been identified as a route by which cytoplasmic and nuclear antigens are degraded and loaded onto MHC-II molecules (e.g. HLA-DR) via fusion of autophagosomes with MIIC vesicles in professional and non-professional antigen presenting cells (Arbogast et al, 2019; Ljunggren and Anderson, 1998; Munz, 2016; Runsala et al, 2023). The resulting peptide-MHC-II complexes are then transported to the cell surface and presented to CD4^+^ T cells, triggering cytokine production and the activation of an adaptive immune response (Munz, 2021).

**Figure 6:**
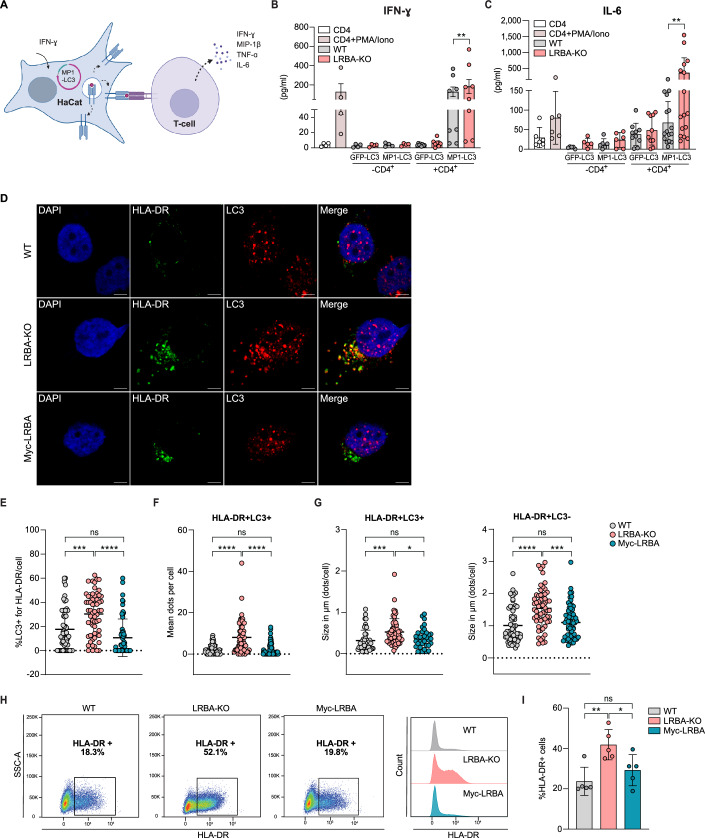
LRBA deficiency enhances MHC class II restricted antigen presentation and T cell responses. (**A**) Schematic illustration of the antigen presentation assay. WT or LRBA-KO HaCat cells stably expressing LC3-GFP or MP1-LC3 (target cells), and pre-treated with IFN-γ for 24 h to up-regulate MHC-II molecules were cultured with a MP1-specific CD4^+^ T cell clone (effector cells) from a Healthy Donor for 20 h. Following incubation, proinflammatory cytokines were measured in the culture supernatants. (**B**, **C**) ELISA assays on culture supernatants from co-cultures of an T-cell effector to HaCat target cell (E:T = 10:5) ratios as described in (**A**). Each dot represents two technical replicates while bars represent the mean ± SD of (**B**) IFN-γ (*n* = 4 individual biological replicates) and (**C**) IL-6 (*n* = 4 individual biological replicates) secretion in WT (grey) and LRBA-KO (red) HaCat cells. (**D**) Representative confocal microscopy images of WT, LRBA-KO and Myc-LRBA reconstituted HaCat cells stimulated overnight with IFN-γ and treated with 20 µM chloroquine for 6 h. Fixed cells were stained for HLA-DR (green), LC3 (red) and DAPI (blue) for the nuclei. Scale bar = 5 µm. (**E**–**G**) Scatter plots represent the (**E**) percentage of LC3^+^ dots also positive for HLA-DR per cell, (**F**) the number of dots positive for HLA-DR and LC3 (known as MIIC vesicles), and (**G**) the average size in µm of HLA-DR+/LC3+ and HLA-DR+/LC3- in WT (grey) LRBA-KO (red) and Myc-LRBA (teal) HaCat cells. Each dot represents one cell from *n* = 3 individual biological replicates with a total of at least 30 cells analyzed per condition, mean ± SD shown by the black line and error bars. Quantification of LC3, HLA-DR or double positive dots per cell was performed using a semiautomatic plugin designed on FIJI. (**H**, **I**) HLA-DR expression in HaCat cells. (**H**) Representative dot plots of HLA-DR expression in WT, LRBA-KO and Myc-LRBA HaCat cells. (**I**) WT (grey), LRBA-KO (red) and Myc-LRBA (teal) HaCat cells positive for HLA-DR. Dots represent the mean of *n* = 2 technical replicates while bars represent the mean ± SD of *n* = 5 independent biological replicates. Statistical analysis of (**B**, **C**) was performed using a ratio paired student t-test and for (**E**–**G**, **I**) a one-way ANOVA with Tukey’s multiple comparisons test, *P < 0.05 ((**G**) HLA-DR+LC3+: *P* = 0.0483), ((**I**): *P* = 0.0471), **P < 0.01 ((**B**): *P* = 0.0022), ((**C**): *P* = 0.0013), ((**I**): *P* = 0.0058), ***P < 0.001 ((**E**): *P* = 0.0002), ((**G**): HLA-DR+LC3+: *P* = 0.0008), ((**G**): HLA-DR+LC3−: *P* = 0.0004), ****P < 0.0001. Source data are available online for this figure.

Given our data that LRBA is essential for autolysosome formation, we considered that LRBA deficiency would impact antigen presentation mediated by autophagy. To test this, we evaluated MHC-II-mediated antigen presentation in vitro. It was previously shown that an influenza matrix protein 1-LC3 (MP1-LC3) fusion protein is targeted to autophagic membranes, enhancing its degradation in MIICs and its MHC-II presentation to CD4^+^ T cell clones (Schmid et al, 2007). We leveraged this assay by expressing either GFP-LC3 or MP1-LC3 in WT and LRBA-KO HaCat cells (target cell), pre-stimulated them with IFN-γ, and then co-cultured them with or without MP1-specific CD4^+^ T cells from healthy donors (effector cells). To monitor productive antigen presentation on HaCat cells, we assessed the concentrations of IFN-γ, IL-6, TNF-α and MIP-1β secreted from T cells into the supernatant after 24 h of co-culture (Fig. 6A).

**Figure EV6:**
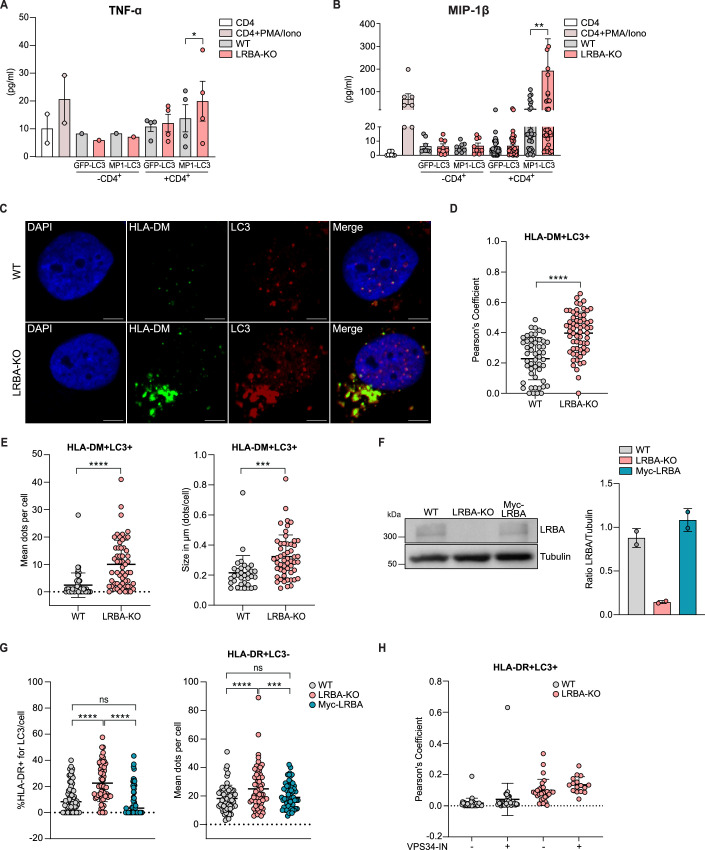
Loss of LRBA leads to increased TNF-α and MP-1β release *via* autophagy. (**A**, **B**) WT and LRBA-KO HaCat cells stably expressing GFP-LC3 or MP1-LC3 (target cells), and pre-treated with IFN-γ for 24 h to up-regulate MHC-II were cultured with a MP1-specific CD4^+^ T cell clone (effector cells) from a HD for 20 h. Following incubation, TNF-α and MIP-1β were measured in the culture supernatants by ELISA. Each dot represents one technical replicate while bars represent the mean ± SD of (**A**) TNF-α (*n* = 4 independent biological replicates for HaCat GFP/MP-1-LC3 cells in presence of CD4+ T cells, whereas *n* = 1 for HaCat GFP/MP-1-LC3 without CD4+ T cells, and *n* = 2 for CD4+ cells alone) and (**B**) MIP-1β (*n* = 4 independent biological replicates) secretion in WT (grey) and LRBA-KO (red) HaCat cells (**C**) Representative confocal microscopy images of WT and LRBA-KO HaCat cells stimulated overnight with IFN-γ and treated with 20 µM chloroquine for 6 h. Fixed cells were stained for HLA-DM (green), LC3 (red) and DAPI (blue) for the nuclei. Scale bar = 5 µm. (**D**, **E**) Scatter plots showing the (**D**) co-localization of LC3 and HLA-DM and (**E**) the number of dots per cell (left) and size (right) of vesicles positive for HLA-DM and LC3 in WT (grey) and LRBA-KO (red) HaCat cells. Each dot represents one cell from *n* = 3 independent biological replicates with a total of at least 35 cells analyzed per condition, mean ± SD shown by the black line and error bars. (**F**) Representative immunoblot and densitometry analyses of LRBA expression in WT (grey), LRBA-KO (red) and Myc-LRBA (teal) reconstituted HaCat cells. Each dot represents the densitometry analysis of one blot while bars represent the mean ± SD from *n* = 2 independent biological replicates (**G**) Scatter plots showing co-localization of HLA-DR and LC3 (left) and number of HLA-DR+LC3- dots (right) in WT (grey), LRBA-KO (red) and Myc-LRBA (teal) reconstituted HaCat cells. Each dot represents one cell from *n* = 3 independent biological replicates with a total of at least 30 cells analyzed per condition, mean ± SD shown by the black line and error bars. (**H**) Scatter plots showing the co-localization of HLA-DR and LC3 in unstimulated WT (grey) and KO (red) HaCat cells with or without VPS34 inhibitor (0.1 µM) treatment. Each dot represents one cell from *n* = 2 biological replicates with a total of at least 30 cells analyzed per condition, mean ± SD shown by the black line and error bars. Statistical analyses of (**A**, **B**) was performed using a ratio paired Student *t* test, for (**D**, **E**) a unpaired Welch’s *t* test, for (**G**) a one-way ANOVA with Tukey’s multiple comparisons test and for (**H**) and a two-way ANOVA with Bonferroni’s multiple comparisons, *P < 0.05 ((**A**): *P* = 0.0410), **P < 0.01 ((**B**): *P* = 0.0014), ***P < 0.0001 ((**E**): *P* = 0.0003), ((**G**): *P* = 0.0004), ****P < 0.0001.

We observed elevated cytokine levels after co-culture of MP1-LC3 WT HaCat cells with CD4^+^ T cells, which was absent when cultured alone or with GFP-LC3 WT HaCat cells (Figs. 6B,C and EV6A,B). Interestingly, we observed higher cytokine concentrations when T cells were co-cultured with MP1-LC3 LRBA-KO HaCat cells compared to MP1-LC3 WT HaCat cells (Figs. 6B,C and EV6A,B). These data suggest that MHC-II antigen presentation is potentially elevated in HaCat cells lacking LRBA.

To corroborate these findings, we detected the MIIC markers LC3 and HLA-DR in stimulated HaCat cells by fluorescence microscopy. Compared to WT HaCat cells, LRBA-KO HaCat cells displayed a higher number of MIIC vesicles (Fig. 6D,E), and an increased co-localization of HLA-DR and LC3 (Fig. 6D–F). In addition, HLA-DR-positive vesicles were significantly larger in LRBA-KO HaCat cells compared to WT cells (Fig. 6G). Similar results were obtained when using the MIIC marker, HLA-DM (Fig. EV6C–E). Reconstitution of LRBA-KO HaCat cells with Myc-LRBA restored the number and size of MIIC vesicles, as well as co-localization of HLA-DR and LC3 markers, to WT levels (Figs. 6E–G and  EV6F,G). Of note, untreated HaCat cells revealed increased co-localization of HLA-DR and LC3 in LRBA-KO HaCat cells already at basal levels, which was not modified after VPS34 inhibition (Fig. EV6H).

Flow cytometry analysis confirmed the elevated surface presentation of HLA-DR molecules in IFN-γ-stimulated HaCat cells lacking LRBA. This elevation was restored to WT levels upon ectopic expression of Myc-LRBA (Fig. 6H,I).

These data suggest that LRBA deficiency leads to an accumulation of enlarged antigen-containing MIIC vesicles, resulting in enhanced MHC-II-restricted antigen presentation of autophagy substrates to T cells. This, in turn, results in increased production of proinflammatory cytokines driven by T-cell activation.

## Discussion

In this study, we show that LRBA interacts with PIK3R4 to promote PIK3-III activity and autophagic flux. Thus, LRBA deficiency impairs autophagy and leads to an accumulation of enlarged autophagosomes. Similarly, we found that LRBA deficiency leads to an accumulation of MIIC vesicles, which was associated with increased MHC-II restricted antigen presentation and proinflammatory T-cell mediated responses in vitro. We propose that these functions of LRBA contribute to the hyperinflammation seen in LRBA-deficient patients, distinct from aberrant CTLA-4 trafficking. Together, these mechanisms would be expected to increase the severity of LRBA deficiency when compared to CTLA-4 insufficiency (Lo et al, 2016; Taghizade et al, 2023). However, future analyses will be required to disentangle the contribution of each mechanism to the hyperinflammatory disease phenotype arising from LRBA deficiency.

Our data suggest that the LRBA-PIK3R4 interaction occurs via WD40-WD40 domain binding (Jain and Pandey, 2018). WD40 domains are common protein-protein interaction domains, and the WD40 domain in WDFY3/ALFY enable its interaction with PI3K-III complexes and, in turn it participates in cargo selection and delivery to autophagosomes (Cullinane et al, 2013; Isakson et al, 2013). Only PIK3R4 harbours a WD40 domain within the PI3K protein family (Bilanges et al, 2019). Our data also suggest that the BEACH domain of LRBA, which is involved in the trafficking of CTLA-4-containing vesicles in Tregs (Lo et al, 2015), interacts with FYCO1. These data suggest that LRBA forms distinct protein complexes through its BEACH and WD40 domains, playing a role in both CTLA-4 recycling (Lo et al, 2015) and autophagy. It will be of interest to determine whether LRBA protein-protein interactions are cell- or stimuli-specific.

LRBA-KO cells showed reduced PI(3)P levels after autophagy induction. However, both WT and LRBA-KO cells produced similar levels of PI(3)P in resting cells, suggesting that the PI(3)P constitutive pool is not affected by the loss of the LRBA-PIK3R4 interaction. Indeed, downregulation of VPS34 expression in resting glioblastoma cells resulted in a residual production of PI(3)P (Johnson et al, 2006), indicating that the PI(3)P constitutive pool is generated independently of the PI3K-III complex. (Falasca and Maffucci, 2009; Gaullier et al, 2000; Johnson et al, 2006). VPS34-KO cells showed normal vesicle trafficking between the *trans*-Golgi and late endosomes, normal endocytic uptake of fluid-phase markers, and normal association with early endosomes, but disrupted late endosomal trafficking (Sarbassov et al, 2004). Therefore, the effect of LRBA deficiency in CTLA-4 vesicle trafficking could also be related to an aberrant activity of the PI3K-III complex. Moreover, we demonstrated that LRBA interacts with UVRAG, which together with PIK3R4 and VPS34 forms a protein complex located at the membrane of early endosomes acting in endocytosis, cytokinesis and lysosome recycling, suggesting that LRBA might also affect other vesicle trafficking processes. PI(3)P acts as a membrane-bound localized signal, controlling the assembly of PI(3)P-binding scaffold proteins such as WIPI2 and DFCP1 that mediate autophagosome biogenesis (Marat and Haucke, 2016). WIPI2 is necessary for recruiting the lipidation machinery for the phagophore-forming protein LC3B (Dooley et al, 2014; Polson et al, 2010), whereas the ATPase DFCP1 is necessary for releasing the newly formed autophagosomes from the ER (Nahse et al, 2023). LRBA-KO cells and WT cells displayed comparable levels of LC3 lipidation, despite reduced WIPI2 and DFCP1 puncta in LRBA-KO cells ruing autophagy. Normal autophagosome formation was also seen in DFCP1-mutant cells and in Vps34-mutant cells, despite reduced WIPI2 puncta (Bilanges et al, 2017). Similar to our observations, shVPS34 glioblastoma cells as well as *vps34*-KO MEF cells showed enlarged late endosomes but with an intact capacity to fuse with lysosomes (Johnson et al, 2006). Our data, however, suggest a delay of autophagosome/lysosome fusion in LRBA-KO cells, which highlights the additional role played by LRBA in the autophagy process.

We suggest that the LRBA-FYCO1 interaction is essential for autophagosome mobilization and regular lysosome positioning followed by autophagosome/lysosome fusion. This notion is reinforced by previous reports showing the importance of FYCO1 for intracellular transport of autophagic vesicles (Chen et al, 2011; Pankiv et al, 2010; Pankiv and Johansen, 2010), lysosomes and phagosomes (Mrakovic et al, 2012; Pu et al, 2016). In fact, FYCO1 depletion led to an accumulation of autophagosomes as observed in LRBA-KO cells (Behrends et al, 2010). Since ectopic expression of Myc-LRBA in LRBA-KO HEK293T cells rescued the recruitment of DFCP1 and the degradation of p62, our data support the requirement of LRBA for autophagosome maturation and therefore autophagy flux. Intriguingly, LRBA plays a role in ATG9A vesicle trafficking to mitochondria in HeLa cells and loss of LRBA resulted in reduced mitophagy efficiency (Nguyen et al, 2021).

We previously reported defective autophagy in naive B cells from LRBA-deficient patients, which was associated with poor cell survival, reduced plasmablast differentiation and low antibody production (Lopez-Herrera et al, 2012). The importance of autophagy for plasmablast survival and differentiation has been demonstrated in Atg5-deficient mice (Pengo and Cenci, 2013; Pengo et al, 2013). In T lymphocytes, the PI3K-I complex plays a key role in autophagy upon starvation and T cell receptor stimulation, whereas PI3K-III complex activity is dispensable (McLeod et al, 2021). However, PI3K-III complex-dependent autophagy is required for naive T-cell homeostasis through the quality control of mitochondria (Willinger and Flavell, 2012). Together, these observations may explain the impaired activation of mTORC1 and mTORC2 complexes in human LRBA-deficient Tregs (via PI3K-I complex) and the skewed T-cell memory phenotype (via PI3K-III complex) in LRBA deficiency (Charbonnier et al, 2015). LRBA-deficient patients have normal numbers of circulating T cells, suggesting that abnormal autophagy does not affect T-cell survival, but it nevertheless might affect endocytic trafficking routes.

Previous findings showed that the LC3-lipid and Atg12-Atg5 systems are required in HaCat cells for targeting MP1-LC3 fusion proteins for MHC class II loading and enhanced MHC class II presentation to CD4^+^ T-cells (Schmid et al, 2007). Using this setup in LRBA deficiency, HaCat cells showed enlarged and augmented number of MIIC vesicles potentially associated to an autophagosome rerouting towards MIIC vesicles as a cause of an autophagosome-lysosome fusion defect. Similar autophagosome enlargement and accumulation of the autophagy cargo protein p62 was also observed in LRBA-KO HEK293T and shLRBA HeLa cells. Ectopic expression of WT-LRBA in LRBA-KO cells restored MIIC vesicle sizes and number to those observed in WT cells.

MP1-specific CD4^+^ T-cell clones co-cultured with LRBA-KO HaCat cells displayed increased proinflammatory cytokine secretion, indicating enhanced CD4^+^ T-cell activation. These observations are in line with the clinical picture of LRBA-deficient patients, which is characterized by T-cell immune dysregulation (Alkhairy et al, 2016; Azizi et al, 2017a; Gamez-Diaz et al, 2016). An enhanced proinflammatory response in the context of LRBA deficiency has also been reported for *Lrba*^*−/*−^ mice, which display increased susceptibility to DSS-induced colitis (Wang et al, 2019). Dendritic cells from *Lrba*^*−/−*^ mice exhibited excessive IRF3/7- and PI3K/mTORC1-dependent signaling and type I IFN production in response to the stimulation of the Toll-like receptors (TLRs) 3, TLR7, and TLR9. It would however be valuable to determine whether FYCO1 dysfunction alone leads to enhanced cargo in MIIC vesicles and antigen presentation, which could clarify whether the observed MIIC accumulation is primarily dependent on FYCO1 or if it is solely due to LRBA loss.

Finally, we show that LRBA-deficient cells exhibit concomitant high mTORC1 activity potentially contributing to defective autophagy (Egan et al, 2011). Thus, mTORC1 inhibitors may be valuable treatment options for LRBA-deficient patients (Brachmann et al, 2009; Goodman et al, 2014). In fact, several clinical reports on patients with LRBA deficiency demonstrated that treatment with Sirolimus, an mTOR inhibitor, improved their clinical symptoms (Azizi et al, 2017b; Gamez-Diaz et al, 2016; Seidel et al, 2018).

In conclusion, our data suggest that LRBA facilitates autophagy by interacting with PIK3R4 and FYCO1. Loss of LRBA resulted in low PI(3)P production, reduced autophagosome-lysosome fusion, enlarged autophagosomes, and inefficient degradation of cargo proteins via autophagy. Accumulation of cargo/antigenic peptides, particularly in MIIC vesicles that contain MHC-II molecules, was associated with enhanced antigen presentation in LRBA-deficient cells, leading to a stronger T-cell driven proinflammatory immune response.

## Methods

**Table:** Reagents and tools table

Reagent/resource	Reference or source	Identifier or catalog number
**Experimental models**		
HEK 293T cell (*H. sapiens*)	ATCC	CRL-11268
HaCat cells (*H. sapiens*)	Kind gift from Prof. Rajiv Khanna, Brisbane, Australia.	Generated in the laboratory of Prof. Norbert E. Fusenig (J. Cell Biol. 1998)
HeLa cells (*H. sapiens*)	ATCC	CCL-2
LCL (Lymphoblastoid cell line (*H. sapiens*)	Generated in our laboratory using Healthy donor cells and Epstein Barr Virus supernatant	N/A
MP1-specific CD4+ T cell clone	Generated in the laboratory of Prof. Christian Münz, University of Zürich, Switzerland	
Ramos B cells	ATCC	CRL-1596
C57BL/6 N *Lrba −/−*	Generated in the laboratory of Prof. Manfred Kilimann. Max-Planck Institute Göttingen, Germany	C57BL/6N-Lrba^tm1.1Kili^/Ieg RRID:IMSR_EM:14924
Naive B cells (*M. musculus*)	From C57BL/6 N WT and Lrba−/− mice	N/A
**Recombinant DNA**		
pmCherry-GFP-LC3	Dr. Ian Gentle University of Freiburg, Germany	N/A
pClneo FLAG	Promega	E1841
Myc-DDK LRBA	Origene	RC17204
pVitro-hygro-N-myc-hVps34/Vps15-C-V5-his	Addgene	24296
pEGFP-Atg14L	Addgene	21635
pEGFP-C1-hUVRAG	Addgene	24296
pClneo FLAG LRBA fragments	Dr. Bernice Lo National Institute of Health, USA	N/A
pGFP-FYCO1	Dr. Christian Behrends University of Frankfurt, Germany	N/A
GFP-LC3	Prof. Christian Münz, University of Zürich, Switzerland	N/A
MP1-LC3	Prof. Christian Münz, University of Zürich, Switzerland	N/A
**Antibodies**		
Rabbit anti-LC3	Cell Signaling	E5Q2K
Mouse anti-Lamp1	Invitrogen	MA1-205
Mouse anti-WIPI2	Abcam	ab105459
Rabbit ZFYVE/DFCP1	LS-bio	LS-B15646-50
Rabbit anti-GAPDH	Cell Signalling	2118
Mouse anti-FLAG	Sigma-Aldrich	SAB4301135
Mouse anti-GFP	Santa Cruz	SC-9996
Mouse anti-AMPK total	Cell Signaling	2793S
Rabbit anti-pAMPK (T172)	Cell Signaling	2535S
Rabbit anti-LC3	Novus	NB110-2220
Rabbit anti-LRBA	Sigma-Aldrich	HPA023597
Rabbit anti-Myc	Cell Signaling	2278S
Mouse anti-PIK3R4	Novus	H00030849-M02
Rabbit anti-p62	Enzo Lifescience	5114S
Mouse anti-p62 AF647	Abcam	ab194721
Rabbit anti-p70S6K total	Cell Signaling	2708S
Mouse anti-p-p70S6K (T389)	Cell Signaling	9206S
Mouse anti-HLA-DR	Cell signaling	NB-100-7785
Mouse anti-HLA-DM AF488	Santa Cruz	sc-32248 AF488
Rabbit anti-FYCO1	Santa Cruz	25730-1-AP
Rabbit anti-P6RP	Cell signaling	4858
Rabbit anti-VPS34	Novus	NB110-87320SS
Mouse anti-HLA-DR FITC	Invitrogen	A16118
Rabbit anti-pS6RP Pacific Blue	Cell signaling	8520S
Anti-rabbit IgG-FITC	Invitrogen	A16118
Anti-rabbit AF488	Cell Signaling	4412
Anti-rabbit AF555	Cell Signaling	4413
Anti-mouse AF647	Cell Signaling	4410
HRP-linked anti-mouse	Santa Cruz	sc-516102
HRP-linked anti-rabbit	Invitrogen	31470
HRP-linked anti-Tubulin	Prointech	HRP-66031
PLA probe anti-mouse minus	Sigma-Aldrich	DU92004
PLA probe anti-goat minus	Sigma-Aldrich	DU92006
PLA probe anti-rabbit plus	Sigma-Aldrich	DUO92101
**Oligonucleotides and other sequence-based reagents**		
gRNA for CrispR	IDT	N.A.
shControl	Thermo Fisher	
shLRBA	Thermo Fisher	
**Chemicals, enzymes and other reagents**		
3’3-diaminobenzidine	Sigma-Aldrich	D12384
ABC Elite Kit	BIOZOL	VEC-PK-6100
Bafilomycin A1	InvivoGen	tlrl-baf1
B cell isolation kit, mouse	Miltenyi Biotec	130-090-862
BD Cytofix/Cytoperm solutionTM	BD	554714
BSA Albumin Bovine V	Serva	11903.03
Chloroquine	Sigma-Aldrich	C6628
DAPI	Sigma-Aldrich	D9542
DMEM high glucose	Life Technologies GmbH	31966047
DPBS	Sigma-Aldrich	D8537
Duolink In Situ Mounting Medium with DAPI	Merck	DEU82040
Duolink PLA Kit	Sigma-Aldrich	DUO92008
Durcupan resin	Sigma-Aldrich	44611
Dynabeads protein G	Thermo Fisher Scientific	10004D
EBSS	Thermo Fisher	14155063
EDTA-free protease inhibitor cocktail	Roche	5,893E + 09
Ethanol	VWR	20.821.310
Ethylenediaminetetraacetic acid solution (EDTA)	Sigma-Aldrich	E8008
Fetal bovine serum (FBS)	Thermo Fisher	F7524
Glutraldehyde	Sigma-Aldrich	354400
Goat serum	Sigma-Aldrich	G9023
HEPES	Sigma-Aldrich	H0887
Hydrochloric acid (HCl)	AppliChem	182883.12
IFN-γ ELISA Kit	MABTECH	3420-1H-6
IL-6 ELISA Kit	MABTECH	3460-1HP-1
Lead(II) acetate trihydrate	Merck Millipore	107375
Lipofectamine™ 2000	Invitrogen	11668-027
LPS from *E. coli* 055	Sigma-Aldrich	L2654
Lysotracker Red	Invitrogen	L12492
Methanol	Honeywell	32213
MG132	InvivoGen	tlrl-mg132-2
Milk powder	Roth	T145.1/.3
MIP-1β ELISA Kit	Thermo Fisher	88-7034-22
NP-40	Calbiochem	492018
Opti-MEM™ - Reduced Serum Medium	Thermo Fisher	31985070
Optiprep	Sigma-Aldrich	D1556
Osimum tetroxide (OsO4)	Sigma-Aldrich	201030
Paraformaldehyde	Sigma-Aldrich	158127
Penicillin-Streptomycin (10 000U/ml)	Thermo Fisher	15140122
Perm/Wash BufferTM	BD	554723
PI(3)P Mass ELISA kit	Echelon	K-3300
Pierce^TM^ BCA Protein Assay Kit	Thermo Fisher	23225
Potassium hydroxide (KOH)	Sigma-Aldrich	6103
Propylene oxide	Sigma-Aldrich	75-56-9
Proteinase K	Zymo Research	D3001-2
PVDF membrane	Bio-Rad Laboratories GmbH	1620177
Rapamycin	InvivoGen	tlrl-rap
Recombinant human IFN-γ	Peprotech	300-02
RPMI 1640 + L-Glutamine	Sigma-Aldrich	21875091
Saponin	Sigma-Aldrich	47036
Signal Fire/Plus chemiluminescent substrates	Cell Signaling	12630S
Silver enhancement HQ-Silver kit	Nanoprobe	2012
Sodium chloride (NaCl)	Roth	9265.1
Sodium deoxycholate	Sigma-Aldrich	89904
Sodium dodecyl sulfate (SDS)	Sigma-Aldrich	436143
Sodium pyruvate	Sigma-Aldrich	P5280
Sucrose	Sigma-Aldrich	84097
Sulfuric acid (H_2_SO_4_)	Sigma-Aldrich	339741
TMB substrate	MABTECH	3652-F10
TNF-α ELISA Kit	MABTECH	3512-1HP-1
Torin1	InVivoGen	inh-tor1
Trichloroacetic acid (TCA)	Sigma-Aldrich	8.223.420.250
Tris	Applichem GmbH	A1379
Triton X-100	Sigma-Aldrich	93443
Trypsin-EDTA (0.05%), phenol red	Thermo Fischer	25300-054
Tween 20	Sigma-Aldrich	P9416
Uranyl acetate	Polysciences	21447-25
Viability Dye eFluor780	Invitrogen	65-0865-14
VPS34-In1	Selleckchem	S7980
Wortmannin	InVivogen	tlrl-wtm
**Software**		
Affinity designer 2	Serif	N/A
Duolink Image Tool	Sigma-Aldrich	
FlowJo	Treestar Inc	
FUNCBase database		
GraphPad Prism 10		
High Ambiguity Driven Biomolecular DOCKing, version 2.2		
Homology modelling software Prime	Schrödinger Suite 2018-1, LLC	
ImageJ/FiJi		
ImageSP		
Imaris9.7	Oxford Instrument	
STRING database		
Zeiss blue/black	Zeiss	
**Other**		
BD FACS canto II	Beckton Dickinson	
Dounce homogenizer	WHEATON® Dounce Tissue Grinder	
Fusion SL device	Peqlab	
SP8	Leica	
Zeiss LSM 710	Zeiss	
Zeiss LSM 880	Zeiss	
Zeiss LEO 906 transmission electron microscope	Zeiss	

### Study protocol

Collection of peripheral blood mononuclear cells from healthy donors (HD) and LRBA-deficient patients to generate LCL cell lines was approved by the ethics committee of the University of Freiburg, Germany, vote n° 290/13. Patients have signed an informed consent, and experiments were performed conformed to the principles set out in the WMA Declaration of Helsinki and the Department of Health and Human Services Belmont report.

### Generation of a LRBA-KO HEK293T cell line, a HaCat and a Ramos cell line by CRISPR-Cas9, and a LRBA knock-down HeLa cell line by shRNA (shLRBA cells)

**Figure EV7:**
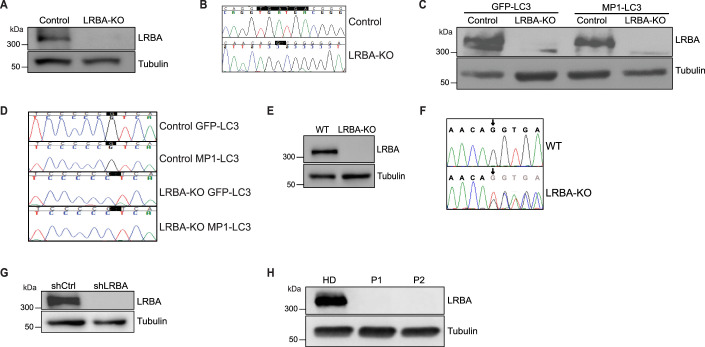
Generation of LRBA-deficient cell lines. (**A**, **C**, **E**) Immunoblot analyses of LRBA protein expression in WT and LRBA-KO (**A**) HEK293T, (**C**) HaCaT and (**E**) Ramos cells. (**B**, **D**, **F**) Sequencing analyses of *LRBA* exon 2 cells showing successful depletion of LRBA using the CRISPR-Cas9 system in (**B**) HEK293T, (**D**) HaCaT and (**F**) Ramos cells. (**G**) Immunoblot analyses of LRBA protein expression in HeLa cells shcontrol and shLRBA HeLa cells. (**H**) Immunoblot analyses of LRBA protein expression in LCL cells from a HD and two LRBA-deficient patients.

LRBA-KO HEK293T, HaCat and Ramos cells were generated using the Alt-R-CRISPR-Cas9 system according to the manufacturer´s instructions (www.idtdna.com). The gRNA CCACCAACAGGTGATGACGG specific for exon 2 of human LRBA, was inserted into the cells by electroporation together with the crRNA: tracrRNA complex and the Cas9. Following 48 h incubation, cells were single cell sorted by flow cytometry. LRBA-KO clones were validated by western blot for LRBA expression abolishment and by Sanger sequencing for mutation identification (Fig. EV7A–F). shLRBA HeLa cells were generated by lentiviral transfection with the vector pLKO.1 (SIGMA) containing the shRNA sequence: CCGGGCAGAAGATATTCACAGACATCTCGAGATGTCTGTGAATATCTT-CTGCTTTTTTG (TRCN0000148136, SIGMA) according to the manufacturer’s instructions (Fig. EV7G). Ramos cells were authenticated by STR profiling and all cells lines were tested weekly for Mycoplasma contamination.

### Human lymphoblastoid B cell line generation

LCL cells were generated from B cells from three HD and two LRBA-deficient patients (P1: c.2004+2 A > G; P2: p.S2713Hfs) after isolation from PBMCs by negative selection, and incubation 1:1 cell: EBV containing-media for four days. LCL actively proliferate, secrete antibodies and express LRBA (Fig. EV7H).

### Mice

*Lrba* knock-out mice (*Lrba*^*−/−*^) were kindly provided by Prof. Manfred Kilimann, Max-Planck-Institut für Experimentelle Medizin (MPIEM), Göttingen, Germany. They were generated on a C57BL/6N background by homologous recombination producing a loss-of-protein deletion of exon 4 of *Lrba* (C57BL/6N-Lrbatm1.1Kili/Ieg RRID:IMSR_EM:14924) (Kurtenbach et al, 2017). Mice were bred and maintained on a C57BL/6N background under specific pathogen-free (SPF) conditions at the animal facility of the Center for Experimental Models and Transgenic Service (CEMT), University Medical Center Freiburg, Germany. All animal experiments were approved by the local animal ethics committee (Regierungspräsidium Freiburg, Germany) under the following reference numbers: X-15/05F, G-16/19, G-16/94 and G15-168.

### Plasmids

Human LRBA was cloned in seven different fragments into pCIneo FLAG vectors (Promega). These plasmids were kindly provided by Dr. Bernice Lo from the National Institute of Health, Bethesda, USA (Lo et al, 2015). mCherry-GFP-LC3 plasmid was a kind gift from Dr. Ian Gentle from the University of Freiburg, Germany, whereas GFP-FYCO1 was kindly provided by Dr. Christian Behrends from the University of Frankfurt, Germany. Human LRBA full-length tagged to Myc-DDK was purchased in Origene. pVitro-hygro-N-myc-hVps34/Vps15-C-V5-his-plasmid (Addgene), pEGFP-Atg14L (Addgene) and pEGFP-C1-hUVRAG (Addgene) were provided by Jonathan Backer (Yan et al, 2009), Tamotsu Yoshimiri (Matsunaga et al, 2009) and Noburo Mizushima (Itakura et al, 2008), respectively. GFP-LC3 and MP1-LC3 plasmids were previously generated in the laboratory of Prof. Christian Münz from the University of Zürich, Switzerland.

### Reconstitution of LRBA

Rescue experiments were conducted in LRBA-KO HEK293T and HaCat cell lines transfected with 2 μg of WT LRBA plasmid tagged with Myc (Origen) using Lipofectamine 2000 (Invitrogen). These reconstituted cells lines are called Myc-LRBA in this manuscript. Details of the transfection protocol are described in the “Transfections and Immunoprecipitations” section below.

### Proximity ligation assay

In situ PLA was performed using Duolink kit (Sigma-Aldrich) in LCL cells from HD 1 and 2, and Patient 1 and 2. Cell fixation and permeabilization was performed with 4% PFA and 0.1% Triton X-100, respectively. Incubation with primary antibodies was followed by incubation with secondary antibodies that are conjugated with oligonucleotides, PLA probe anti-mouse (or anti-goat) MINUS and PLA probe anti-rabbit PLUS (Sigma-Aldrich). After ligation with DNA oligonucleotides and amplification with a DNA polymerase, the amplified product was detected as a fluorescent signal with a confocal microscope (Zeiss LSM700). Signal quantification was performed using Duolink Image Tool (Sigma-Aldrich).

### Transfections

HEK293T cells were co-transfected with 2 μg of one of the seven Flag-tagged-LRBA-fragment plasmids or Myc-LRBA plasmid, plus 2 μg of Myc-PIK3R4/His-VPS34, GFP-FYCO1, GFP-ATG14L or GFP-UVRAG using Lipofectamine 2000 (Invitrogen) according to the manufacturer´s protocol. Cells were harvested after 48 h and lysed with IP buffer (50 mM Tris-HCl pH 6.8, 150 mM NaCl, 0.2% NP-40, 1 mM EDTA), plus 1× complete EDTA-free protease inhibitor cocktail (Roche). Lysates were collected and used for immunoprecipitation experiments.

### Immunoprecipitations

LRBA IP, or flag-tagged LRBA IP or GFP IP were performed using 200 µg of cell lysates from HEK293T cells and 1 µg of either rabbit anti-LRBA (Sigma-Aldrich), or mouse anti-FLAG antibody (Sigma-Aldrich), mouse anti-PIK3R4 (Novus) or mouse anti-GFP (Santa Cruz). Then, 40 µl of Dynabeads protein G (Thermo Fischer Scientific) were added to the lysate/antibody mix and incubated overnight. Beads were washed with lysis buffer, and proteins were eluted with 2% SDS and resuspended in Laemmli buffer. In total, 20 µl of the eluted proteins were separated by SDS-PAGE, blotted and detected by immunoblotting.

### Immunoblotting and antibodies

Cell lysates were generated with RIPA buffer (50 mM Tris, 1% NP-40, 0.5% sodium deoxycholate, 100 mM NaCl, 1 mM EDTA, 0.1% SDS) + 1× complete EDTA-free protease inhibitor cocktail (Roche). Total protein concentrations were determined by bicinchoninic acid Protein Assay (Thermo Fisher Scientific). Protein lysates were size-fractionated by SDS-PAGE and electro transferred to a PVDF membrane in a wet blotting system for 1.5 h at 45 V. After blocking with 5% milk in TBST (20 mM Tris, 150 mM NaCl, 0.1% Tween 20), the membranes were incubated at 4 °C with any of the following primary antibodies: anti-AMPK total (Cell Signaling), anti-AMPK (pT172; Cell Signaling), anti-DFCP1 (LS-bio), anti-FLAG (Sigma-Aldrich), anti-FYCO1 (Proteintech), anti-GFP (Santa Cruz), anti-LC3 (Novus), anti-LRBA (Sigma-Aldrich), anti-Myc (Cell Signaling), anti-PIK3R4 (Novus), anti-p62 (Cell Signaling), anti-S6K total (Cell Signaling), anti-S6K70 (pT389; Cell Signaling), anti-P6RP (Cell Signalling), anti-VPS34 (Novusbio), anti-WIPI2 (Abcam). After overnight incubation with the primary antibodies, membranes were immunodetected with their corresponding secondary HRP-coupled antibody (Santa Cruz). Membranes were washed and developed with Signal Fire or Signal Fire Plus chemiluminescent substrates (Cell Signaling). HRP-linked anti-Tubulin (Proteintech) and anti-GAPDH (Cell Signaling) were used as a loading control. Peroxidase activity was detected with the Fusion SL device (Peqlab).

### Autophagy induction

Naive B cells (CD43^-^B220^+^CD3^-^) were obtained by negative selection (mouse CD43 Ly-48 MicroBeads; MACS Miltenyi Biotec) from spleens of WT and *Lrba*^*−/*−^ mice, followed by stimulation with LPS from *Escherichia coli* 055: B5 (20 µg/ml, Sigma-Aldrich) for 3 days in complete RPMI medium (L-glutamine, 10% FCS, 100 μg/mL streptomycin, 100 U/mL penicillin, 10 mM HEPES and 1 mM sodium pyruvate) with or without 100 nM of Bafilomycin A1 (InvivoGen) for 3 h. Next, cell pellets were either used for LC3-II detection by immunoblotting or for morphology and LRBA cellular localization analyses by electron microscopy. shControl and shLRBA HeLa cells were seeded at 0.3*10^6^ cells in six-well plates followed by treatment for 16 h or 24 h with 5 μM of MG132 (InvivoGen) alone or in combination with 100 nM of Bafilomycin A1 for 3 h.

### Measurement of phosphatidylinositol-3 phosphate (PI(3)P)

WT and LRBA-KO HEK293T cells were seeded and treated with 333 nM of Torin 1 (Invivogen) or with 1 nM of VPS34-IN (Biomol) for 4 h. After cell collection, cells lysis and extraction of neutral and acidic lipids, PI(3)P was obtained from the organic phase as described before (Chicanne et al, 2012). Finally, PI(3)P was measured using the PI(3)P Mass ELISA kit (Echelon) according to the manufacturer’s protocol.

### Fluorescence microscopy

To visualize autophagosomes, shWT and shLRBA HeLa cells were transfected with GFP-LC3 using Lipofectamine 2000 (Invitrogen). Lysosomes were visualized with LysoTracker Red (Invitrogen), according to the manufacturer’s instructions. To visualize autophagosomes and autolysosomes, WT and LRBA-KO HEK293T cells were either stably transduced with the mCherry-GFP-LC3 tandem vector (kindly provided by Dr. Ian Gentle, University of Freiburg), or stained endogenous LC3B. Cells were treated for 4 h with 333 nM Torin 1 (Invivogen) alone or in combination with 100 nM Bafilomycin A1 (Invivogen). To visualize endogenous expression of DFCP1 and WIPI2, WT and LRBA-KO HEK293T cells were treated for 1 h with 50 µM Rapamycin (Invivogen), 333 nM Torin 1 (Invivogen), 100 nM Wortmannin (Invivogen) or incubated in EBSS medium (Thermo Fisher) for starvation. After treatments, cells were fixed for 10 min with 4% PFA (Sigma-Aldrich) and permeabilized with either 0.05% Saponin (Sigma-Aldrich) or 0.1% Triton X-100. Endogenous LC3 expression was detected after 1 h incubation with anti-LC3B (Novus), followed by 45 min incubation with secondary antibodies anti-rabbit-AlexaFluor555 (Cell Signaling). DFCP-1 and WIPI2 expression was detected after overnight incubation with anti-DFCP1 (LS-bio) or anti-WIPI2 (Abcam) antibodies, followed by 1 h incubation with anti-mouse or anti-rabbit Alexa Fluor 488 or Alexa Fluor 647 antibodies (Cell Signaling). After staining, cover slides were mounted with DuoLink In Situ Mounting Medium with DAPI (Merck). The images were acquired using Zeiss LSM710 or LSM880 confocal microscope. The number of dots per cells and co-localization was assessed with ImageJ. The autophagosome size was assessed using Imaris 9.7 (Oxford Instruments). Autophagosomal size was determined from binary images using the analyses particle module of FIJI software (version X), with a particle size from 0 to 20 µm^2^. Autophagosomal mobility was evaluated by time-lapse movies of autophagosomes acquired in individual frames color-coded and projected into one image, using the temporal color-coding module of the Zeiss Zen Black software. Autophagosomes were followed for 25 frames and area and velocity were quantified in pixels.

### LC3B co-localization with HLA-DR molecules in MIICs experiments

HaCat cells were seeded in glass cover slips placed inside 24-well plate and stimulated overnight with IFN-ɣ to induce MHC-II expression. Cells were treated 6 h with 20 µM of chloroquine, and then with 4% PFA for 15 min at RT in the dark. Permeabilization was performed with 0.1% Triton X-100 for 5 min at RT, followed by three PBS washes. Then cells were saturated with blocking buffer (1% FBS-PBS) for 1 h at RT. Primary antibodies were diluted in the blocking buffer and incubated with the cells for 1 h at RT. Primary antibodies used: rabbit anti-LC3B (Novus), mouse anti-HLA-DR (Novus), mouse anti-HLA-DM AF488 (Santa Cruz) and mouse anti-p62 AF647 (Abcam). Three PBS washes were performed before staining with Alexa Fluor 488/555-conjugated goat anti-mouse or anti-rabbit (Thermo Fischer) for 1 h at RT. After three PBS washes, cell nuclei were stained with DAPI for 5 min prior to mounting the cover slip onto a glass slide using Duolink In Situ Mounting Medium with DAPI (Merck). Cells were visualized through 63×, 1.4 NA oil immersion objective with a confocal laser scanning microscope (LSM710, Zeiss) and images were processed with Fiji software.

### Membrane flotation assay

Cells from three 15-cm dishes were washed twice with PBS and harvested. The cell pellets were collected after centrifugation at 800 × *g* for 5 min and resuspended in homogenization buffer (250 mM sucrose, 20 mM HEPES-KOH pH 7.4, 1 mM EDTA and complete EDTA-free protease inhibitor cocktail (Roche). Cells were lysed by 40 strokes in a glass Dounce homogenizer (WHEATON® Dounce Tissue Grinder). Unbroken cells and debris were removed by two centrifugation steps at 2000 × *g* for 5 min. The supernatant was mixed with an equal volume of 50% OptiPrep (D1556-250ML; Sigma-Aldrich) in homogenization buffer. Discontinuous Optiprep gradients were prepared as described previously (Matsui et al, 2018), in SW41 tubes (344059; Beckman Coulter) by overlaying the following Optiprep solutions in homogenization buffer: 2.4 ml of the diluted sample (25% Optiprep), 1.8 ml of 20% Optiprep, 2 ml of 15% Optiprep, 2 ml of 10% Optiprep, 2 ml of 5% Optiprep and 2 ml of homogenization buffer without Optiprep. The gradients were spin and 13 fractions of 0.95 ml were collected from the top and subjected to TCA precipitation. The final pellet was resuspended in sample buffer and incubated at 95 °C for 5 min.

### Proteinase protection assay

Cells were treated for 2 h with EBSS and 300 nM Torin 1. Afterwards they were washed with PBS and collected by centrifugation at 500×*g* for 5 min. Pellets were resuspended in homogenization buffer (250 mM sucrose, 20 mM Hepes-KOH pH 7.4, 1 mM EDTA and complete EDTA-free protease inhibitor cocktail) and lysed by 30 passages with a 25 G needle. After two preclearing steps (2000 × *g*, 5 min), cell membranes were pelleted by centrifugation at 20,000 × *g* for 30 min. The pellet was resuspended in 100 µl of homogenization buffer without EDTA or protease inhibitors, divided into three equal fractions and incubated in the presence or absence of proteinase K (100 µg per ml of sample) with or without 0.5% Triton X-100 for 30 min on ice. The samples were then subjected to TCA precipitation and resuspended in sample buffer.

### Pre-embedding immunoperoxidase and immunogold electron microscopy (EM)

Following fixation in 0.05% glutaraldehyde and 4% PFA in 0.1 M phosphate buffer (PB), cell pellets were embedded in agar and cut onto 100 µm slices using a Vibratome. Slices were blocked with 20% normal goat serum and incubated with anti-Lamp1 (Invitrogen) or anti-LC3 (Cell Signalling) antibodies, followed by incubation with biotinylated anti-mouse and with goat anti-rabbit IgG coupled to 1.4 nm gold particles in 2% NGS/ 50 mM Tris-buffered saline. Slices were postfixed in 1% glutaraldehyde, and the gold particles were enlarged using the silver enhancement kit HQ-Silver from Nanoprobe. Slices were washed with ABC Elite Kit and peroxidase reaction was visualized by 3’3-diaminobenzidine. After washing, slices were postfixed with 0.5% OsO_4_/ 1% uranyl acetate, dehydrated in a graded series of ethanol, treated with propylene oxide and embedded in Durcupan resin. Slices were cut into 60 nm ultrathin sections, counterstained with lead citrate and viewed in a Zeiss LEO 906 transmission electron microscope. Images were taken using the sharp-eye 2k CCD camera and processed with ImageSP.

### Flow cytometry analyses

Intracellular expression of HLA-DR in IFN-γ-stimulated HaCat cells, intracellular expression of pS6k and pAMPK in LCL cells and intracellular expression of p62 in Ramos cells were determined as follows: 3 × 10^5^ cells were first resuspended in PBS and stained for fixable viability dye (Invitrogen) for 20 min at 4 °C protected from light. Next, cells were fixed and permeabilized for 20 min using BD Cytofix/Cytoperm solution^TM^ (BD), and then washed twice with 1× Perm/Wash Buffer^TM^ (BD). Subsequently, cells were resuspended in 1× Perm/Wash Buffer and stained with conjugated anti-pS6K-Pacific Blue (Cell Signaling) for LCL cells, or with conjugated anti-HLA-DR (Invitrogen) for HaCat cells for 1 h at 4 °C or with conjugated anti-p62 (Abcam) for Ramos cells for 30 min at 4 °C. After washing two times, a secondary antibody anti-rabbit IgG-FITC (Invitrogen) was added to LCL cells and incubated at 4 °C for 25 min. Cells were then washed and acquired on a FACS Canto II (BD). Data analyses and calculation of the geometric mean fluorescence intensity (MFI) were performed using FlowJoTM 7.6.5 software (TreeStar Inc., Ashland, OR, USA). Gating strategy excluded doublets according to their FSC-H and FSC-A and dead cells, which were positive for fixable viability dye.

### ELISA

WT and LRBA-KO HaCat cells stably expressing GFP-LC3 or MP1-LC3 (target cells), and pre-treated with IFN-γ for 24 h to up-regulate MHC-II molecules were cultured with a MP1-specific CD4+ T cell clone (effector cells) from a HD (E:T = 10:5) for 20 h. Levels of TNF-α, IL-6, IFN-γ and MIP-1β were measured by ELISA in the collected supernatant as follows, 96-well flat-bottom plates (Corning Costar) were coated with 100 μl per well of 2 μg/ml of capture unlabeled mouse anti-human TNF-α (MABTECH), IL-6 (MABTECH,), IFN-γ (MABTECH), and MIP-1β (Thermo Fisher). Following overnight incubation at 4 °C, plates were incubated with blocking buffer (PBS with 0.05% Tween and 0.1% BSA) for 1 h at RT. Next, corresponding cytokine standard or serial dilutions of culture supernatant was added to each well and incubated for 2 h at RT. Biotin-conjugated detection antibody specific for each cytokine (MABTECH and Thermo Fisher) were added, and incubated for 1 h at RT. Streptavidin-HRP solution diluted 1:1000 in PBS was added and incubated for 1 h followed by incubation with TMB substrate (MABTECH) for 15 min protected from direct light. After stopping the reaction with 0.2 M H2SO4 the optical density was detected in an ELISA reader at 450 nm within 15 min. Cytokine levels of supernatants were determined according to the standard curve.

### In silico analyses

The LRBA protein interaction candidates were obtained from STRING and FUNCBase databases that provide known and predicted physical and functional protein-protein interactions. Only interactions with high confidence levels (>0.7) were selected from both databases for further validation. Network visualization was performed with the Cytoscape software provided for both databases.

### Modelling of the complex of LRBA and PIK3R4/PIK3C3

The structural modelling of the complex of LRBA and PIK3R4 was based on the finding that LRBA interacts via the WD40 domain with PIK3R4 (see results section). The 3D structure of the WD40 domain was modelled by homology modelling applying the structure of the homolog prokaryotic protein PkwA from *Thermomonospora curvata* (Brohawn et al, 2008). Sequence identity was 26% (39% shared similar residues). Since the first repeat sequence of the WD40-propeller of human LRBA is separated from the other WD40-repeats in sequence, this part of the domain was modelled based on a template of a single propeller and merged with the others, forming a full WD40 propeller domain. For the merging, a WD40 domain with an inserted propeller was used as a structural template (Wang et al, 2015)). For the structural modelling of the complex of PIK3R4 with PIK3C3 the homologous structures of the Phosphatidylinositol 3-kinase VPS34 in complex with serine/threonine-protein kinase VPS15 from Saccharomyces cerevisiae was applied. Human PIK3C and the template share a sequence identity of 36% (with 54% similar residues). Human PIK3R4 and VPS15 share a sequence identity of 33% (50% similar residues). The modelling was performed with the homology modelling software Prime (Schrödinger Suite 2018-1, LLC). For the model of the WD40 domain of PIK3R4 we applied the modelling server WDSPdb 2.0 that accurately identifies WD40 repeats and includes them for the correct assignment of the beta sheets in the structural model (Wang et al, 2015). The WD40 domain was then connected with the kinase domain of PIK3R4 and finally energy minimized using the OPLS3 force field provided by the Schrödinger software package. The modelling of the complex of PIK3R4/PIK3C3 and LRBA was performed by applying the docking web server HADDOCK (High Ambiguity Driven Biomolecular DOCKing, version 2.2, (van Zundert et al, 2016). As input constraints, the single exposed ɑ-helix of the PIK3R4-WD40 domain (sequence motif: VGPSDD) and the smaller top surface of the WD40 domain of LRBA were defined as interacting regions. The top-ranked cluster with the highest negative Z-score value (-2.0) was selected for further analyses.

### Data and statistical analyses

Data analysis was performed without blinding and statistical significance was calculated with a non-parametric an unpaired Welch’s *t* test, ratio paired Student *t* test, a one-way ANOVA with Tukey’s multiple comparisons test or a two-way ANOVA with Bonferroni’s multiple comparisons test using GraphPad Prism 6.0 software. A *P* value of <0.05 was considered statistically significant (*P < 0.05; **P < 0.01; ***P < 0.001; ****P < 0.0001).

## Supplementary information

Peer Review File

Movie EV1

Movie EV2

Source data Fig. 1

Source data Fig. 2

Source data Fig. 3

Source data Fig. 4

Source data Fig. 5

Source data Fig. 6

Expanded View Figures

## Data Availability

This study includes no data deposited in external repositories. The source data of this paper are collected in the following database record: biostudies:S-SCDT-10_1038-S44319-025-00504-7.

## References

[CR1] Abada A, Levin-Zaidman S, Porat Z, Dadosh T, Elazar Z (2017) SNARE priming is essential for maturation of autophagosomes but not for their formation. Proc Natl Acad Sci USA 114:12749–12754 29138318 10.1073/pnas.1705572114 PMC5715740

[CR2] Abrahamsen H, Stenmark H, Platta HW (2012) Ubiquitination and phosphorylation of Beclin 1 and its binding partners: tuning class III phosphatidylinositol 3-kinase activity and tumor suppression. FEBS Lett 586:1584–1591 22673570 10.1016/j.febslet.2012.04.046

[CR3] Alkhairy OK, Abolhassani H, Rezaei N, Fang M, Andersen KK, Chavoshzadeh Z, Mohammadzadeh I, El-Rajab MA, Massaad M, Chou J et al (2016) Spectrum of phenotypes associated with mutations in LRBA. J Clin Immunol 36:33–45 26707784 10.1007/s10875-015-0224-7

[CR4] Arbogast F, Arnold J, Hammann P, Kuhn L, Chicher J, Murera D, Weishaar J, Muller S, Fauny JD, Gros F (2019) ATG5 is required for B cell polarization and presentation of particulate antigens. Autophagy 15:280–294 30196744 10.1080/15548627.2018.1516327 PMC6333460

[CR5] Axe EL, Walker SA, Manifava M, Chandra P, Roderick HL, Habermann A, Griffiths G, Ktistakis NT (2008) Autophagosome formation from membrane compartments enriched in phosphatidylinositol 3-phosphate and dynamically connected to the endoplasmic reticulum. J Cell Biol 182:685–701 18725538 10.1083/jcb.200803137 PMC2518708

[CR6] Azizi G, Abolhassani H, Mahdaviani SA, Chavoshzadeh Z, Eshghi P, Yazdani R, Kiaee F, Shaghaghi M, Mohammadi J, Rezaei N et al (2017a) Clinical, immunologic, molecular analyses and outcomes of Iranian patients with LRBA deficiency: a longitudinal study. Pediatr Allergy Immunol 28:478–484 28512785 10.1111/pai.12735

[CR7] Azizi G, Abolhassani H, Yazdani R, Mohammadikhajehdehi S, Parvaneh N, Negahdari B, Mohammadi J, Aghamohammadi A (2017b) New therapeutic approach by sirolimus for enteropathy treatment in patients with LRBA deficiency. Eur Ann Allergy Clin Immunol 49:235–239 28884992 10.23822/EurAnnACI.1764-1489.22

[CR8] Backer JM (2016) The intricate regulation and complex functions of the Class III phosphoinositide 3-kinase Vps34. Biochem J 473:2251–2271 27470591 10.1042/BCJ20160170

[CR9] Beaver JE, Tasan M, Gibbons FD, Tian W, Hughes TR, Roth FP (2010) FuncBase: a resource for quantitative gene function annotation. Bioinformatics 26:1806–1807 20495000 10.1093/bioinformatics/btq265 PMC2894510

[CR10] Behrends C, Sowa ME, Gygi SP, Harper JW (2010) Network organization of the human autophagy system. Nature 466:68–76 20562859 10.1038/nature09204 PMC2901998

[CR11] Bilanges B, Alliouachene S, Pearce W, Morelli D, Szabadkai G, Chung YL, Chicanne G, Valet C, Hill JM, Voshol PJ et al (2017) Vps34 PI 3-kinase inactivation enhances insulin sensitivity through reprogramming of mitochondrial metabolism. Nat Commun 8:1804 29180704 10.1038/s41467-017-01969-4 PMC5703854

[CR12] Bilanges B, Posor Y, Vanhaesebroeck B (2019) PI3K isoforms in cell signalling and vesicle trafficking. Nat Rev Mol Cell Biol 20:515–534 31110302 10.1038/s41580-019-0129-z

[CR13] Brachmann S, Fritsch C, Maira SM, Garcia-Echeverria C (2009) PI3K and mTOR inhibitors: a new generation of targeted anticancer agents. Curr Opin Cell Biol 21:194–198 19201591 10.1016/j.ceb.2008.12.011

[CR14] Brohawn SG, Leksa NC, Spear ED, Rajashankar KR, Schwartz TU (2008) Structural evidence for common ancestry of the nuclear pore complex and vesicle coats. Science 322:1369–1373 18974315 10.1126/science.1165886 PMC2680690

[CR15] Charbonnier LM, Janssen E, Chou J, Ohsumi TK, Keles S, Hsu JT, Massaad MJ, Garcia-Lloret M, Hanna-Wakim R, Dbaibo G et al (2015) Regulatory T-cell deficiency and immune dysregulation, polyendocrinopathy, enteropathy, X-linked-like disorder caused by loss-of-function mutations in LRBA. J Allergy Clin Immunol 135:217–227 25468195 10.1016/j.jaci.2014.10.019 PMC4289093

[CR16] Chen J, Ma Z, Jiao X, Fariss R, Kantorow WL, Kantorow M, Pras E, Frydman M, Pras E, Riazuddin S et al (2011) Mutations in FYCO1 cause autosomal-recessive congenital cataracts. Am J Hum Genet 88:827–838 21636066 10.1016/j.ajhg.2011.05.008 PMC3113247

[CR17] Chicanne G, Severin S, Boscheron C, Terrisse AD, Gratacap MP, Gaits-Iacovoni F, Tronchere H, Payrastre B (2012) A novel mass assay to quantify the bioactive lipid PtdIns3P in various biological samples. Biochem J 447:17–23 22830526 10.1042/BJ20120945 PMC3441130

[CR18] Crotzer VL, Blum JS (2009) Autophagy and its role in MHC-mediated antigen presentation. J Immunol 182:3335–3341 19265109 10.4049/jimmunol.0803458 PMC2730830

[CR19] Cui B, Lin H, Yu J, Yu J, Hu Z (2019) Autophagy and the immune response. Adv Exp Med Biol 1206:595–634 31777004 10.1007/978-981-15-0602-4_27 PMC7120363

[CR20] Cullinane AR, Schaffer AA, Huizing M (2013) The BEACH is hot: a LYST of emerging roles for BEACH-domain containing proteins in human disease. Traffic 14:749–766 23521701 10.1111/tra.12069 PMC3761935

[CR21] Dikic I, Elazar Z (2018) Mechanism and medical implications of mammalian autophagy. Nat Rev Mol Cell Biol 19:349–364 29618831 10.1038/s41580-018-0003-4

[CR22] Ding WX, Ni HM, Gao W, Yoshimori T, Stolz DB, Ron D, Yin XM (2007) Linking of autophagy to ubiquitin-proteasome system is important for the regulation of endoplasmic reticulum stress and cell viability. Am J Pathol 171:513–524 17620365 10.2353/ajpath.2007.070188 PMC1934546

[CR23] Dominguez C, Boelens R, Bonvin AM (2003) HADDOCK: a protein-protein docking approach based on biochemical or biophysical information. J Am Chem Soc 125:1731–1737 12580598 10.1021/ja026939x

[CR24] Dooley HC, Razi M, Polson HE, Girardin SE, Wilson MI, Tooze SA (2014) WIPI2 links LC3 conjugation with PI3P, autophagosome formation, and pathogen clearance by recruiting Atg12-5-16L1. Mol Cell 55:238–252 24954904 10.1016/j.molcel.2014.05.021 PMC4104028

[CR25] Egan D, Kim J, Shaw RJ, Guan KL (2011) The autophagy initiating kinase ULK1 is regulated via opposing phosphorylation by AMPK and mTOR. Autophagy 7:643–644 21460621 10.4161/auto.7.6.15123 PMC3359466

[CR26] Falasca M, Maffucci T (2009) Rethinking phosphatidylinositol 3-monophosphate. Biochim Biophys Acta 1793:1795–1803 19852987 10.1016/j.bbamcr.2009.10.003

[CR27] Funderburk SF, Wang QJ, Yue Z (2010) The Beclin 1-VPS34 complex-at the crossroads of autophagy and beyond. Trends Cell Biol 20:355–362 20356743 10.1016/j.tcb.2010.03.002 PMC3781210

[CR28] Gamez-Diaz L, August D, Stepensky P, Revel-Vilk S, Seidel MG, Noriko M, Morio T, Worth AJJ, Blessing J, Van de Veerdonk F et al (2016) The extended phenotype of LPS-responsive beige-like anchor protein (LRBA) deficiency. J Allergy Clin Immunol 137:223–230 26768763 10.1016/j.jaci.2015.09.025

[CR29] Gaullier JM, Ronning E, Gillooly DJ, Stenmark H (2000) Interaction of the EEA1 FYVE finger with phosphatidylinositol 3-phosphate and early endosomes: role of conserved residues. J Biol Chem 275:24595–24600 10807926 10.1074/jbc.M906554199

[CR30] Goodman M, Liu Z, Zhu P, Li J (2014) AMPK activators as a drug for diabetes, cancer and cardiovascular disease. Pharm Regul Aff 3:118 27478687 10.4172/2167-7689.1000118 PMC4966671

[CR31] Hampe J, Franke A, Rosenstiel P, Till A, Teuber M, Huse K, Albrecht M, Mayr G, De La Vega FM, Briggs J et al (2007) A genome-wide association scan of nonsynonymous SNPs identifies a susceptibility variant for Crohn disease in ATG16L1. Nat Genet 39:207–211 17200669 10.1038/ng1954

[CR32] He C, Wang S, Zhou C, He M, Wang J, Ladds M, Lianoudaki D, Sedimbi SK, Lane DP, Westerberg LS et al (2021) CD36 and LC3B initiated autophagy in B cells regulates the humoral immune response. Autophagy 17:3577–3591 33535890 10.1080/15548627.2021.1885183 PMC8632284

[CR33] Isakson P, Holland P, Simonsen A (2013) The role of ALFY in selective autophagy. Cell Death Differ 20:12–20 22653340 10.1038/cdd.2012.66 PMC3524637

[CR34] Itakura E, Kishi C, Inoue K, Mizushima N (2008) Beclin 1 forms two distinct phosphatidylinositol 3-kinase complexes with mammalian Atg14 and UVRAG. Mol Biol Cell 19:5360–5372 18843052 10.1091/mbc.E08-01-0080 PMC2592660

[CR35] Jaber N, Dou Z, Chen JS, Catanzaro J, Jiang YP, Ballou LM, Selinger E, Ouyang X, Lin RZ, Zhang J, Zong WX (2012) Class III PI3K Vps34 plays an essential role in autophagy and in heart and liver function. Proc Natl Acad Sci USA 109:2003–2008 22308354 10.1073/pnas.1112848109 PMC3277541

[CR36] Jain BP, Pandey S (2018) WD40 repeat proteins: signalling scaffold with diverse functions. Protein J 37:391–406 30069656 10.1007/s10930-018-9785-7

[CR37] Jamee M, Hosseinzadeh S, Sharifinejad N, Zaki-Dizaji M, Matloubi M, Hasani M, Baris S, Alsabbagh M, Lo B, Azizi G (2021) Comprehensive comparison between 222 CTLA-4 haploinsufficiency and 212 LRBA deficiency patients: a systematic review. Clin Exp Immunol 205:28–43 33788257 10.1111/cei.13600 PMC8209572

[CR38] Janman D, Hinze C, Kennedy A, Halliday N, Waters E, Williams C, Rowshanravan B, Hou TZ, Minogue S, Qureshi OS, Sansom DM (2021) Regulation of CTLA-4 recycling by LRBA and Rab11. Immunology 164:106–119 33960403 10.1111/imm.13343 PMC8358724

[CR39] Johnson EE, Overmeyer JH, Gunning WT, Maltese WA (2006) Gene silencing reveals a specific function of hVps34 phosphatidylinositol 3-kinase in late versus early endosomes. J Cell Sci 119:1219–1232 16522686 10.1242/jcs.02833

[CR40] Kim YC, Guan KL (2015) mTOR: a pharmacologic target for autophagy regulation. J Clin Invest 125:25–32 25654547 10.1172/JCI73939 PMC4382265

[CR41] Kostel Bal S, Haskologlu S, Serwas NK, Islamoglu C, Aytekin C, Kendirli T, Kuloglu Z, Yavuz G, Dalgic B, Siklar Z et al (2017) Multiple presentations of LRBA deficiency: a single-center experience. J Clin Immunol 37:790–800 28956255 10.1007/s10875-017-0446-y PMC7086713

[CR42] Kurtenbach S, Giessl A, Stromberg S, Kremers J, Atorf J, Rasche S, Neuhaus EM, Herve D, Brandstatter JH, Asan E et al (2017) The BEACH protein LRBA promotes the localization of the heterotrimeric G-protein GOLF to olfactory cilia. Sci Rep 7:8409 28814779 10.1038/s41598-017-08543-4 PMC5559528

[CR43] Lagos J, Sagadiev S, Diaz J, Bozo JP, Guzman F, Stefani C, Zanlungo S, Acharya M, Yuseff MI (2022) Autophagy induced by Toll-like receptor ligands regulates antigen extraction and presentation by B cells. Cells 11:3883 36497137 10.3390/cells11233883 PMC9741325

[CR44] Liang C, Lee JS, Inn KS, Gack MU, Li Q, Roberts EA, Vergne I, Deretic V, Feng P, Akazawa C, Jung JU (2008) Beclin1-binding UVRAG targets the class C Vps complex to coordinate autophagosome maturation and endocytic trafficking. Nat Cell Biol 10:776–787 18552835 10.1038/ncb1740 PMC2878716

[CR45] Ljunggren G, Anderson DJ (1998) Cytokine induced modulation of MHC class I and class II molecules on human cervical epithelial cells. J Reprod Immunol 38:123–138 9730287 10.1016/s0165-0378(98)00009-6

[CR46] Lo B, Fritz JM, Su HC, Uzel G, Jordan MB, Lenardo MJ (2016) CHAI and LATAIE: new genetic diseases of CTLA-4 checkpoint insufficiency. Blood 128:1037–1042 27418640 10.1182/blood-2016-04-712612 PMC5000841

[CR47] Lo B, Zhang K, Lu W, Zheng L, Zhang Q, Kanellopoulou C, Zhang Y, Liu Z, Fritz JM, Marsh R et al (2015) AUTOIMMUNE DISEASE. Patients with LRBA deficiency show CTLA4 loss and immune dysregulation responsive to abatacept therapy. Science 349:436–440 26206937 10.1126/science.aaa1663

[CR48] Lopez A, Fleming A, Rubinsztein DC (2018) Seeing is believing: methods to monitor vertebrate autophagy in vivo. Open Biol 8:180106 30355753 10.1098/rsob.180106 PMC6223212

[CR49] Lopez-Herrera G, Tampella G, Pan-Hammarstrom Q, Herholz P, Trujillo-Vargas CM, Phadwal K, Simon AK, Moutschen M, Etzioni A, Mory A et al (2012) Deleterious mutations in LRBA are associated with a syndrome of immune deficiency and autoimmunity. Am J Hum Genet 90:986–1001 22608502 10.1016/j.ajhg.2012.04.015 PMC3370280

[CR50] Mackeh R, Perdiz D, Lorin S, Codogno P, Pous C (2013) Autophagy and microtubules—new story, old players. J Cell Sci 126:1071–1080 23620510 10.1242/jcs.115626

[CR51] Marat AL, Haucke V (2016) Phosphatidylinositol 3-phosphates-at the interface between cell signalling and membrane traffic. EMBO J 35:561–579 26888746 10.15252/embj.201593564 PMC4801949

[CR52] Martinez Jaramillo C, Trujillo-Vargas CM (2018) LRBA in the endomembrane system. Colomb Med 49:236–243 10.25100/cm.v49i2.3802 PMC6220489 30410199

[CR53] Matsui T, Jiang P, Nakano S, Sakamaki Y, Yamamoto H, Mizushima N (2018) Autophagosomal YKT6 is required for fusion with lysosomes independently of syntaxin 17. J Cell Biol 217:2633–2645 29789439 10.1083/jcb.201712058 PMC6080929

[CR54] Matsunaga K, Saitoh T, Tabata K, Omori H, Satoh T, Kurotori N, Maejima I, Shirahama-Noda K, Ichimura T, Isobe T et al (2009) Two Beclin 1-binding proteins, Atg14L and Rubicon, reciprocally regulate autophagy at different stages. Nat Cell Biol 11:385–396 19270696 10.1038/ncb1846

[CR55] McKnight NC, Zhong Y, Wold MS, Gong S, Phillips GR, Dou Z, Zhao Y, Heintz N, Zong WX, Yue Z (2014) Beclin 1 is required for neuron viability and regulates endosome pathways via the UVRAG-VPS34 complex. PLoS Genet 10:e1004626 25275521 10.1371/journal.pgen.1004626 PMC4183436

[CR56] McLeod IX, Saxena R, Carico Z, He YW (2021) Class I PI3K provide lipid substrate in T cell autophagy through linked activity of inositol phosphatases. Front Cell Dev Biol 9:709398 34458267 10.3389/fcell.2021.709398 PMC8397451

[CR57] Mizushima N (2007) Autophagy: process and function. Genes Dev 21:2861–2873 18006683 10.1101/gad.1599207

[CR58] Mrakovic A, Kay JG, Furuya W, Brumell JH, Botelho RJ (2012) Rab7 and Arl8 GTPases are necessary for lysosome tubulation in macrophages. Traffic 13:1667–1679 22909026 10.1111/tra.12003

[CR59] Munz C (2016) Autophagy beyond intracellular MHC class II antigen presentation. Trends Immunol 37:755–763 27667710 10.1016/j.it.2016.08.017

[CR60] Munz C (2021) The macroautophagy machinery in MHC restricted antigen presentation. Front Immunol 12:628429 33717153 10.3389/fimmu.2021.628429 PMC7947692

[CR61] Nahse V, Raiborg C, Tan KW, Mork S, Torgersen ML, Wenzel EM, Nager M, Salo VT, Johansen T, Ikonen E et al (2023) ATPase activity of DFCP1 controls selective autophagy. Nat Commun 14:4051 37422481 10.1038/s41467-023-39641-9 PMC10329651

[CR62] Nascimbeni AC, Codogno P, Morel E (2017) Phosphatidylinositol-3-phosphate in the regulation of autophagy membrane dynamics. FEBS J 284:1267–1278 27973739 10.1111/febs.13987

[CR85] Nguyen T, Padman B, Zellner S, Khuu G, Uoselis L, Lam W, Skulsuppaisarn M, Lindblom Watts E, Behrends C, Lazarou M (2021). ATG4 family proteins drive phagophore growth independently of the LC3/GABARAP lipidation system. Mol. Cell. 81:2013–2030.e9. 10.1016/j.molcel.2021.03.001 33773106

[CR63] Nieto-Torres JL, Shanahan SL, Chassefeyre R, Chaiamarit T, Zaretski S, Landeras-Bueno S, Verhelle A, Encalada SE, Hansen M (2021) LC3B phosphorylation regulates FYCO1 binding and directional transport of autophagosomes. Curr Biol 31:3440–3449.e3447 34146484 10.1016/j.cub.2021.05.052 PMC8439105

[CR64] Nunes-Santos CJ, Uzel G, Rosenzweig SD (2019) PI3K pathway defects leading to immunodeficiency and immune dysregulation. J Allergy Clin Immunol 143:1676–1687 31060715 10.1016/j.jaci.2019.03.017

[CR65] Ohashi Y, Tremel S, Williams RL (2019) VPS34 complexes from a structural perspective. J Lipid Res 60:229–241 30397185 10.1194/jlr.R089490 PMC6358306

[CR66] Palamiuc L, Ravi A, Emerling BM (2020) Phosphoinositides in autophagy: current roles and future insights. FEBS J 287:222–238 31693781 10.1111/febs.15127 PMC9154050

[CR67] Pankiv S, Alemu EA, Brech A, Bruun JA, Lamark T, Overvatn A, Bjorkoy G, Johansen T (2010) FYCO1 is a Rab7 effector that binds to LC3 and PI3P to mediate microtubule plus end-directed vesicle transport. J Cell Biol 188:253–269 20100911 10.1083/jcb.200907015 PMC2812517

[CR68] Pankiv S, Johansen T (2010) FYCO1: linking autophagosomes to microtubule plus end-directing molecular motors. Autophagy 6:550–552 20364109 10.4161/auto.6.4.11670

[CR69] Pengo N, Cenci S (2013) The role of autophagy in plasma cell ontogenesis. Autophagy 9:942–944 23528926 10.4161/auto.24399 PMC3672309

[CR70] Pengo N, Scolari M, Oliva L, Milan E, Mainoldi F, Raimondi A, Fagioli C, Merlini A, Mariani E, Pasqualetto E et al (2013) Plasma cells require autophagy for sustainable immunoglobulin production. Nat Immunol 14:298–305 23354484 10.1038/ni.2524

[CR71] Polson HE, de Lartigue J, Rigden DJ, Reedijk M, Urbe S, Clague MJ, Tooze SA (2010) Mammalian Atg18 (WIPI2) localizes to omegasome-anchored phagophores and positively regulates LC3 lipidation. Autophagy 6:506–522 20505359 10.4161/auto.6.4.11863

[CR72] Pu J, Guardia CM, Keren-Kaplan T, Bonifacino JS (2016) Mechanisms and functions of lysosome positioning. J Cell Sci 129:4329–4339 27799357 10.1242/jcs.196287 PMC5201012

[CR73] Runsala M, Kuokkanen E, Uski E, Sustar V, Balci MO, Rajala J, Paavola V, Mattila PK (2023) The small GTPase Rab7 regulates antigen processing in B cells in a possible interplay with autophagy machinery. Cells 12:2566 37947644 10.3390/cells12212566 PMC10649364

[CR74] Sarbassov DD, Ali SM, Kim DH, Guertin DA, Latek RR, Erdjument-Bromage H, Tempst P, Sabatini DM (2004) Rictor, a novel binding partner of mTOR, defines a rapamycin-insensitive and raptor-independent pathway that regulates the cytoskeleton. Curr Biol 14:1296–1302 15268862 10.1016/j.cub.2004.06.054

[CR75] Schmid D, Pypaert M, Munz C (2007) Antigen-loading compartments for major histocompatibility complex class II molecules continuously receive input from autophagosomes. Immunity 26:79–92 17182262 10.1016/j.immuni.2006.10.018 PMC1805710

[CR76] Seidel MG, Bohm K, Dogu F, Worth A, Thrasher A, Florkin B, Ikinciogullari A, Peters A, Bakhtiar S, Meeths M et al (2018) Treatment of severe forms of LPS-responsive beige-like anchor protein deficiency with allogeneic hematopoietic stem cell transplantation. J Allergy Clin Immunol 141:770–775.e771 28502825 10.1016/j.jaci.2017.04.023

[CR77] Shao BZ, Yao Y, Zhai JS, Zhu JH, Li JP, Wu K (2021) The role of autophagy in inflammatory bowel disease. Front Physiol 12:621132 33633585 10.3389/fphys.2021.621132 PMC7902040

[CR78] Szklarczyk D, Morris JH, Cook H, Kuhn M, Wyder S, Simonovic M, Santos A, Doncheva NT, Roth A, Bork P et al (2017) The STRING database in 2017: quality-controlled protein-protein association networks, made broadly accessible. Nucleic Acids Res 45:D362–D368 27924014 10.1093/nar/gkw937 PMC5210637

[CR79] Taghizade N, Babayeva R, Kara A, Karakus IS, Catak MC, Bulutoglu A, Haskologlu ZS, Akay Haci I, Tunakan Dalgic C, Karabiber E et al (2023) Therapeutic modalities and clinical outcomes in a large cohort with LRBA deficiency and CTLA4 insufficiency. J Allergy Clin Immunol 152(6):1634–1645 37595759 10.1016/j.jaci.2023.08.004

[CR80] van Zundert GCP, Rodrigues J, Trellet M, Schmitz C, Kastritis PL, Karaca E, Melquiond ASJ, van Dijk M, de Vries SJ, Bonvin A (2016) The HADDOCK2.2 web server: user-friendly integrative modeling of biomolecular complexes. J Mol Biol 428:720–725 26410586 10.1016/j.jmb.2015.09.014

[CR81] Wang KW, Zhan X, McAlpine W, Zhang Z, Choi JH, Shi H, Misawa T, Yue T, Zhang D, Wang Y et al (2019) Enhanced susceptibility to chemically induced colitis caused by excessive endosomal TLR signaling in LRBA-deficient mice. Proc Natl Acad Sci USA 116:11380–11389 31097594 10.1073/pnas.1901407116 PMC6561264

[CR82] Wang Y, Hu XJ, Zou XD, Wu XH, Ye ZQ, Wu YD (2015) WDSPdb: a database for WD40-repeat proteins. Nucleic Acids Res 43:D339–344 25348404 10.1093/nar/gku1023 PMC4383882

[CR83] Willinger T, Flavell RA (2012) Canonical autophagy dependent on the class III phosphoinositide-3 kinase Vps34 is required for naive T-cell homeostasis. Proc Natl Acad Sci USA 109:8670–8675 22592798 10.1073/pnas.1205305109 PMC3365213

[CR84] Yan Y, Flinn RJ, Wu H, Schnur RS, Backer JM (2009) hVps15, but not Ca2+/CaM, is required for the activity and regulation of hVps34 in mammalian cells. Biochem J 417:747–755 18957027 10.1042/BJ20081865 PMC2652830

