# Supplementary figures and images for: LRBA deficiency impairs autophagy and contributes to enhanced antigen presentation and T-cell dysregulation
Source: EMBO Rep. 2025 Jun 23;26(16):4040–71. doi: 10.1038/s44319-025-00504-7 (PMC12373796; doi:10.1038/s44319-025-00504-7)

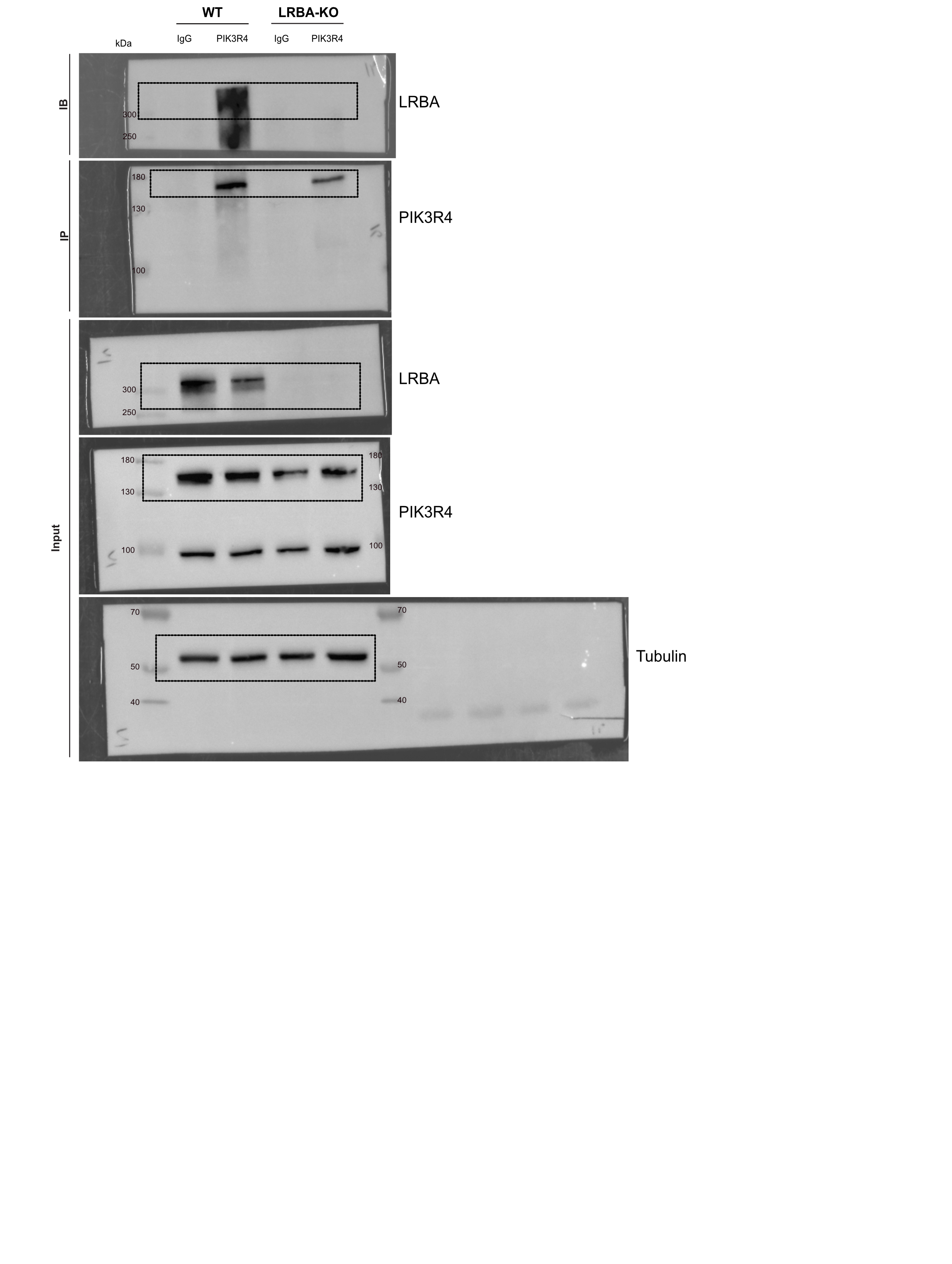

Supplement: Supplementary file 4 — Source data Fig. 1 [file 44319_2025_504_MOESM4_ESM.zip › Figure 1B/Endogenous Co-IP LRBA and PIK3R4.tiff]

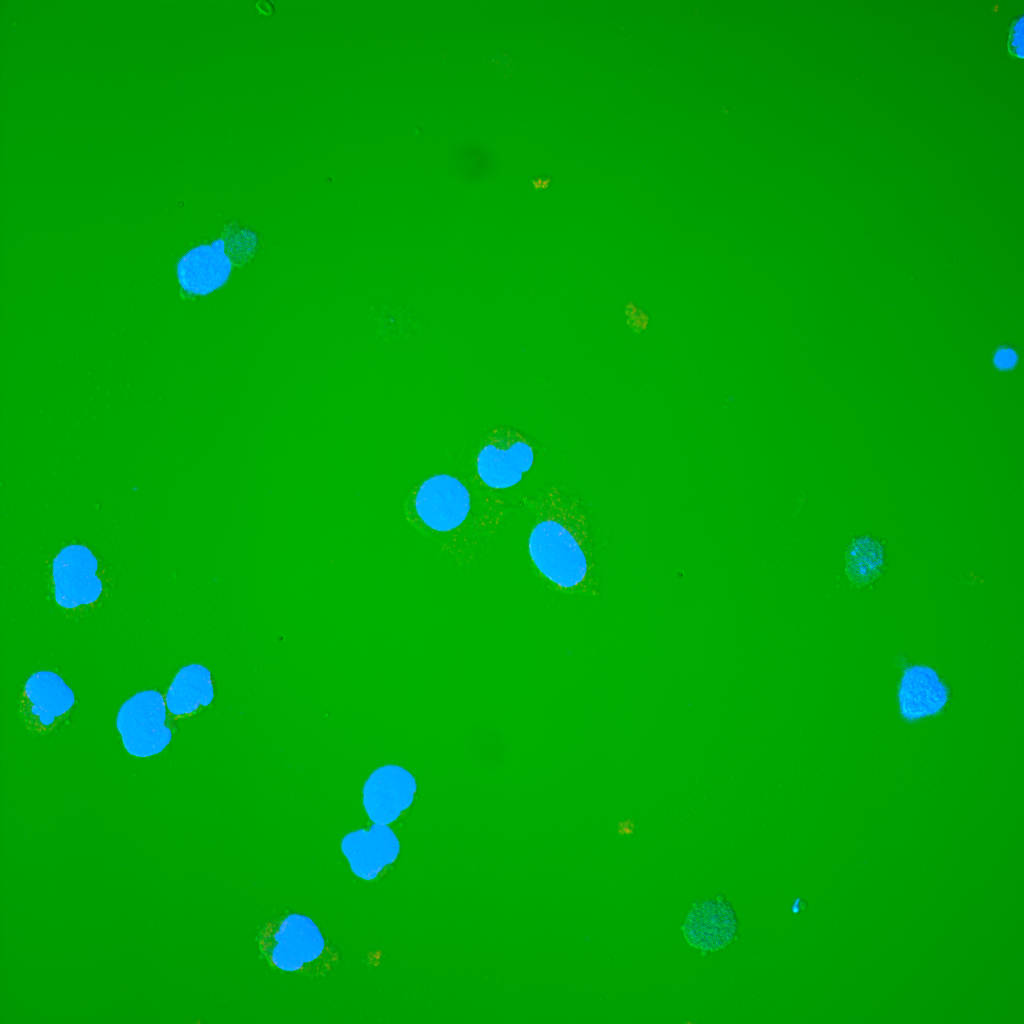

Supplement: Supplementary file 4 — Source data Fig. 1 [file 44319_2025_504_MOESM4_ESM.zip › Figure 1C/PLA Healthy donor_PI3KR4+LRBA.tif]

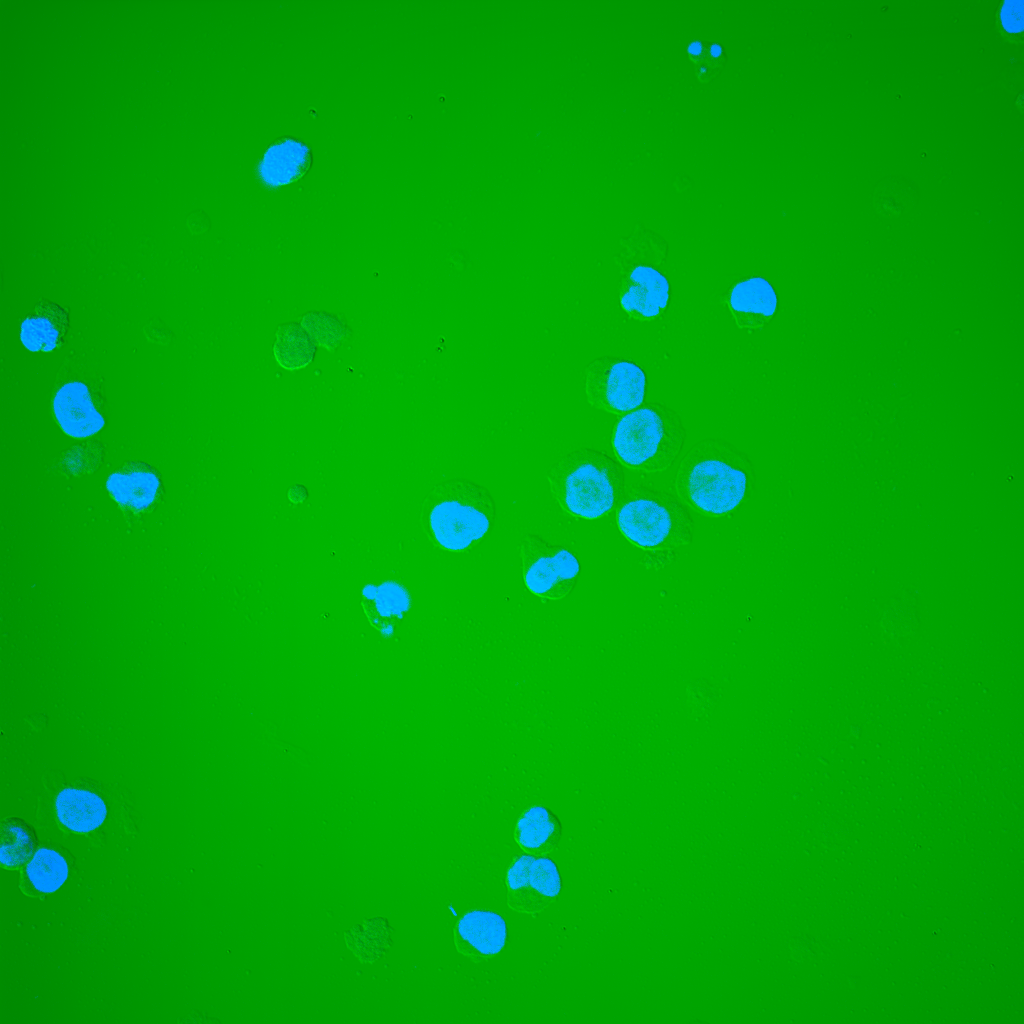

Supplement: Supplementary file 4 — Source data Fig. 1 [file 44319_2025_504_MOESM4_ESM.zip › Figure 1C/PLA LRBA-deficient patient_PI3KR4+LRBA.tif]

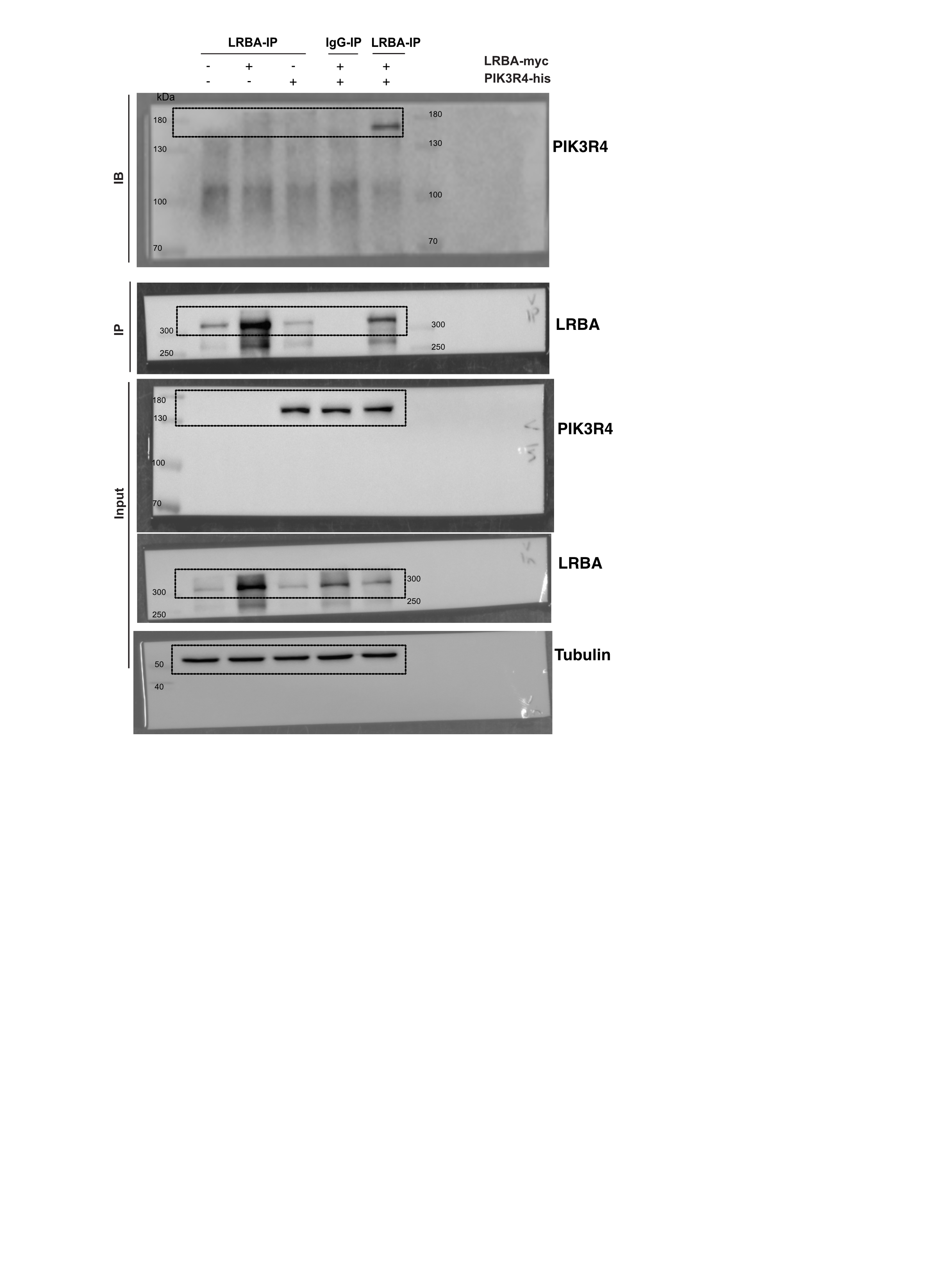

Supplement: Supplementary file 4 — Source data Fig. 1 [file 44319_2025_504_MOESM4_ESM.zip › Figure 1E/Co-IP LRBA and PIK3R4.tiff]

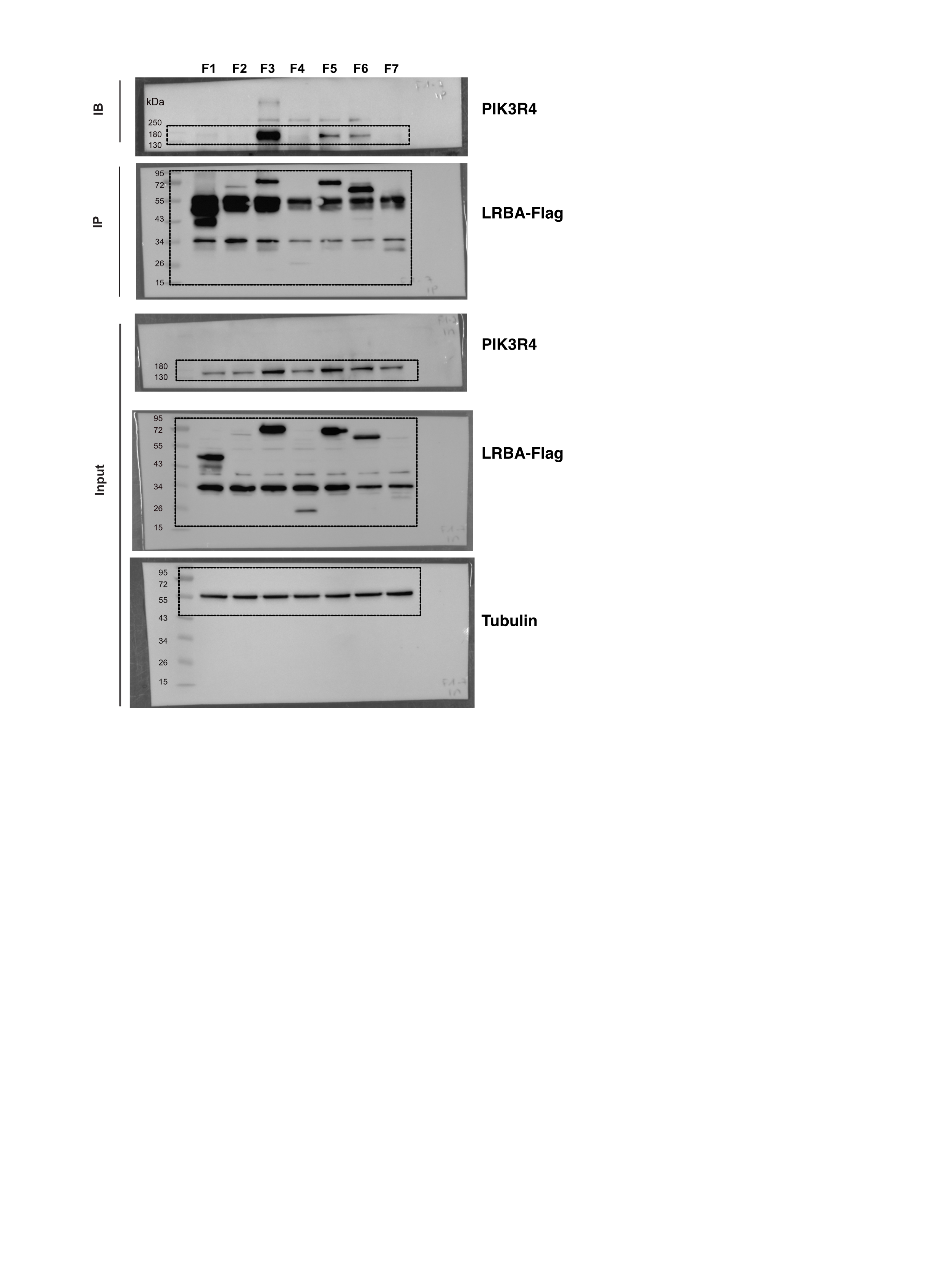

Supplement: Supplementary file 4 — Source data Fig. 1 [file 44319_2025_504_MOESM4_ESM.zip › Figure 1F/Co-IP PIK3R4 and LRBA fragments.tiff]

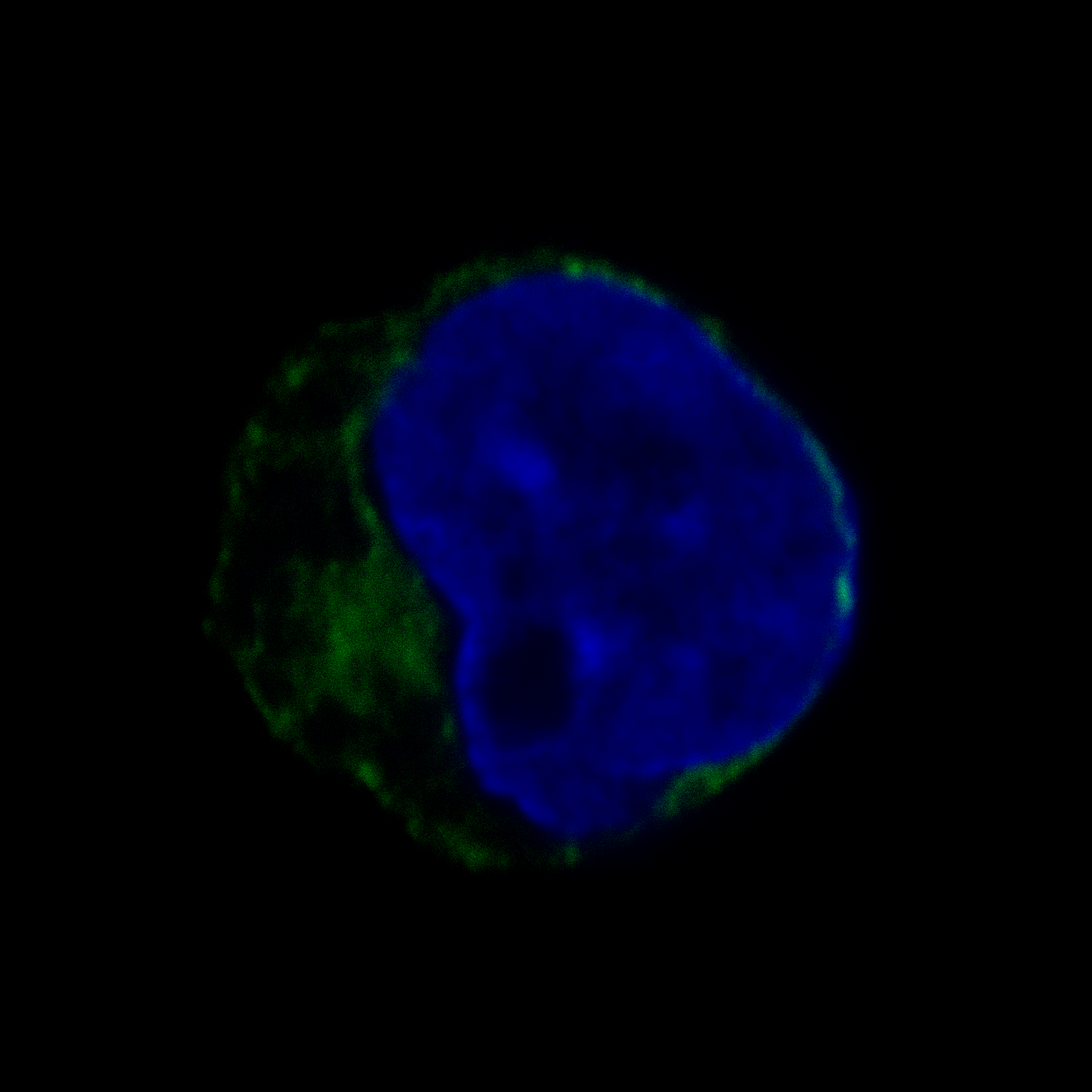

Supplement: Supplementary file 5 — Source data Fig. 2 [file 44319_2025_504_MOESM5_ESM.zip › Figure 2E/HEK LRBA-KO_DFCP1_Basal.tif]

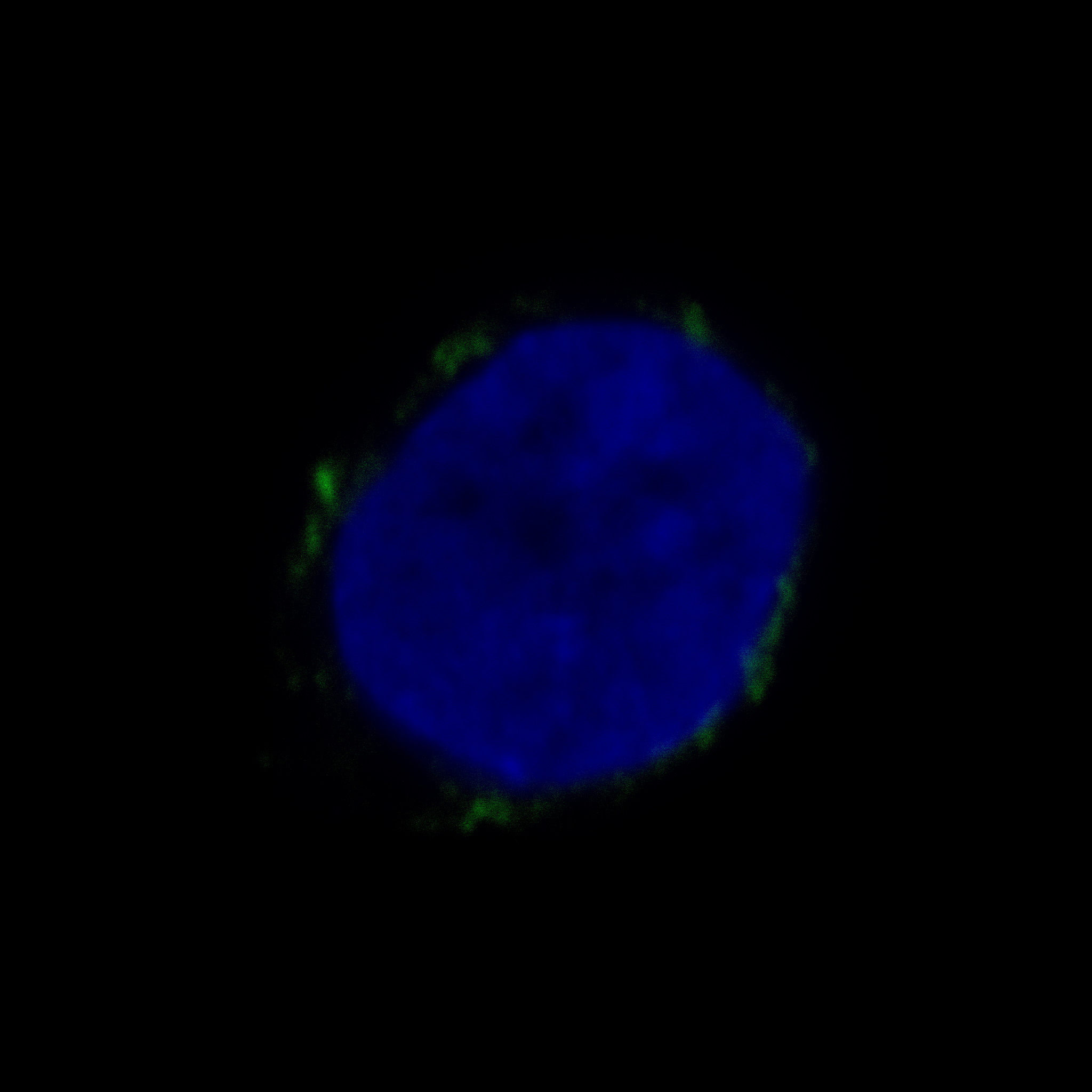

Supplement: Supplementary file 5 — Source data Fig. 2 [file 44319_2025_504_MOESM5_ESM.zip › Figure 2E/HEK LRBA-KO_DFCP1_Rapamycin.tif]

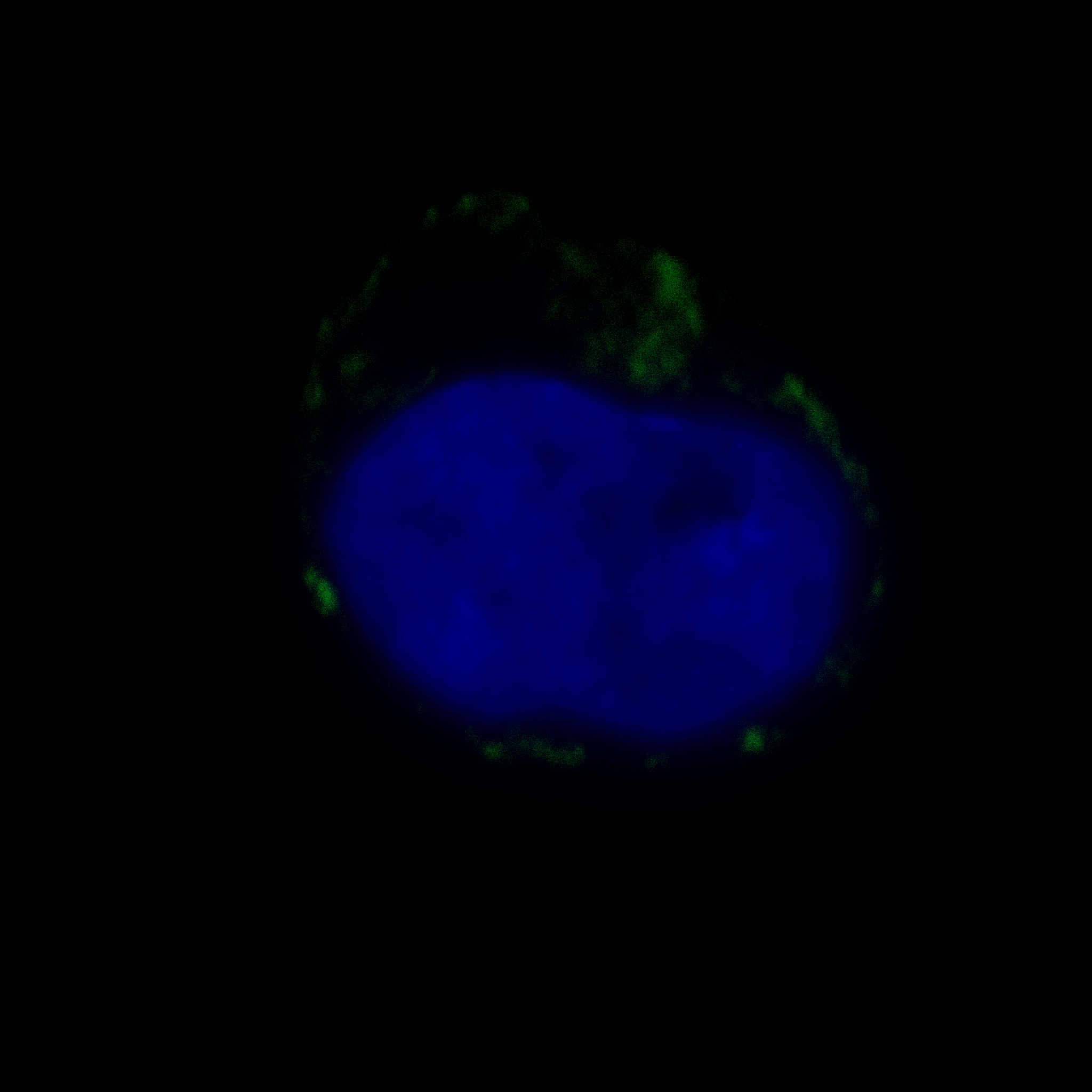

Supplement: Supplementary file 5 — Source data Fig. 2 [file 44319_2025_504_MOESM5_ESM.zip › Figure 2E/HEK LRBA-KO_DFCP1_Torin1.tif]

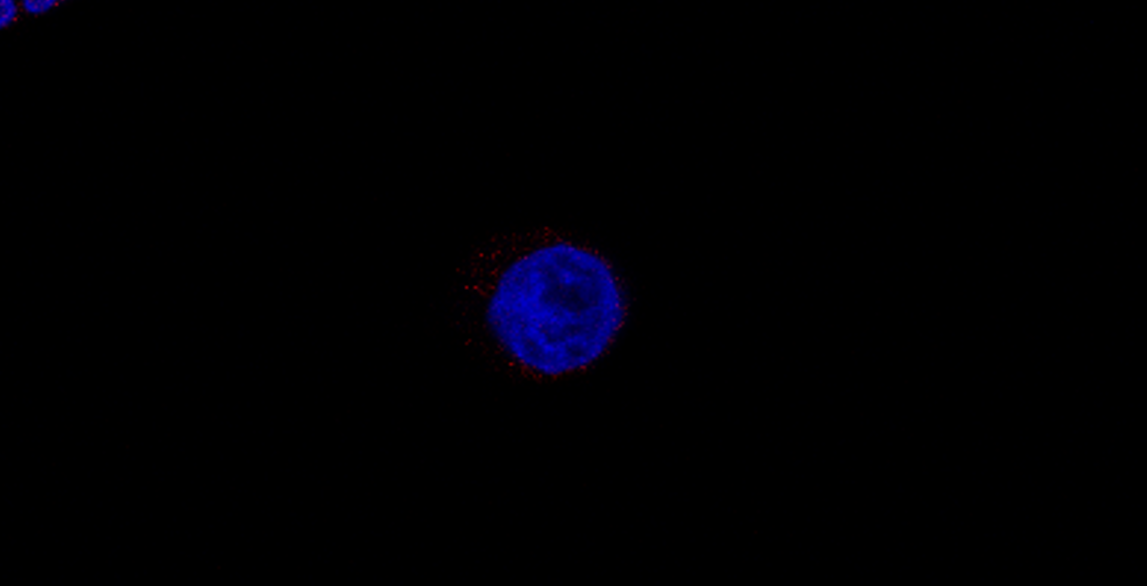

Supplement: Supplementary file 5 — Source data Fig. 2 [file 44319_2025_504_MOESM5_ESM.zip › Figure 2E/HEK LRBA-KO_WIPI2_Basal.tif]

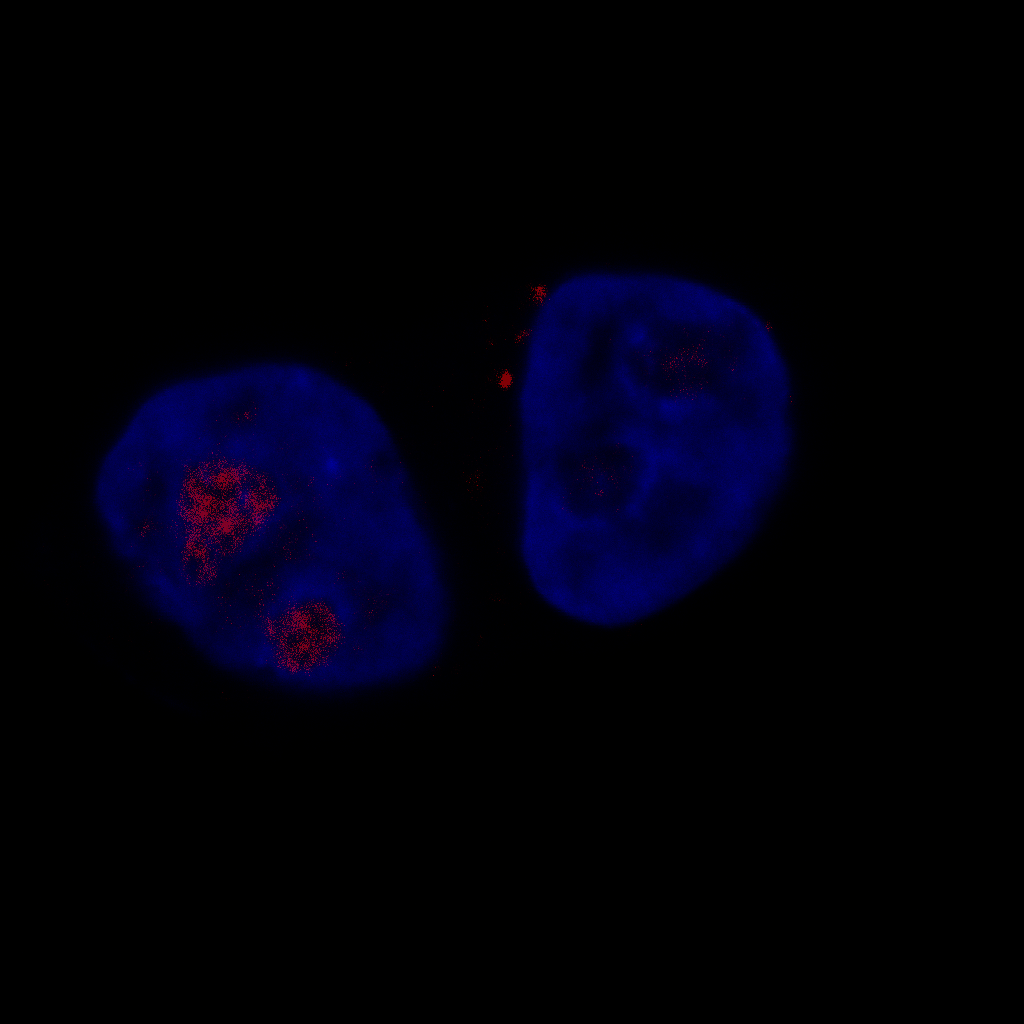

Supplement: Supplementary file 5 — Source data Fig. 2 [file 44319_2025_504_MOESM5_ESM.zip › Figure 2E/HEK LRBA-KO_WIPI2_Rapamycin.tif]

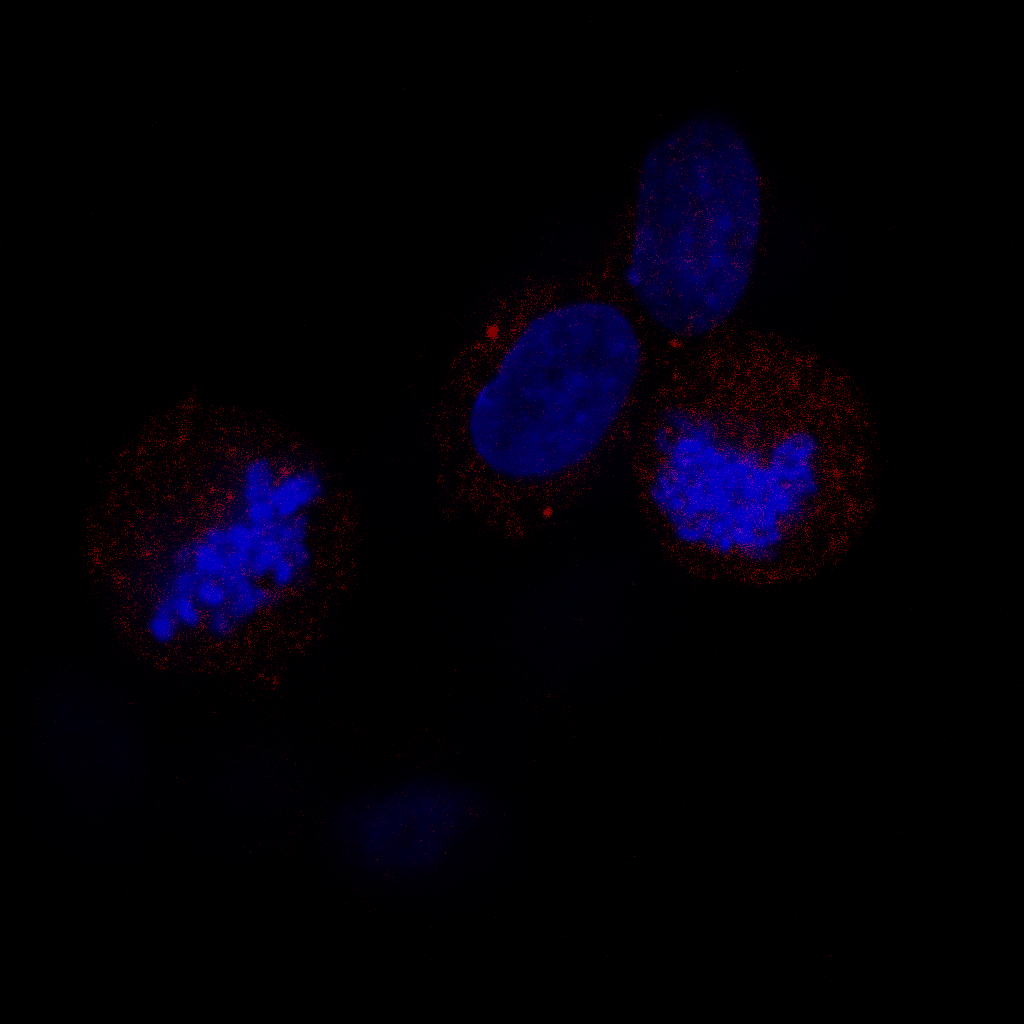

Supplement: Supplementary file 5 — Source data Fig. 2 [file 44319_2025_504_MOESM5_ESM.zip › Figure 2E/HEK LRBA-KO_WIPI2_Torin1.tif]

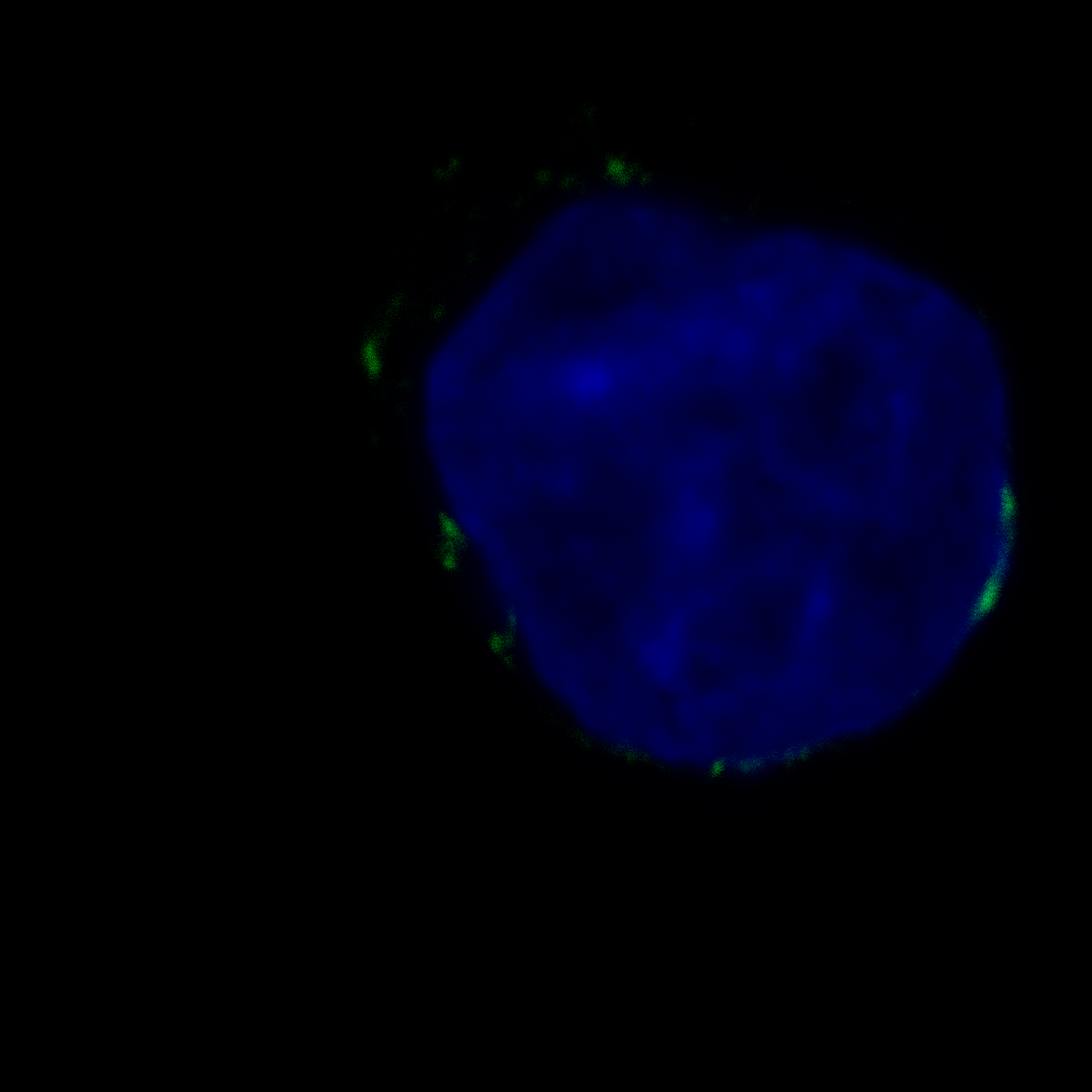

Supplement: Supplementary file 5 — Source data Fig. 2 [file 44319_2025_504_MOESM5_ESM.zip › Figure 2E/HEK WT_DFCP1_Basal.tif]

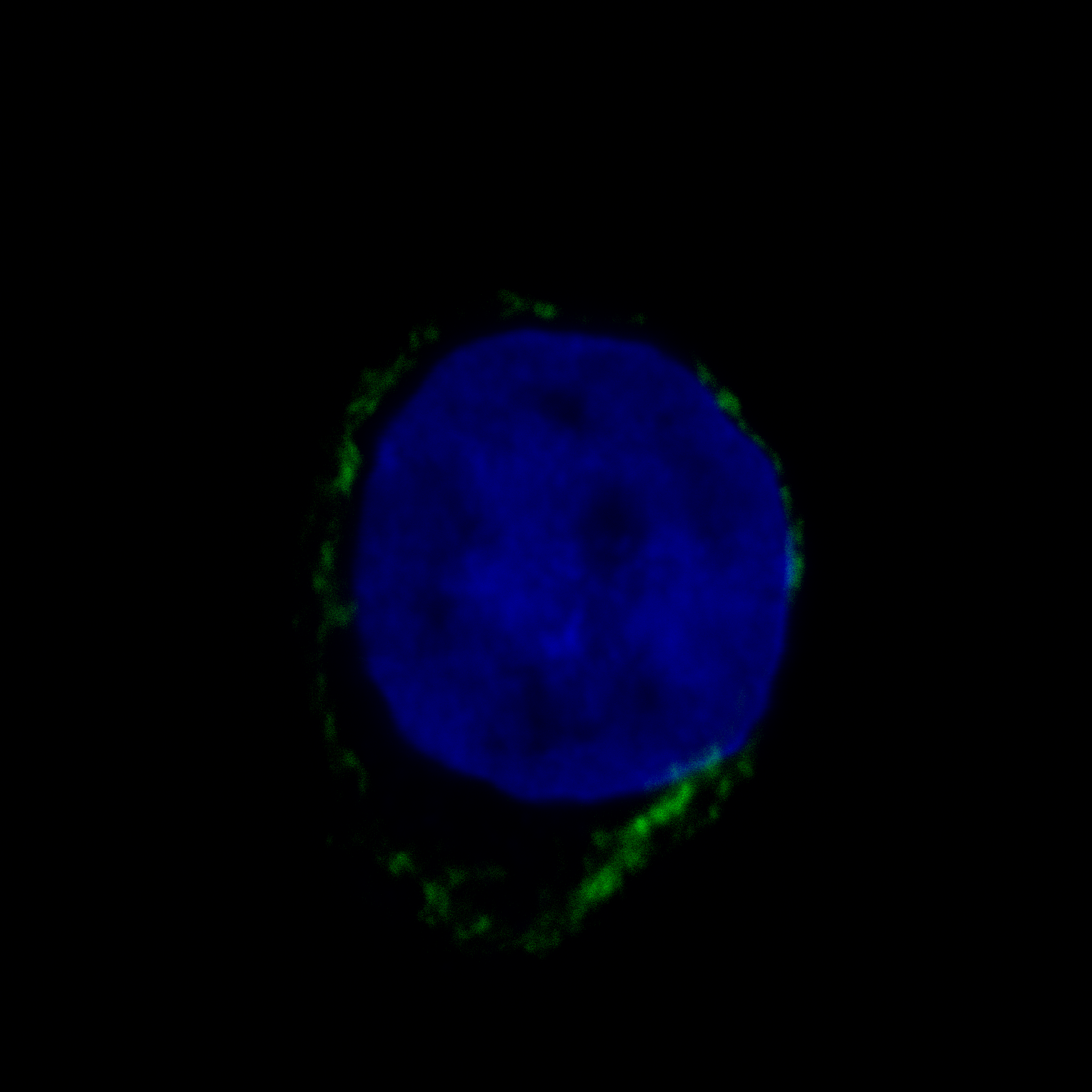

Supplement: Supplementary file 5 — Source data Fig. 2 [file 44319_2025_504_MOESM5_ESM.zip › Figure 2E/HEK WT_DFCP1_Rapamycin.tif]

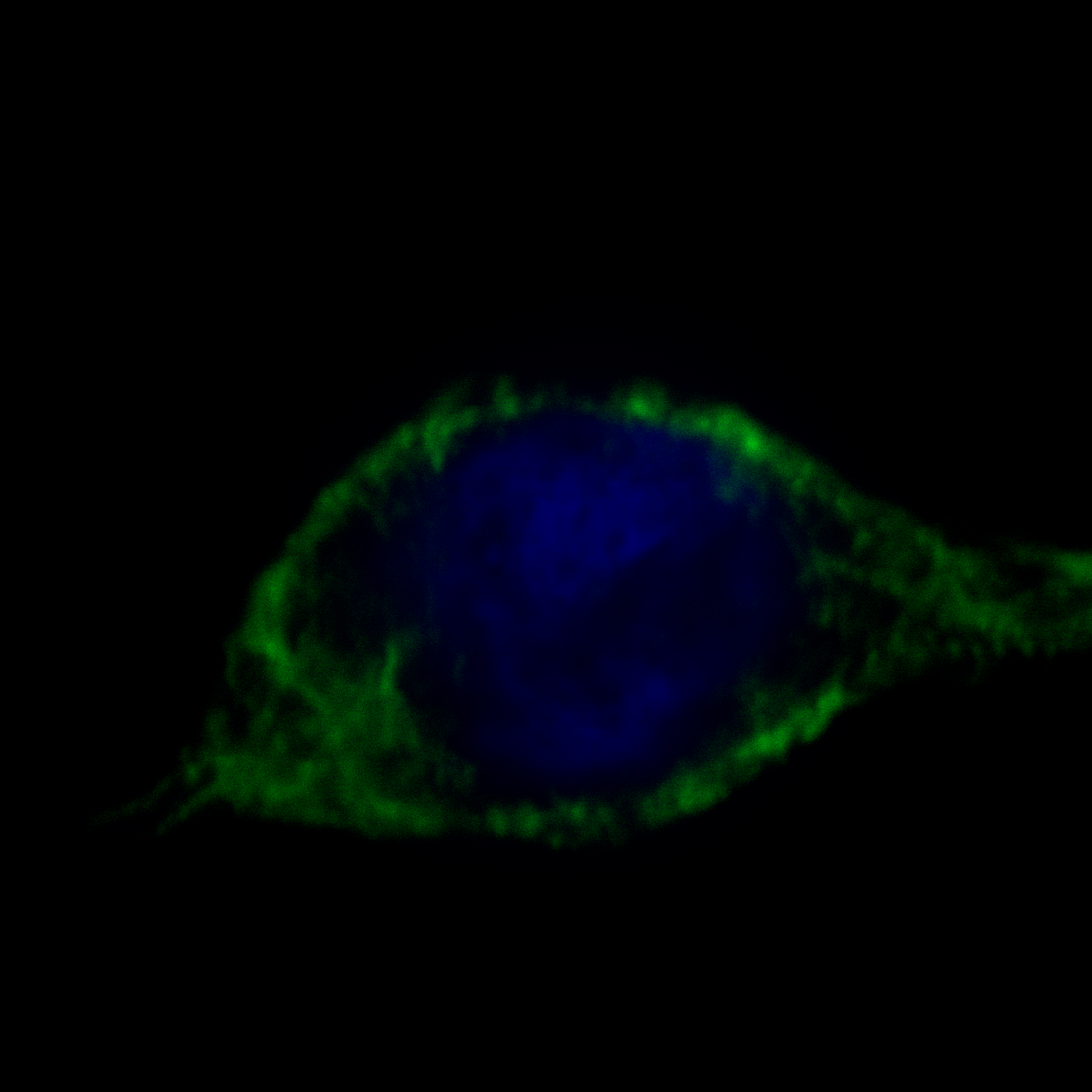

Supplement: Supplementary file 5 — Source data Fig. 2 [file 44319_2025_504_MOESM5_ESM.zip › Figure 2E/HEK WT_DFCP1_Torin1.tif]

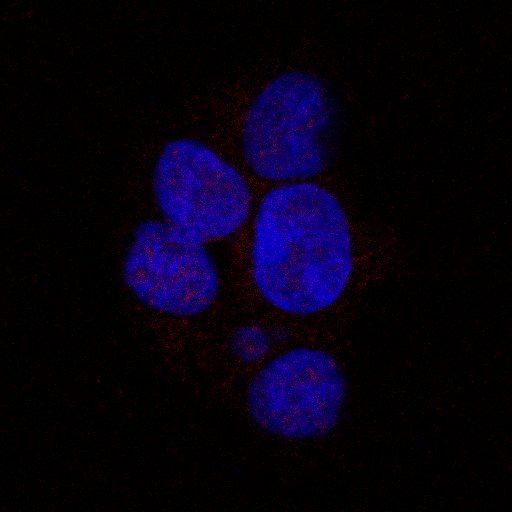

Supplement: Supplementary file 5 — Source data Fig. 2 [file 44319_2025_504_MOESM5_ESM.zip › Figure 2E/HEK WT_WIPI2_Basal.tif]

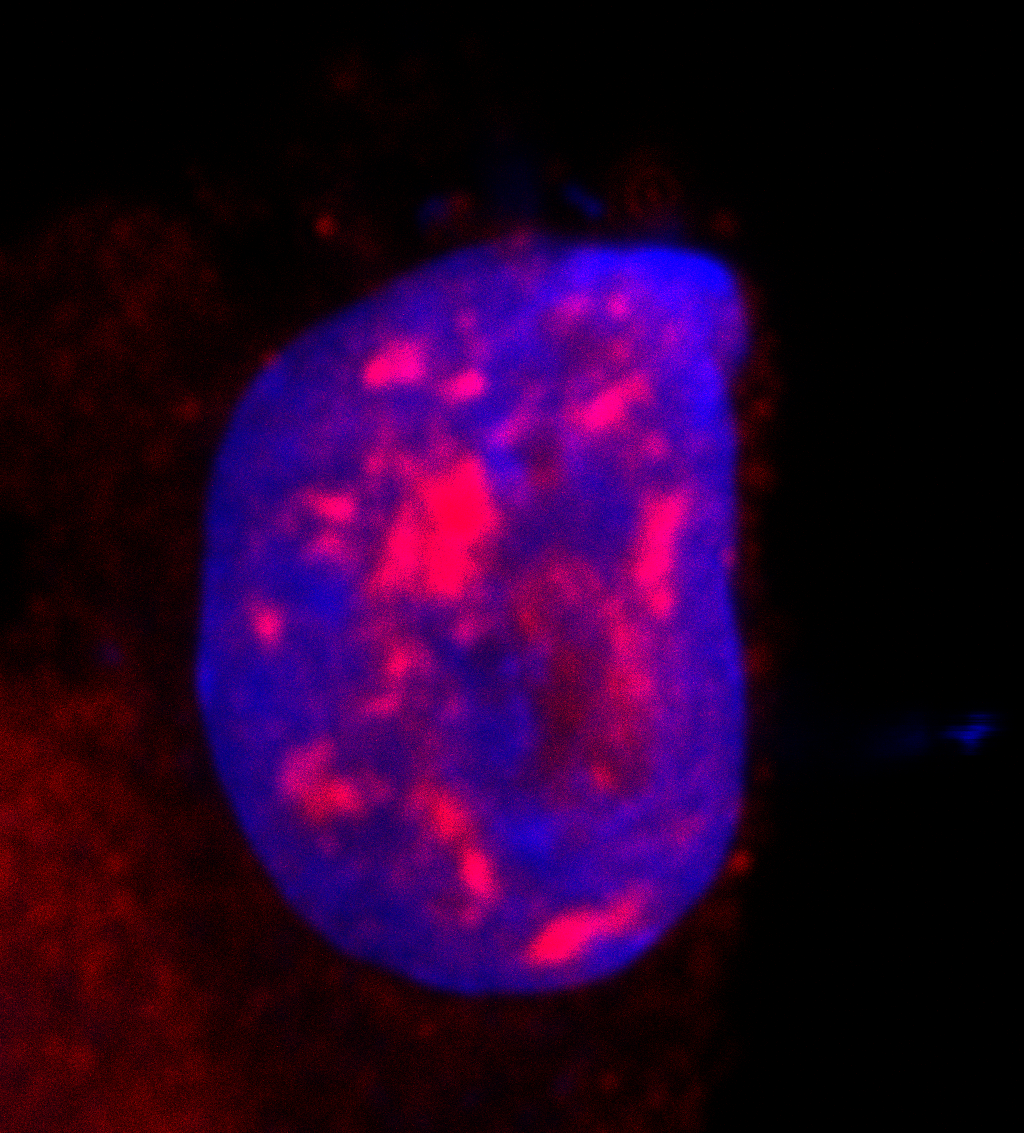

Supplement: Supplementary file 5 — Source data Fig. 2 [file 44319_2025_504_MOESM5_ESM.zip › Figure 2E/HEK WT_WIPI2_Rapamycin.tif]

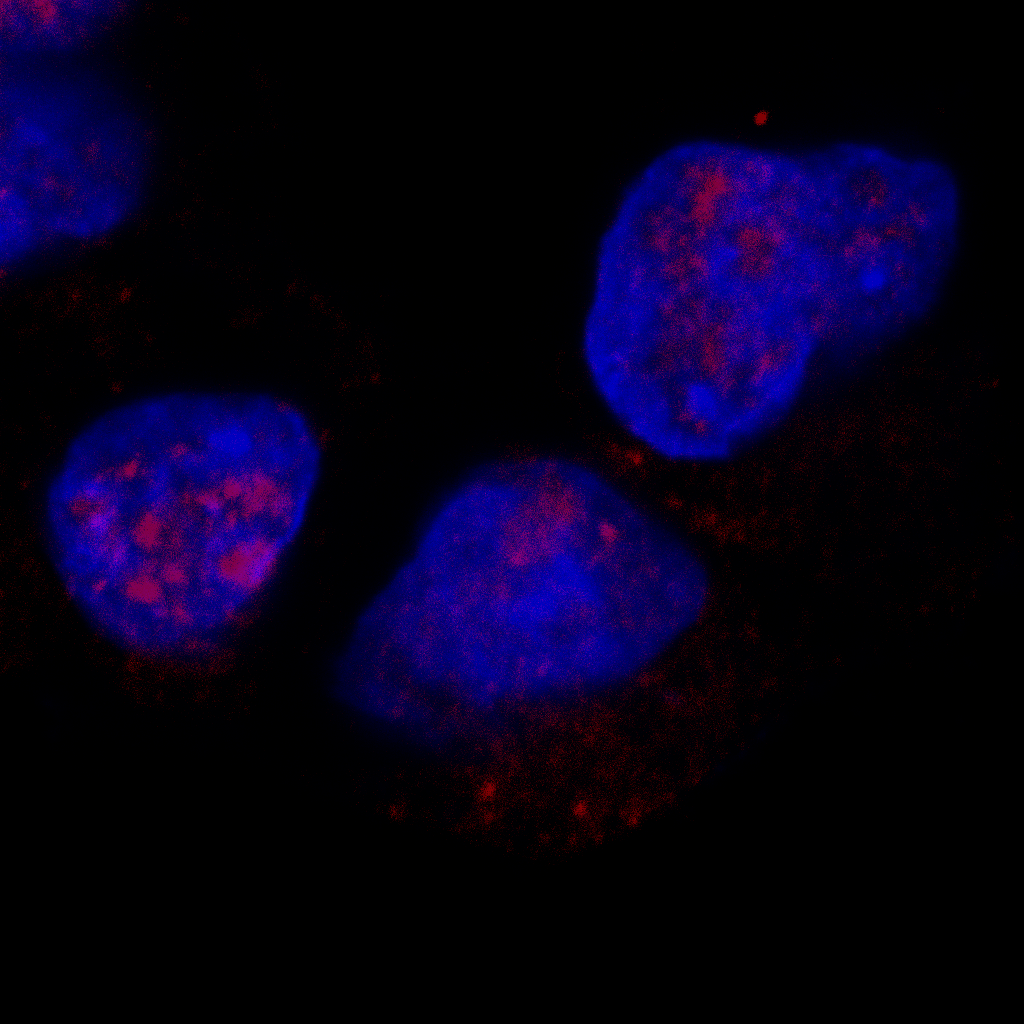

Supplement: Supplementary file 5 — Source data Fig. 2 [file 44319_2025_504_MOESM5_ESM.zip › Figure 2E/HEK WT_WIPI2_Torin1.tif]

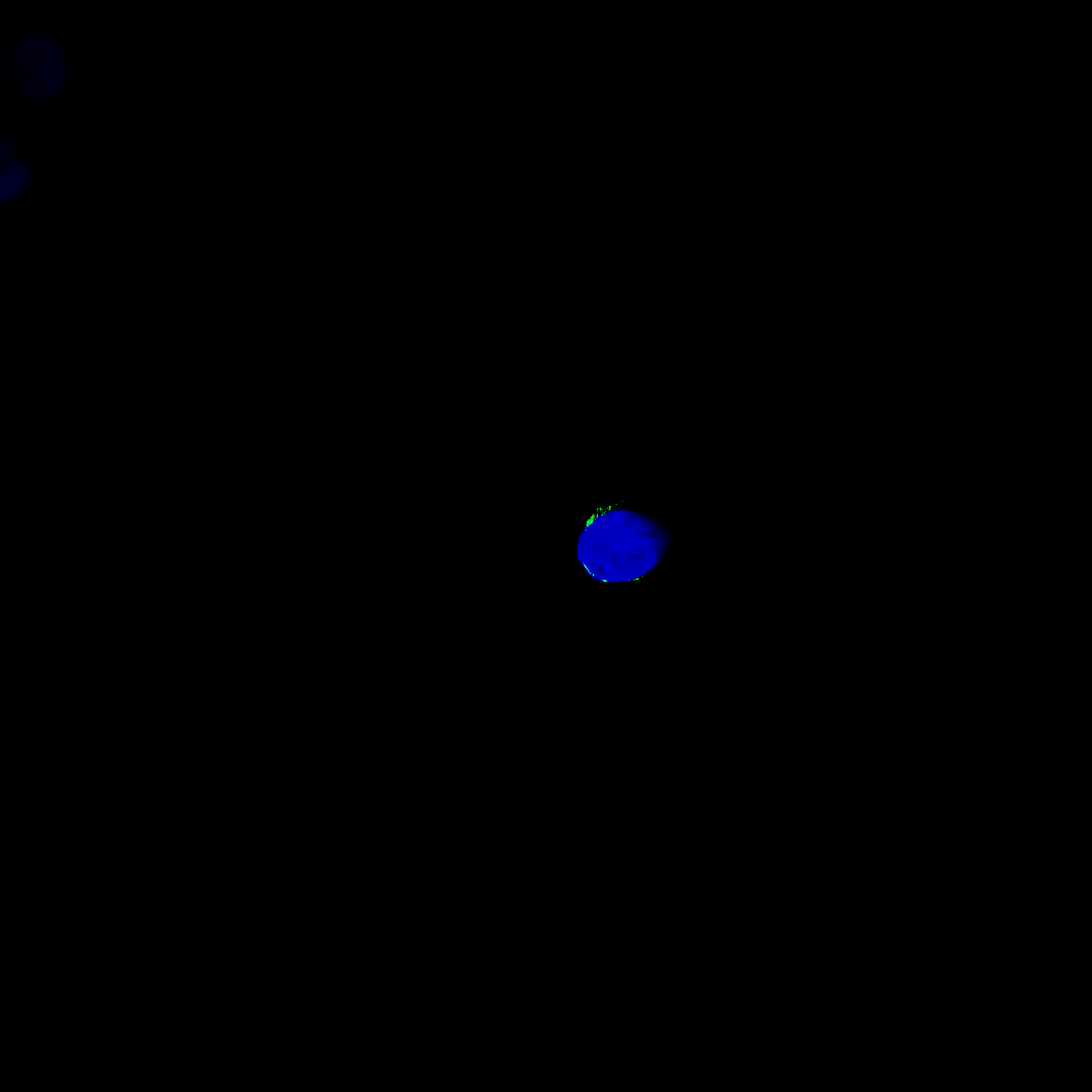

Supplement: Supplementary file 5 — Source data Fig. 2 [file 44319_2025_504_MOESM5_ESM.zip › Figure 2F/HEK LRBA-KO_DFCP1_Torin1.tif]

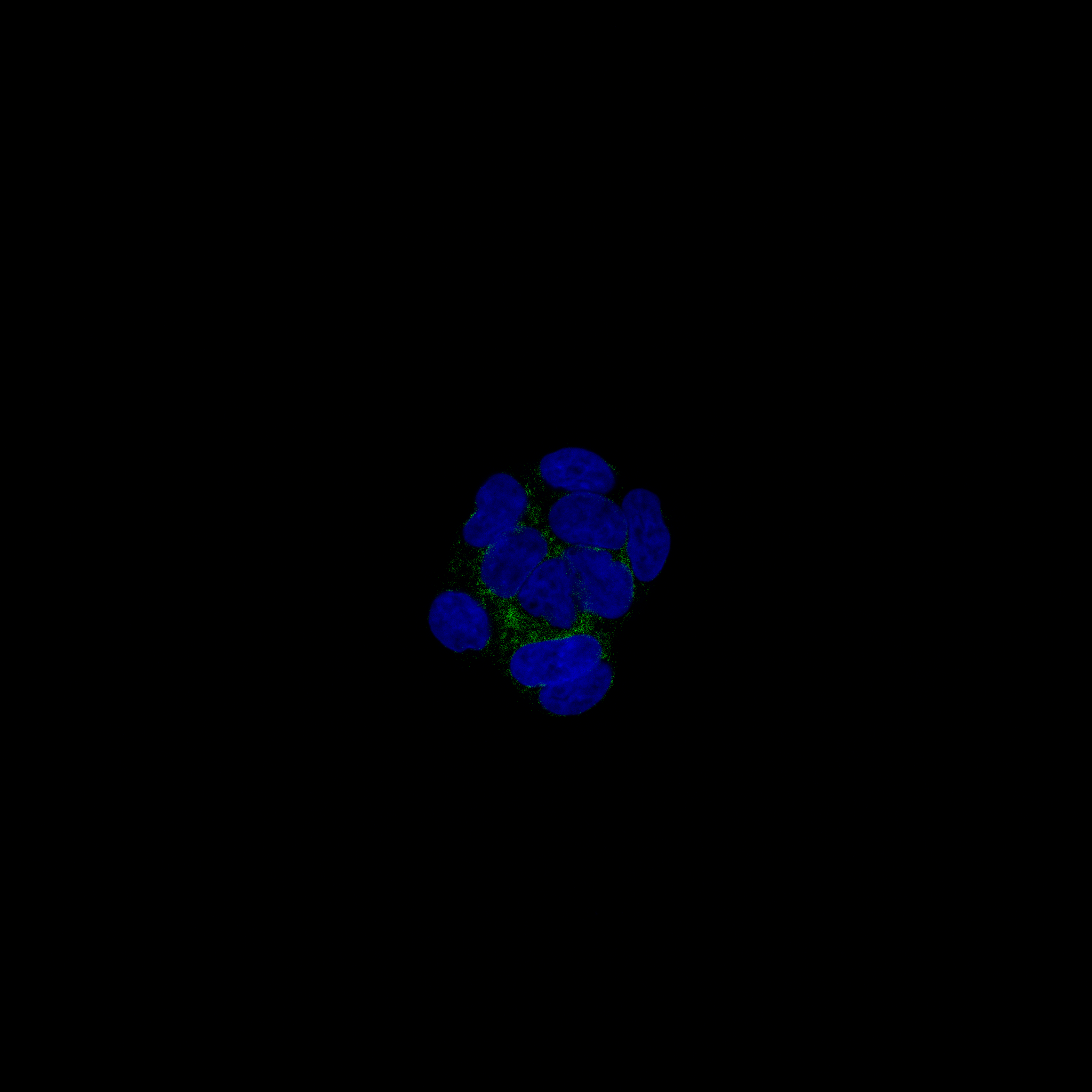

Supplement: Supplementary file 5 — Source data Fig. 2 [file 44319_2025_504_MOESM5_ESM.zip › Figure 2F/HEK LRBA-KO_DFCP1_Torin1+0.01uM VPS34-IN1.tif]

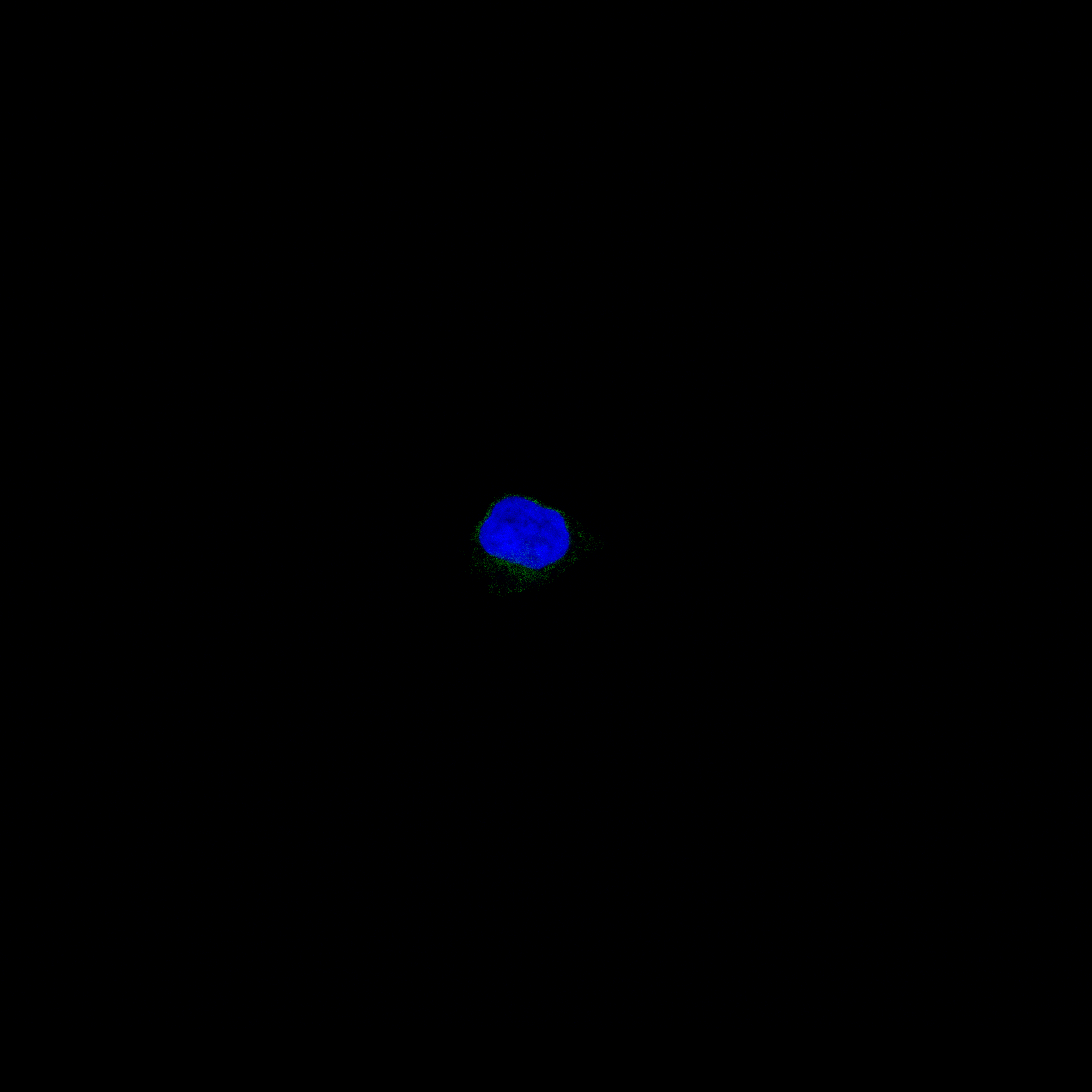

Supplement: Supplementary file 5 — Source data Fig. 2 [file 44319_2025_504_MOESM5_ESM.zip › Figure 2F/HEK LRBA-KO_DFCP1_Torin1+0.1uM VPS34-IN1.tif]

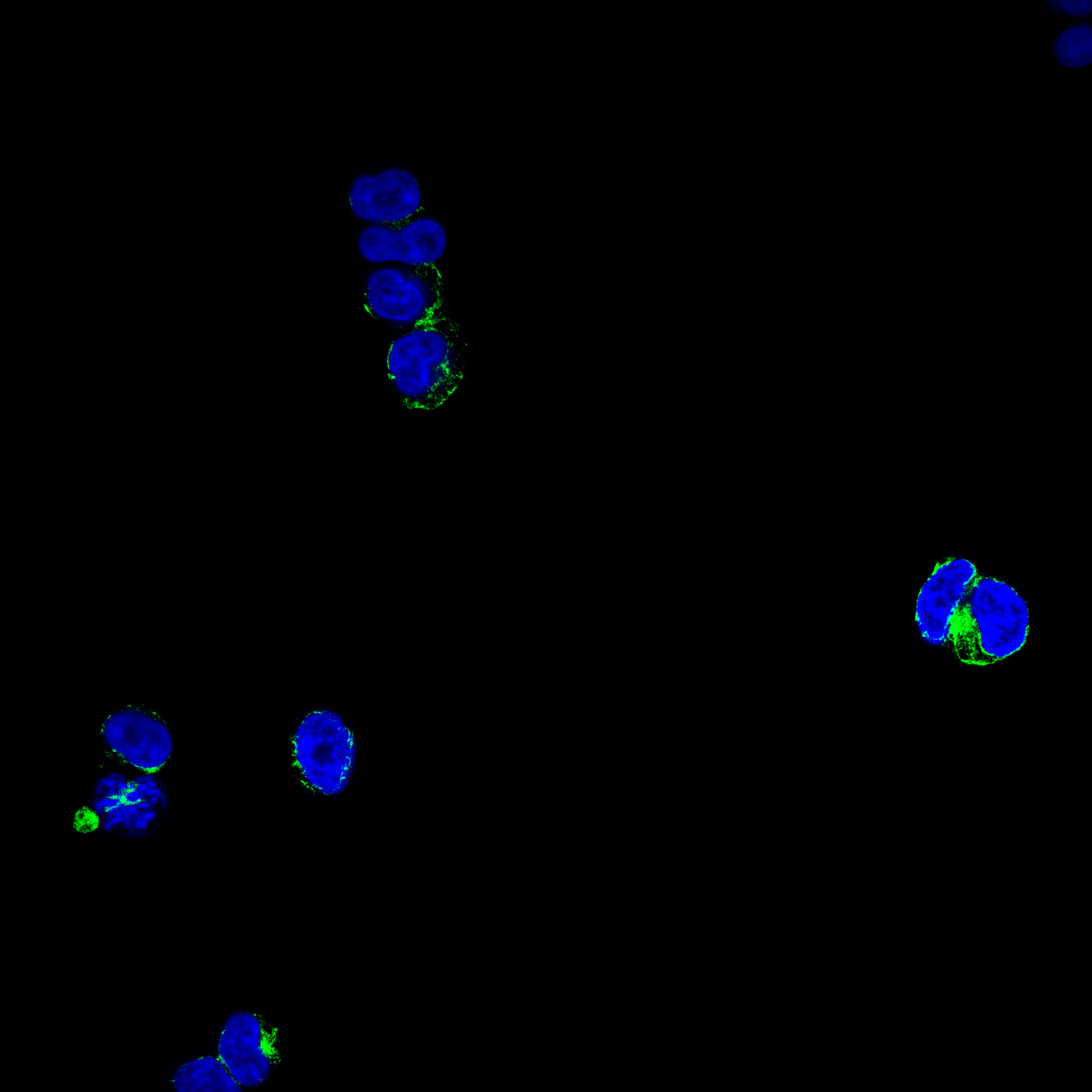

Supplement: Supplementary file 5 — Source data Fig. 2 [file 44319_2025_504_MOESM5_ESM.zip › Figure 2F/HEK WT_DFCP1_Torin1.tif]

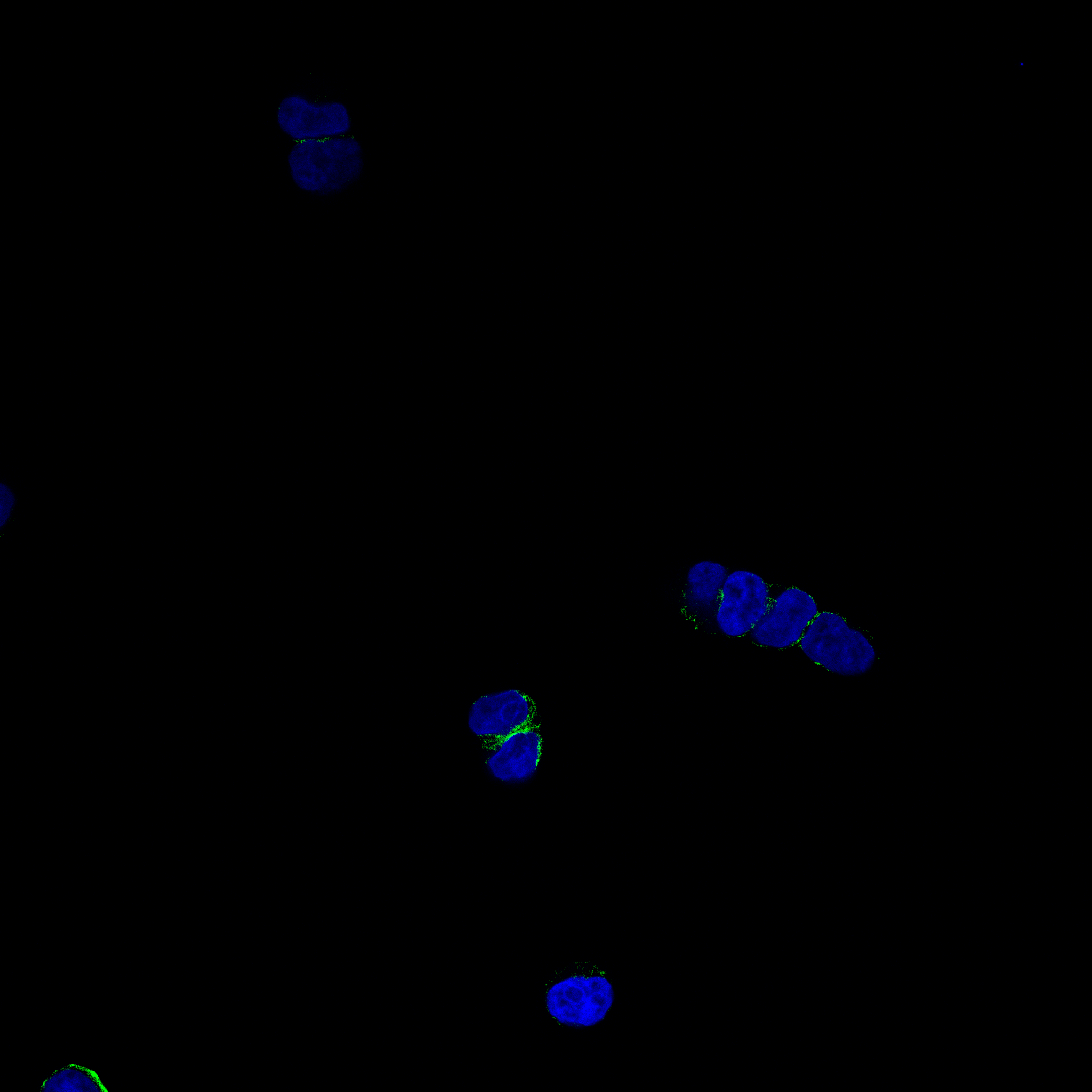

Supplement: Supplementary file 5 — Source data Fig. 2 [file 44319_2025_504_MOESM5_ESM.zip › Figure 2F/HEK WT_DFCP1_Torin1+0.01uM VPS34-IN1.tif]

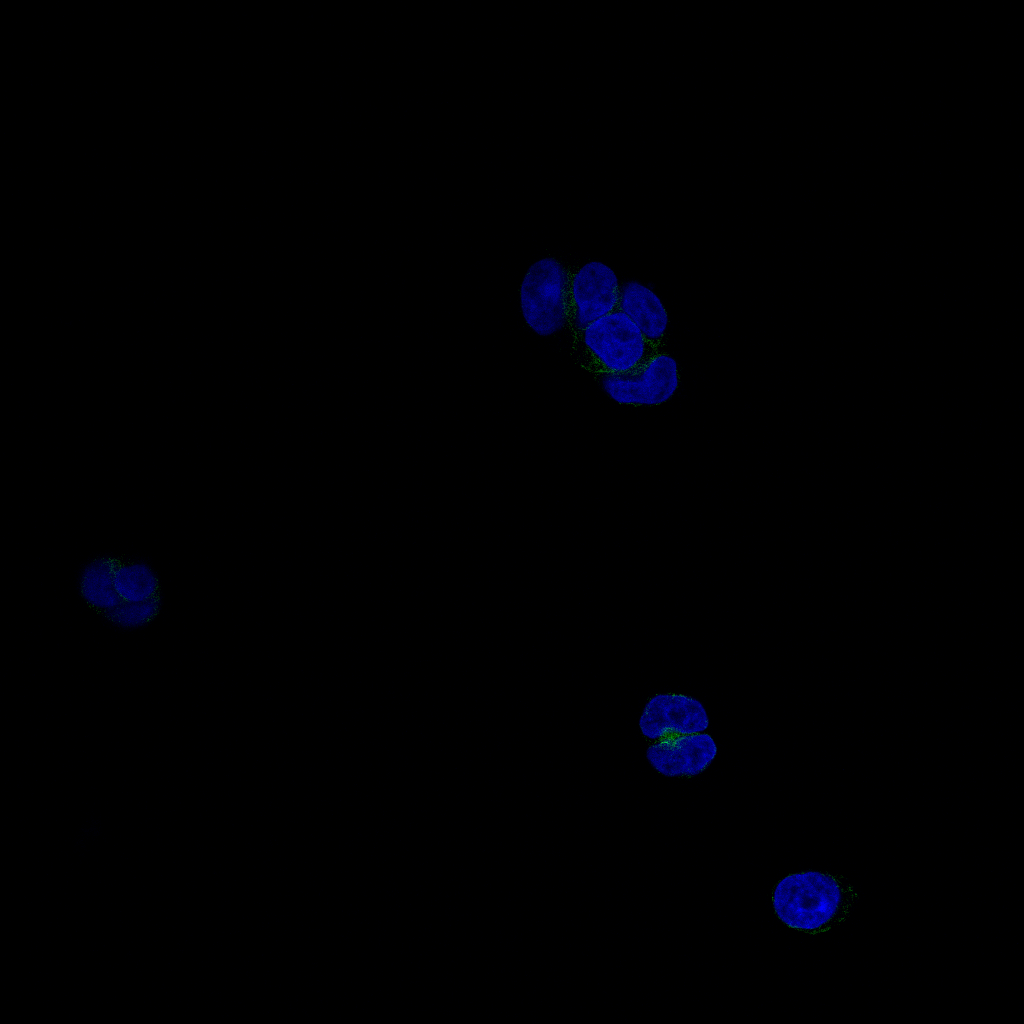

Supplement: Supplementary file 5 — Source data Fig. 2 [file 44319_2025_504_MOESM5_ESM.zip › Figure 2F/HEK WT_DFCP1_Torin1+0.1uM VPS34-IN1.tif]

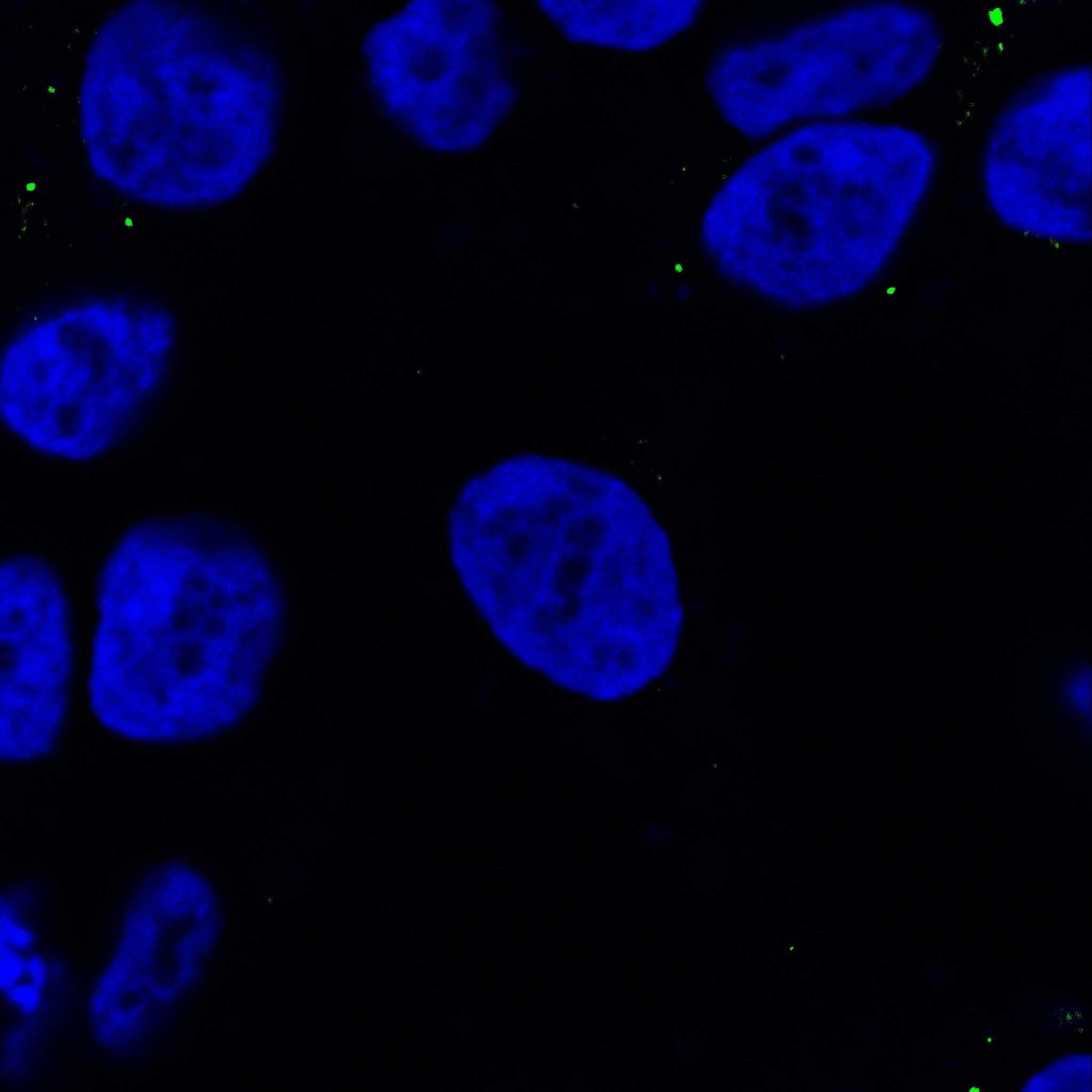

Supplement: Supplementary file 5 — Source data Fig. 2 [file 44319_2025_504_MOESM5_ESM.zip › Figure 2H/HEK293T LRBA-KO_Basal_DFCP1+DAPI.tif]

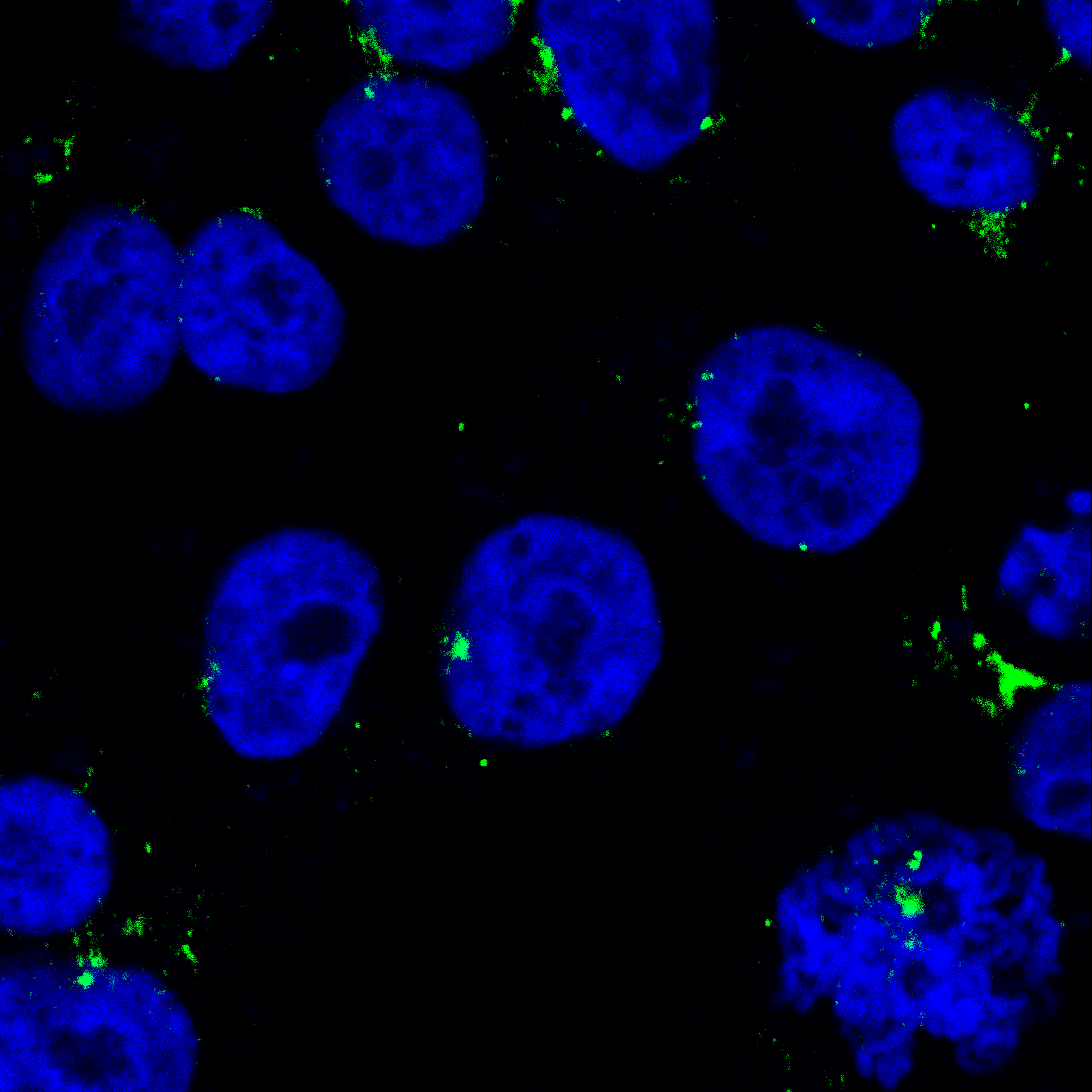

Supplement: Supplementary file 5 — Source data Fig. 2 [file 44319_2025_504_MOESM5_ESM.zip › Figure 2H/HEK293T LRBA-KO_Torin1_DFCP1+DAPI.tif]

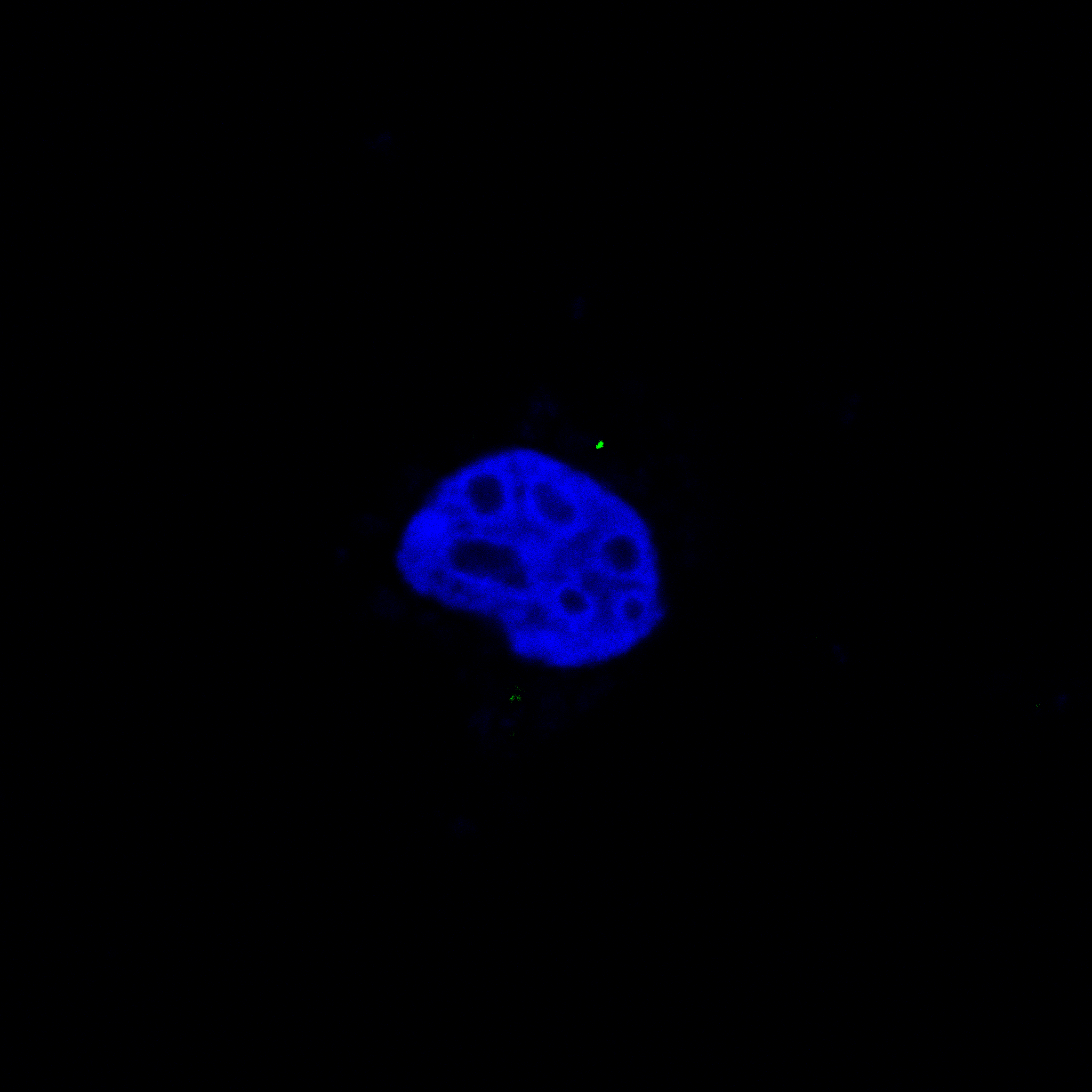

Supplement: Supplementary file 5 — Source data Fig. 2 [file 44319_2025_504_MOESM5_ESM.zip › Figure 2H/HEK293T Myc-LRBA_Basal_DFCP1+DAPI.tif]

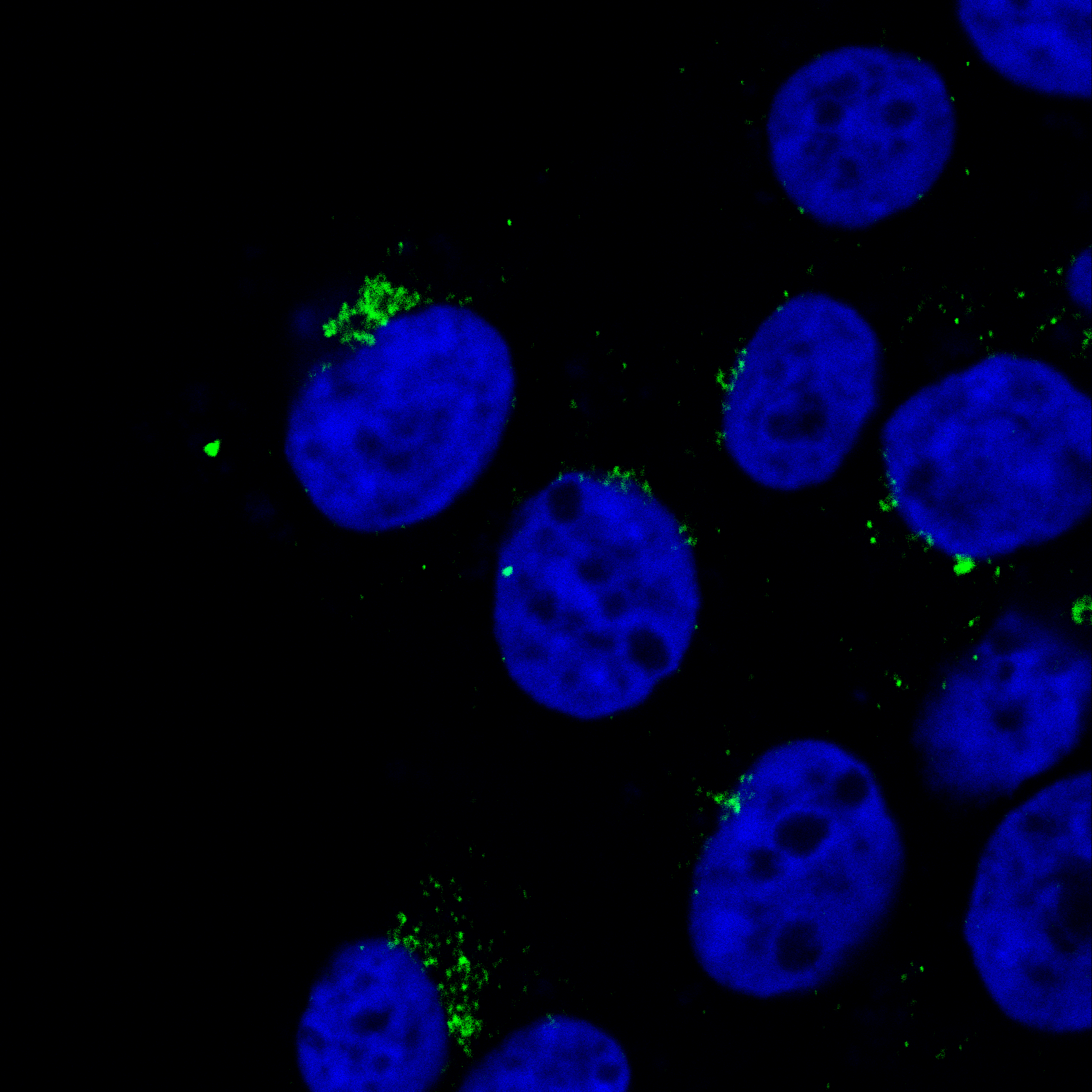

Supplement: Supplementary file 5 — Source data Fig. 2 [file 44319_2025_504_MOESM5_ESM.zip › Figure 2H/HEK293T Myc-LRBA_Torin1_DFCP1+DAPI.tif]

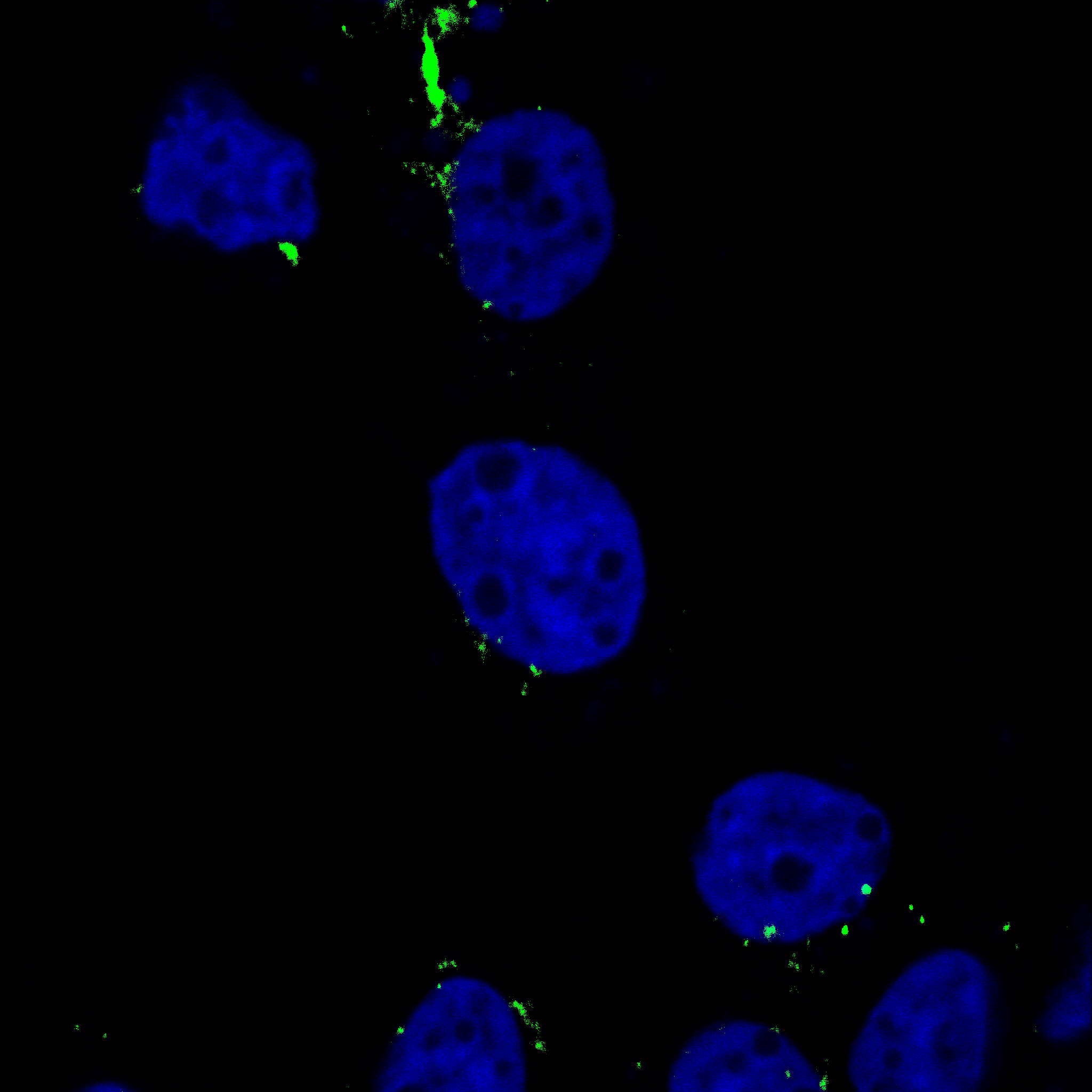

Supplement: Supplementary file 5 — Source data Fig. 2 [file 44319_2025_504_MOESM5_ESM.zip › Figure 2H/HEK293T WT_Basal_DFCP1+DAPI.tif]

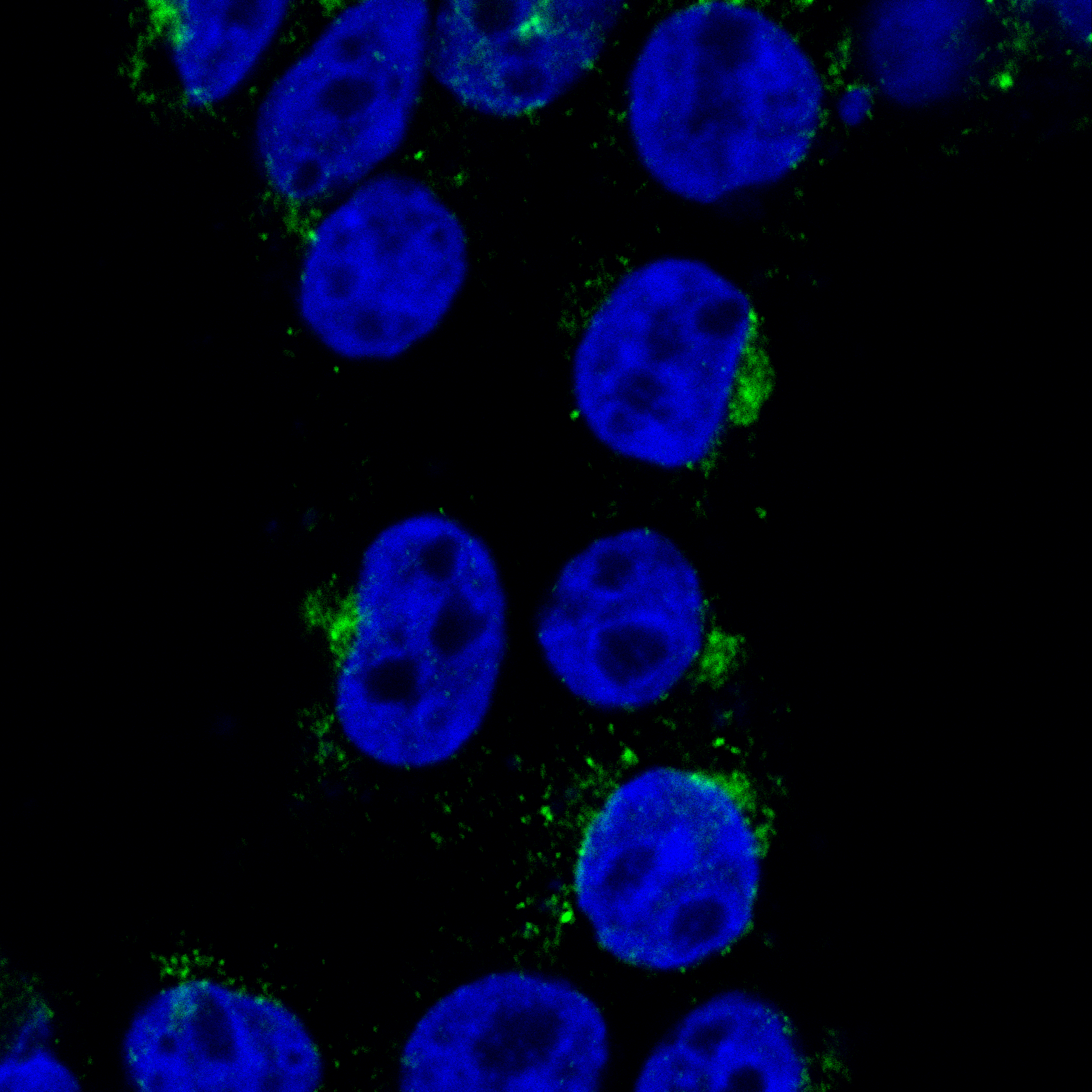

Supplement: Supplementary file 5 — Source data Fig. 2 [file 44319_2025_504_MOESM5_ESM.zip › Figure 2H/HEK293T WT_Torin1_DFCP1+DAPI.tif]

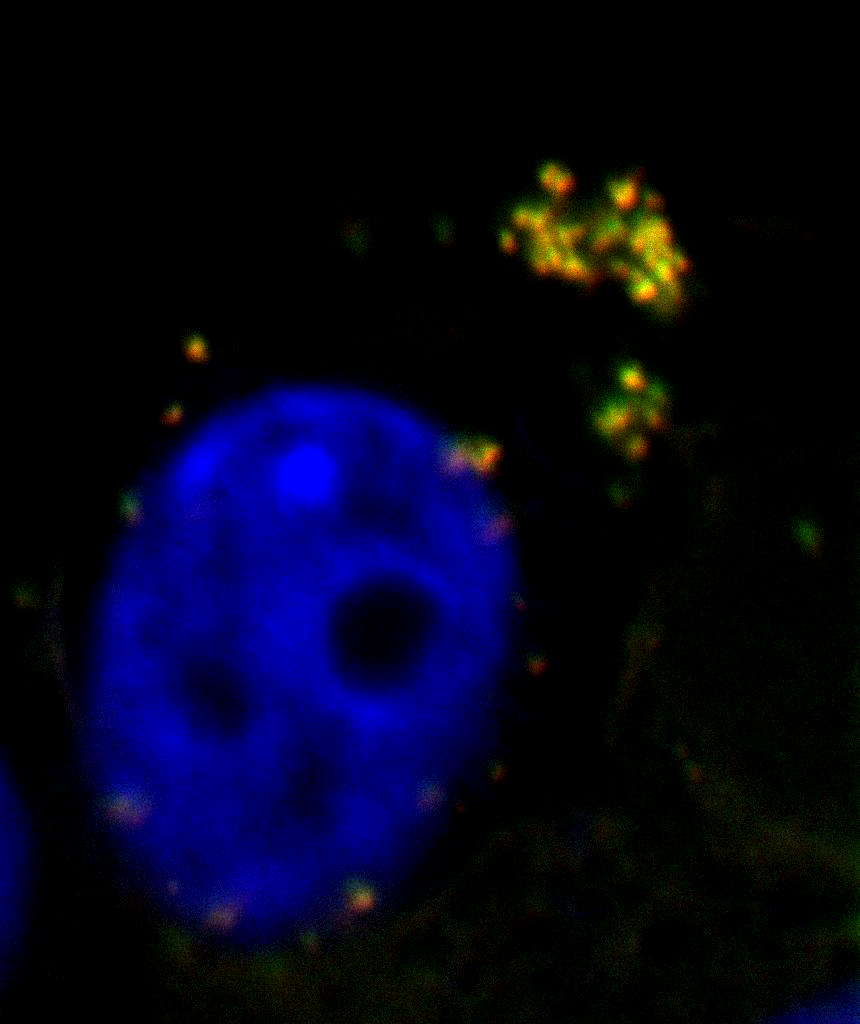

Supplement: Supplementary file 6 — Source data Fig. 3 [file 44319_2025_504_MOESM6_ESM.zip › Figure 3A/LRBA-KO_Bafilomycin A1_GFP-LC3-mCherry.tif]

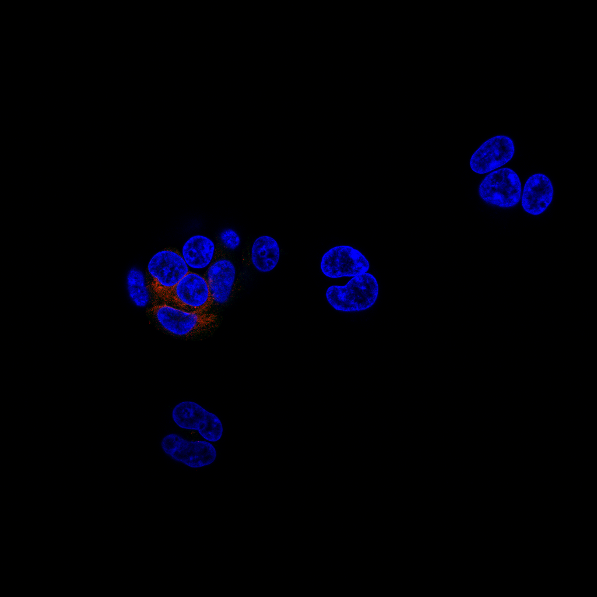

Supplement: Supplementary file 6 — Source data Fig. 3 [file 44319_2025_504_MOESM6_ESM.zip › Figure 3A/LRBA-KO_Basal_GFP-LC3-mCherry.tif]

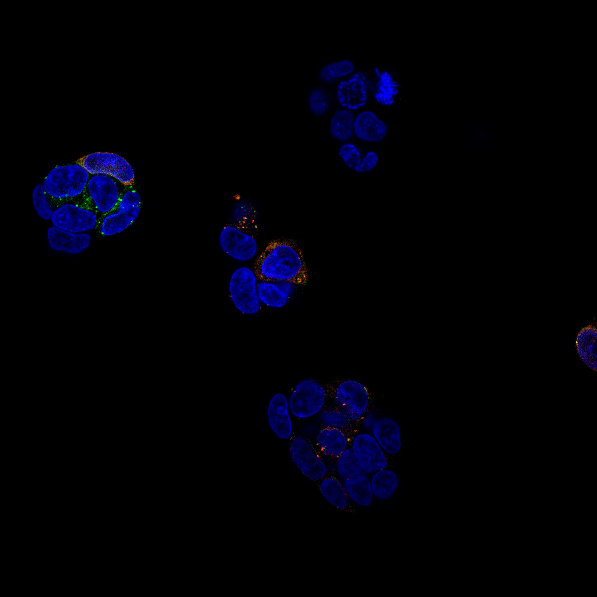

Supplement: Supplementary file 6 — Source data Fig. 3 [file 44319_2025_504_MOESM6_ESM.zip › Figure 3A/LRBA-KO_Torin1_GFP-LC3-mCherry.tif]

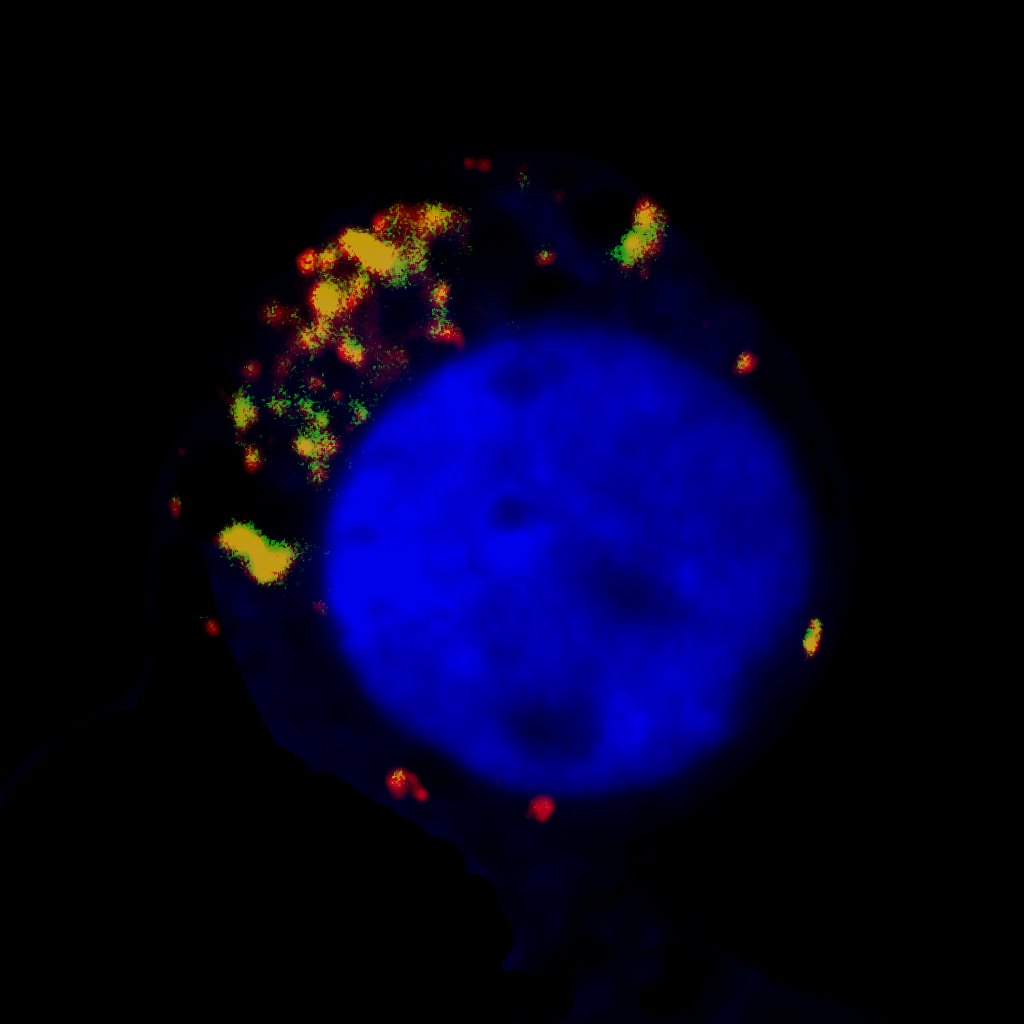

Supplement: Supplementary file 6 — Source data Fig. 3 [file 44319_2025_504_MOESM6_ESM.zip › Figure 3A/LRBA-KO_Torin1+Bafilomycin A1_GFP-LC3-mCherry.tif]

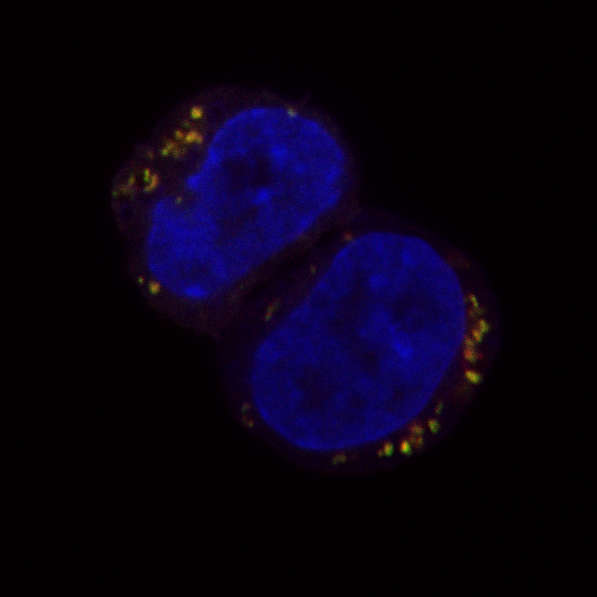

Supplement: Supplementary file 6 — Source data Fig. 3 [file 44319_2025_504_MOESM6_ESM.zip › Figure 3A/WT_Bafilomycin A1_GFP-LC3-mCherry.tif]

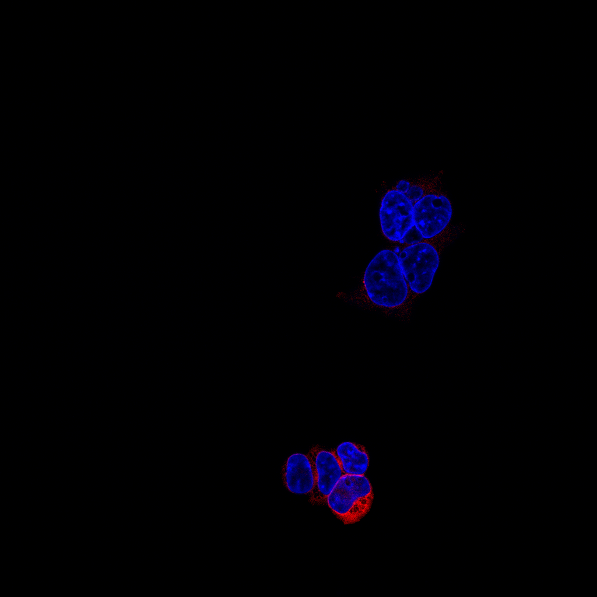

Supplement: Supplementary file 6 — Source data Fig. 3 [file 44319_2025_504_MOESM6_ESM.zip › Figure 3A/WT_Basal_GFP-LC3-mCherry.tif]

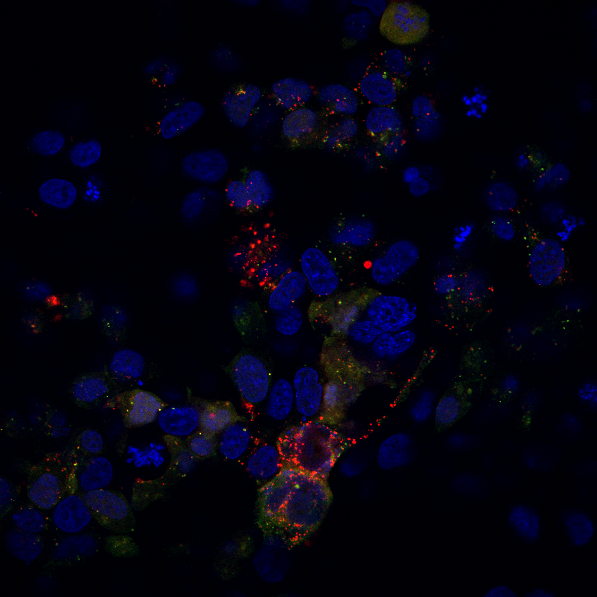

Supplement: Supplementary file 6 — Source data Fig. 3 [file 44319_2025_504_MOESM6_ESM.zip › Figure 3A/WT_Torin1_GFP-LC3-mCherry.tif]

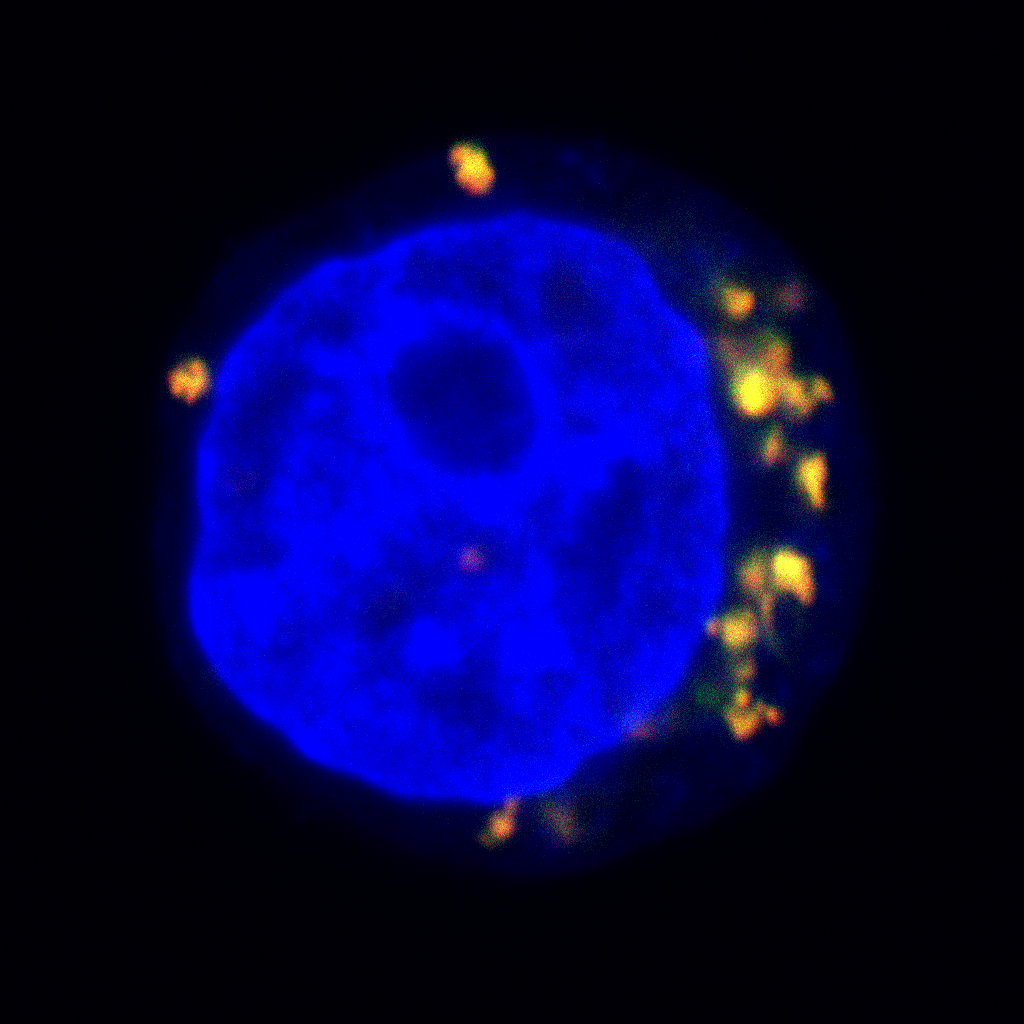

Supplement: Supplementary file 6 — Source data Fig. 3 [file 44319_2025_504_MOESM6_ESM.zip › Figure 3A/WT_Torin1+Bafilomycin A1_GFP-LC3-mCherry.tif]

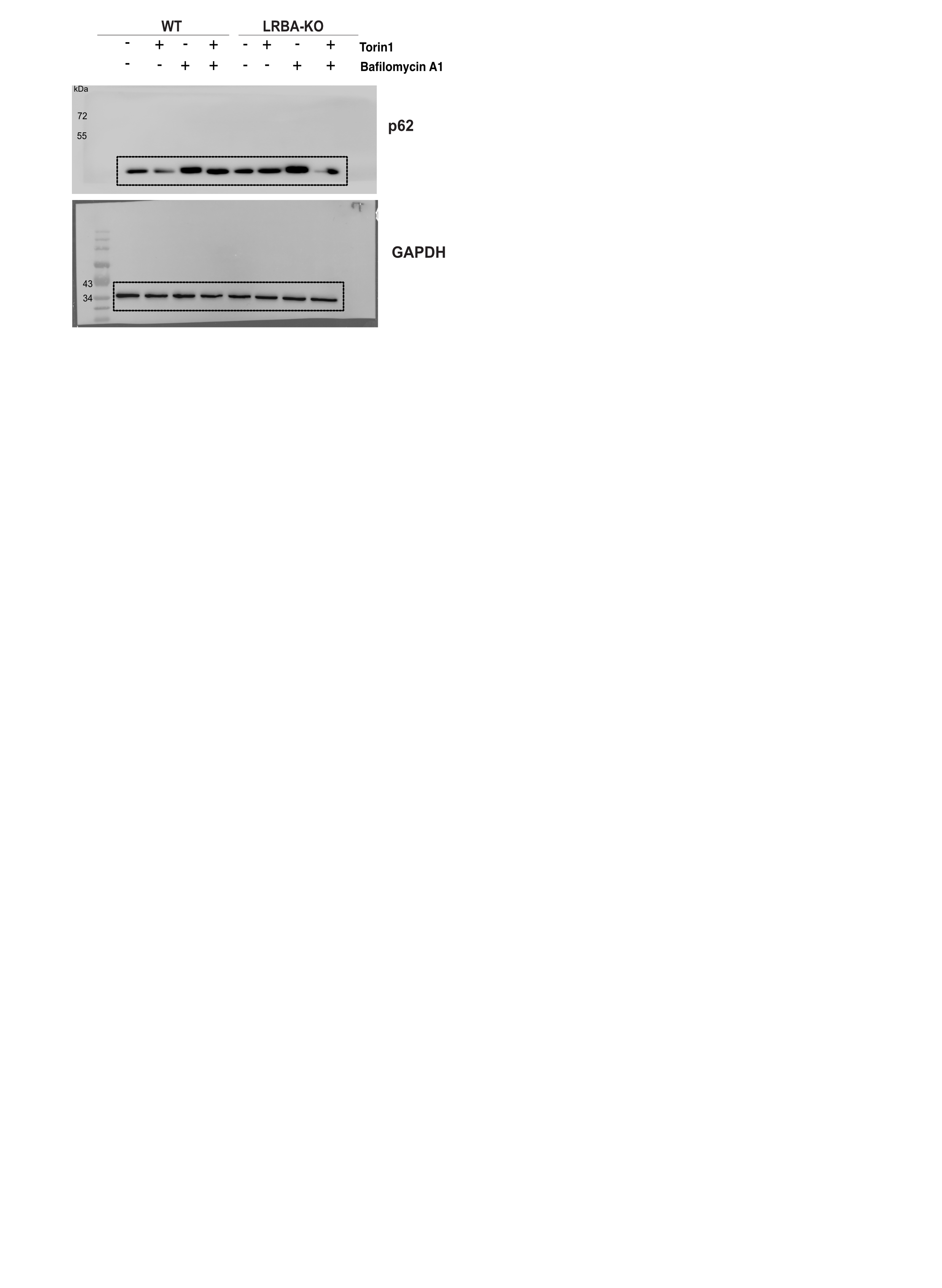

Supplement: Supplementary file 6 — Source data Fig. 3 [file 44319_2025_504_MOESM6_ESM.zip › Figure 3C/p62 Western Blot.tiff]

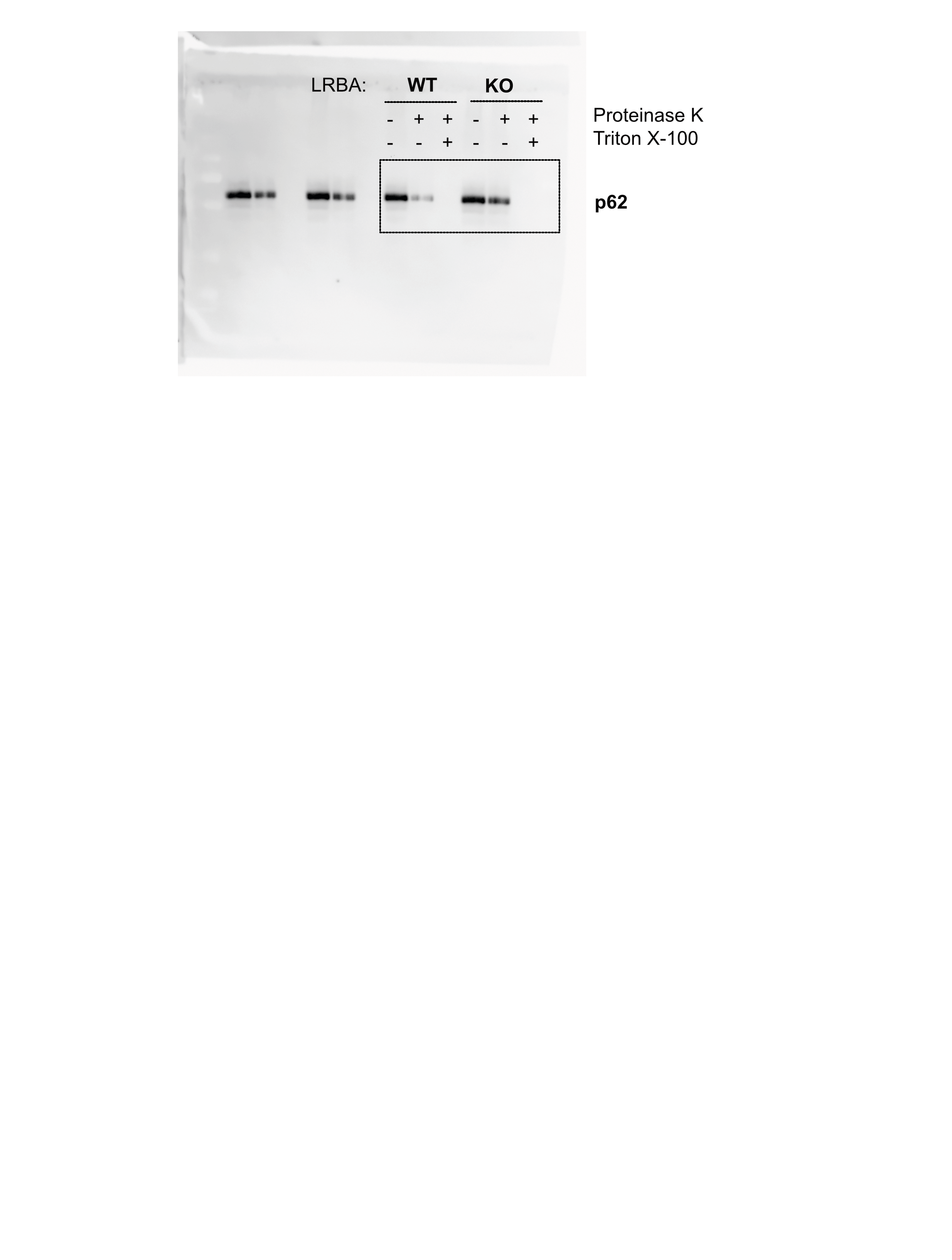

Supplement: Supplementary file 7 — Source data Fig. 4 [file 44319_2025_504_MOESM7_ESM.zip › Figure 4B/Western Blot p62 after Proteinase K treatment.tiff]

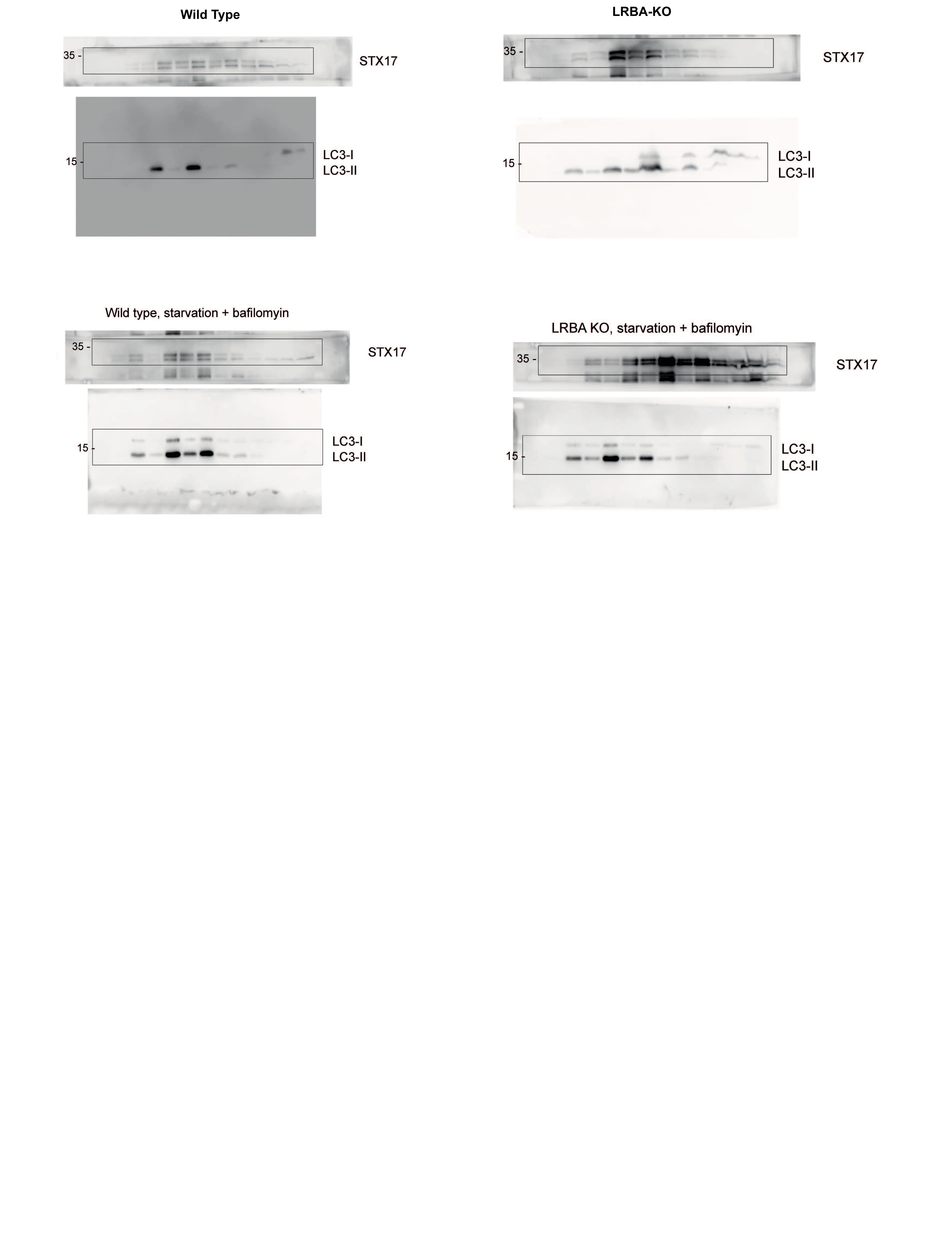

Supplement: Supplementary file 7 — Source data Fig. 4 [file 44319_2025_504_MOESM7_ESM.zip › Figure 4D/Western Blot Optiprep analysis.tiff]

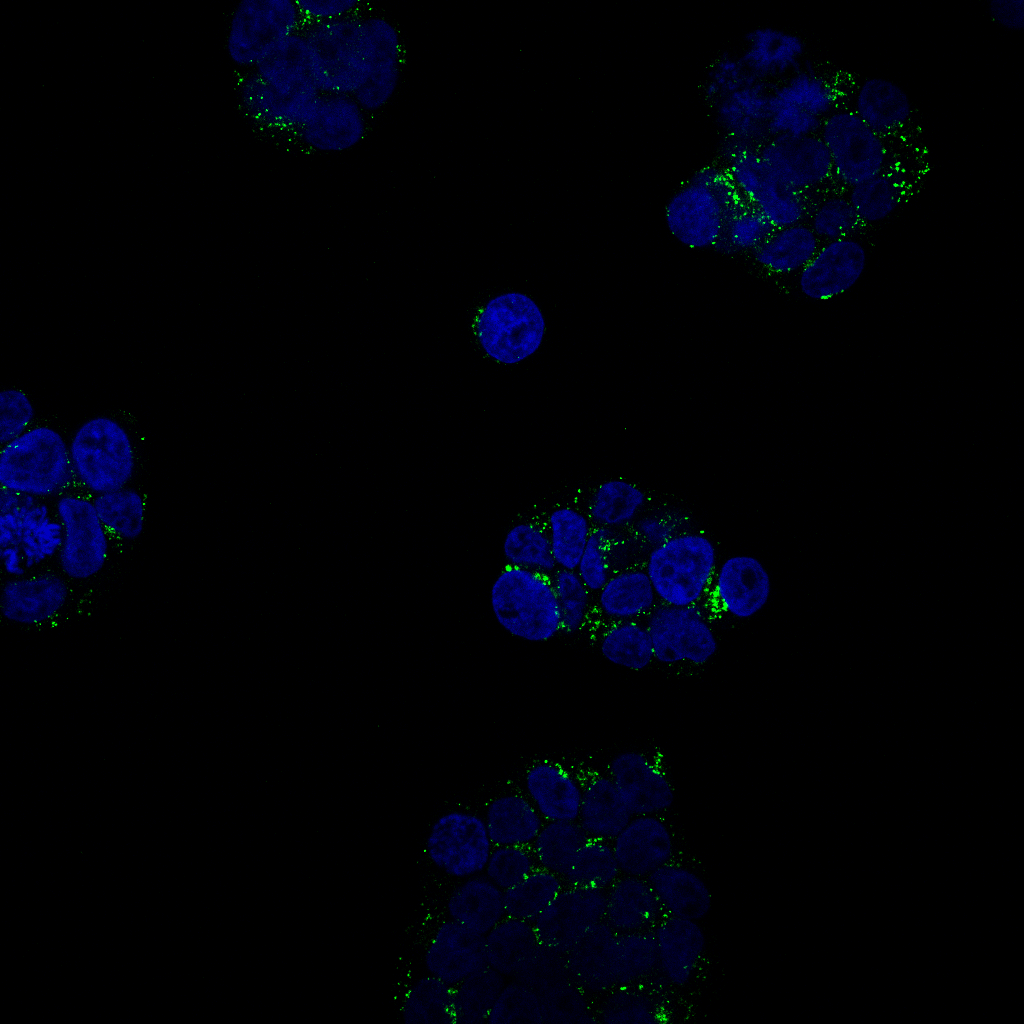

Supplement: Supplementary file 7 — Source data Fig. 4 [file 44319_2025_504_MOESM7_ESM.zip › Figure 4E/LRBA-KO_DAPI+LC3_autophagosome size.tif]

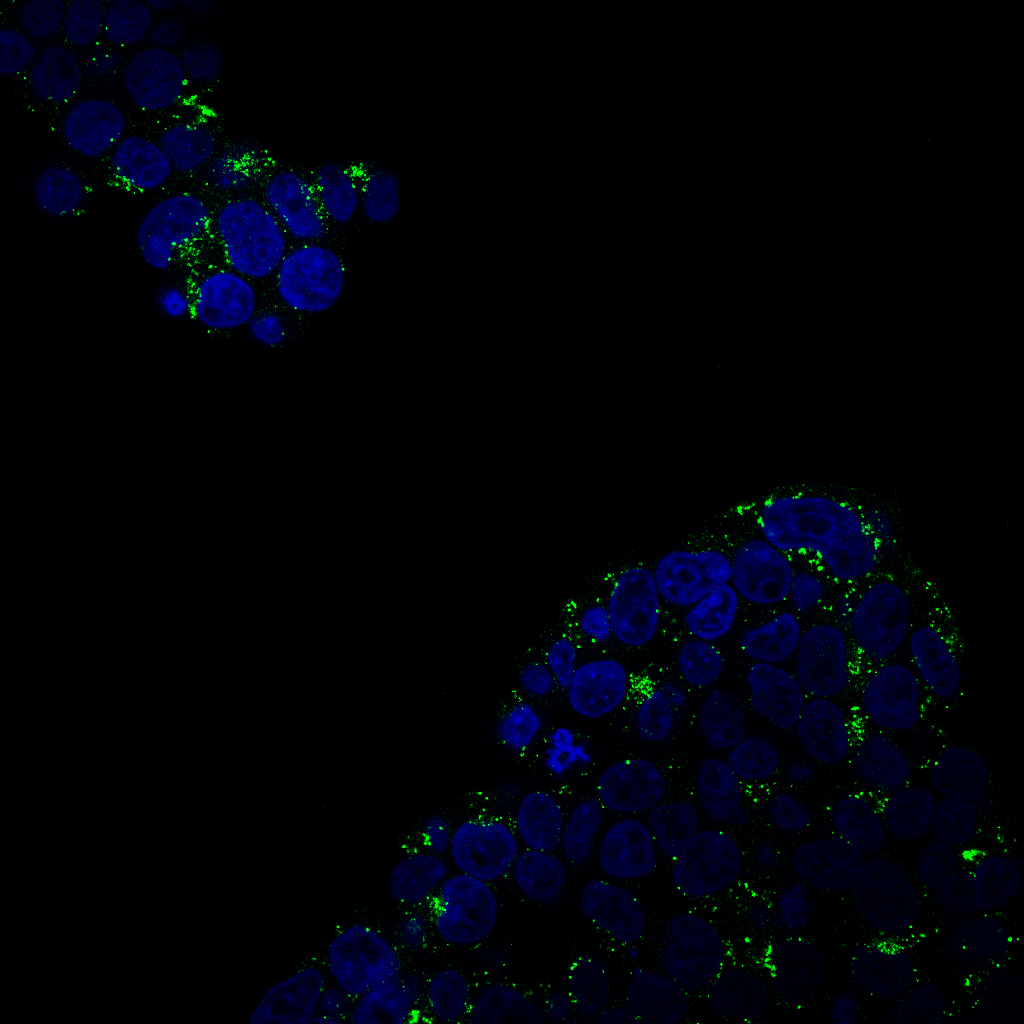

Supplement: Supplementary file 7 — Source data Fig. 4 [file 44319_2025_504_MOESM7_ESM.zip › Figure 4E/WT_DAPI+LC3_autophagosome size.tif]

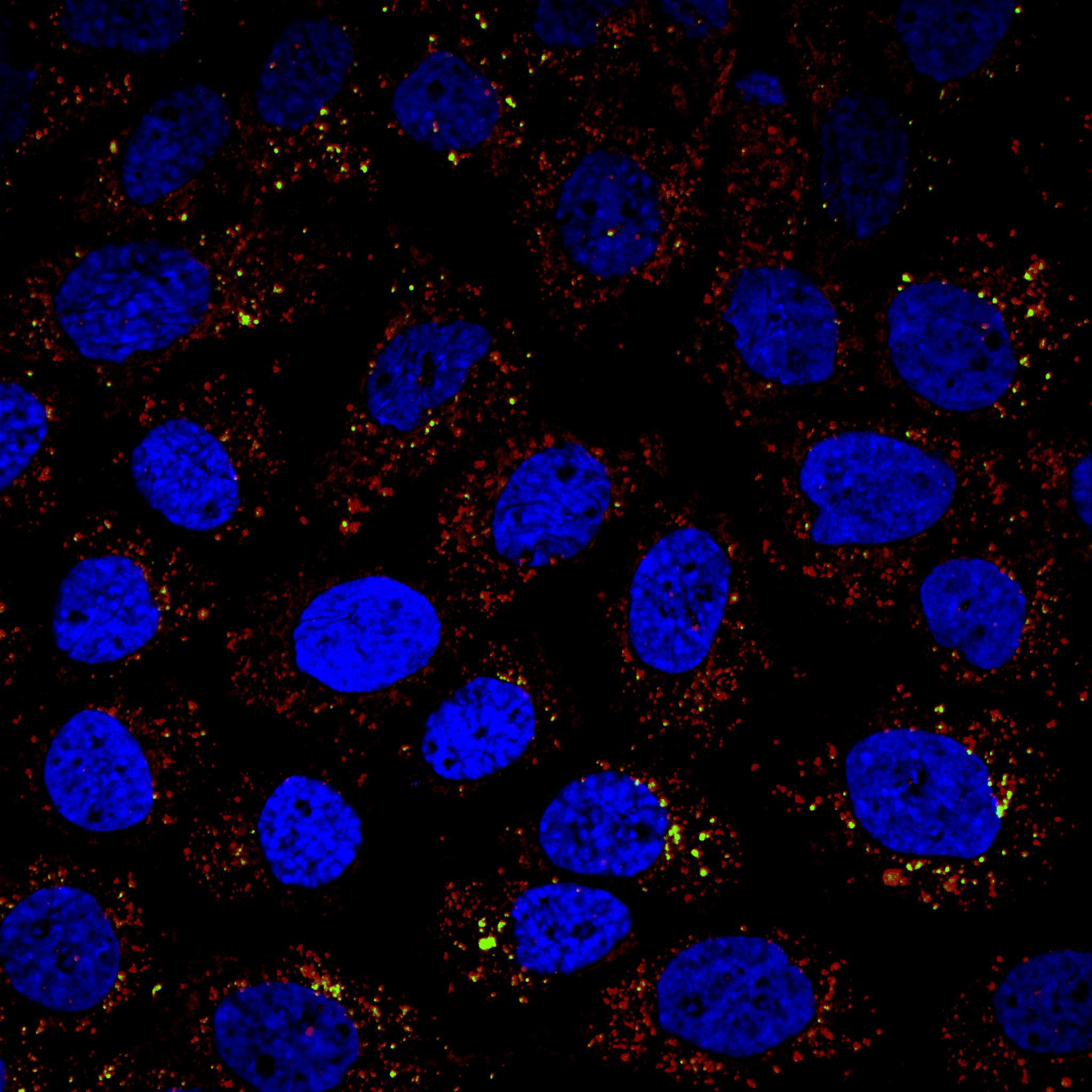

Supplement: Supplementary file 7 — Source data Fig. 4 [file 44319_2025_504_MOESM7_ESM.zip › Figure 4G/HaCat LRBA-KO_p62+LC3+DAPI.tif]

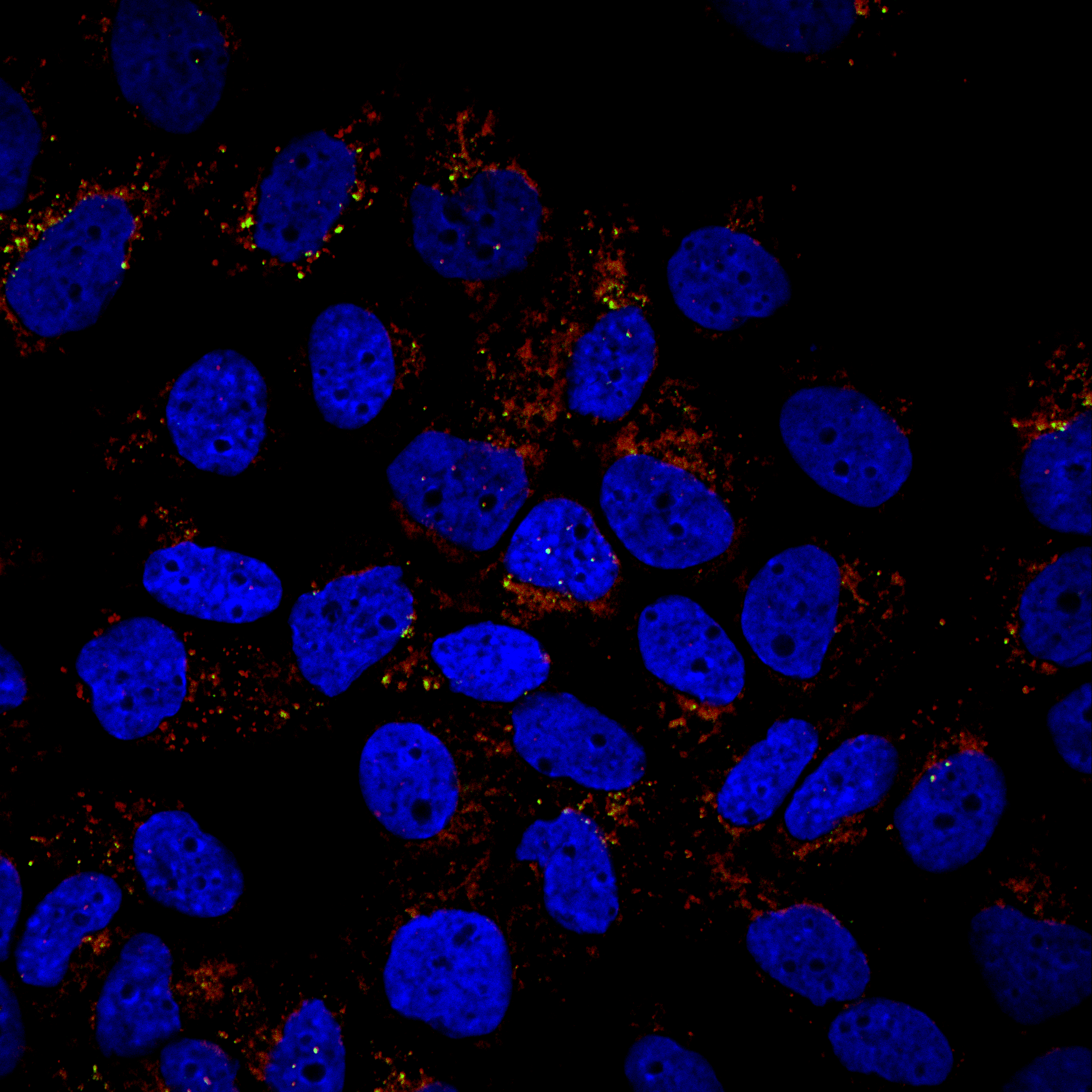

Supplement: Supplementary file 7 — Source data Fig. 4 [file 44319_2025_504_MOESM7_ESM.zip › Figure 4G/HaCat WT_p62+LC3+DAPI.tif]

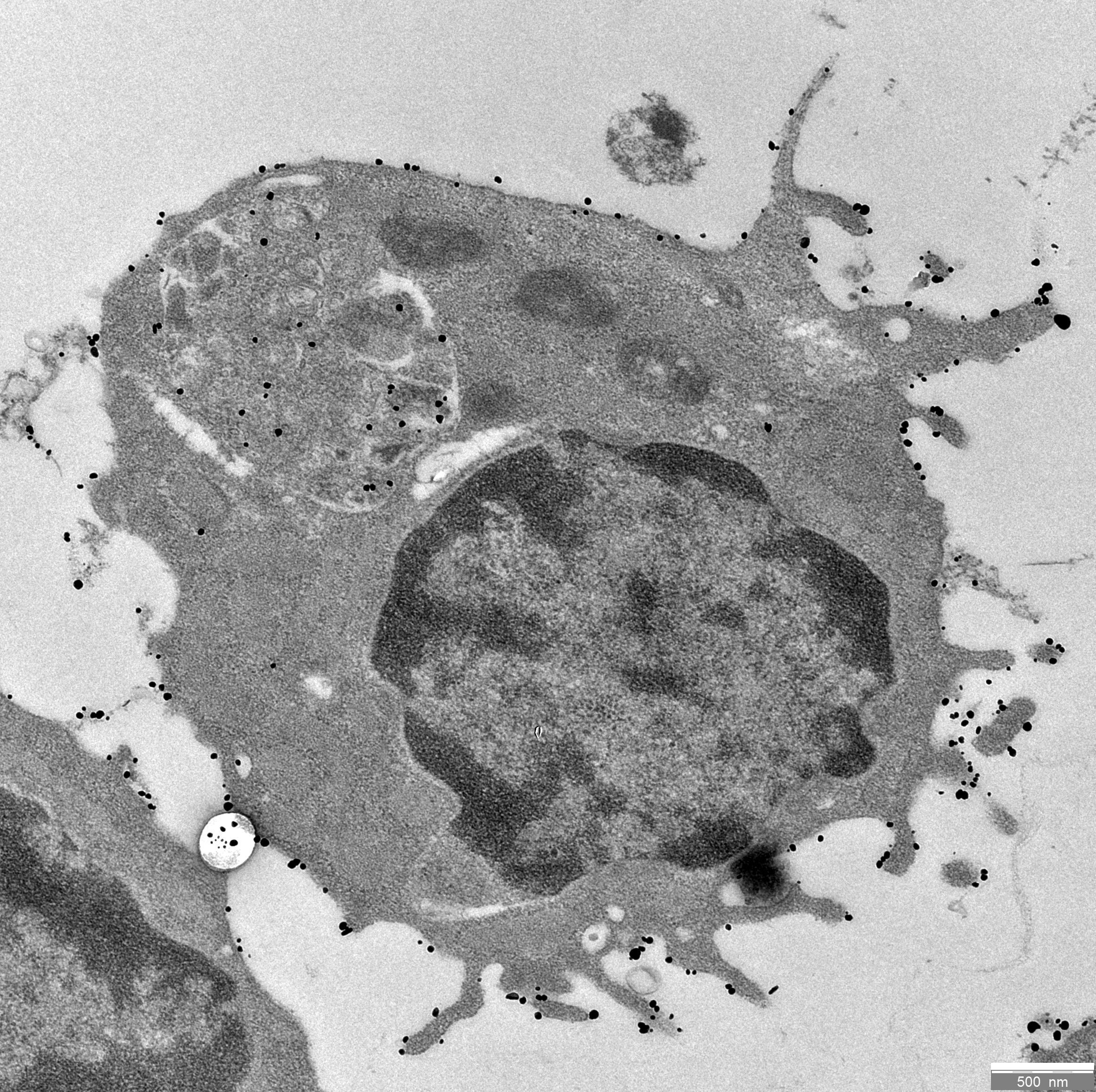

Supplement: Supplementary file 7 — Source data Fig. 4 [file 44319_2025_504_MOESM7_ESM.zip › Figure 4J/Lrba-KO_mLC3_LPS.TIF]

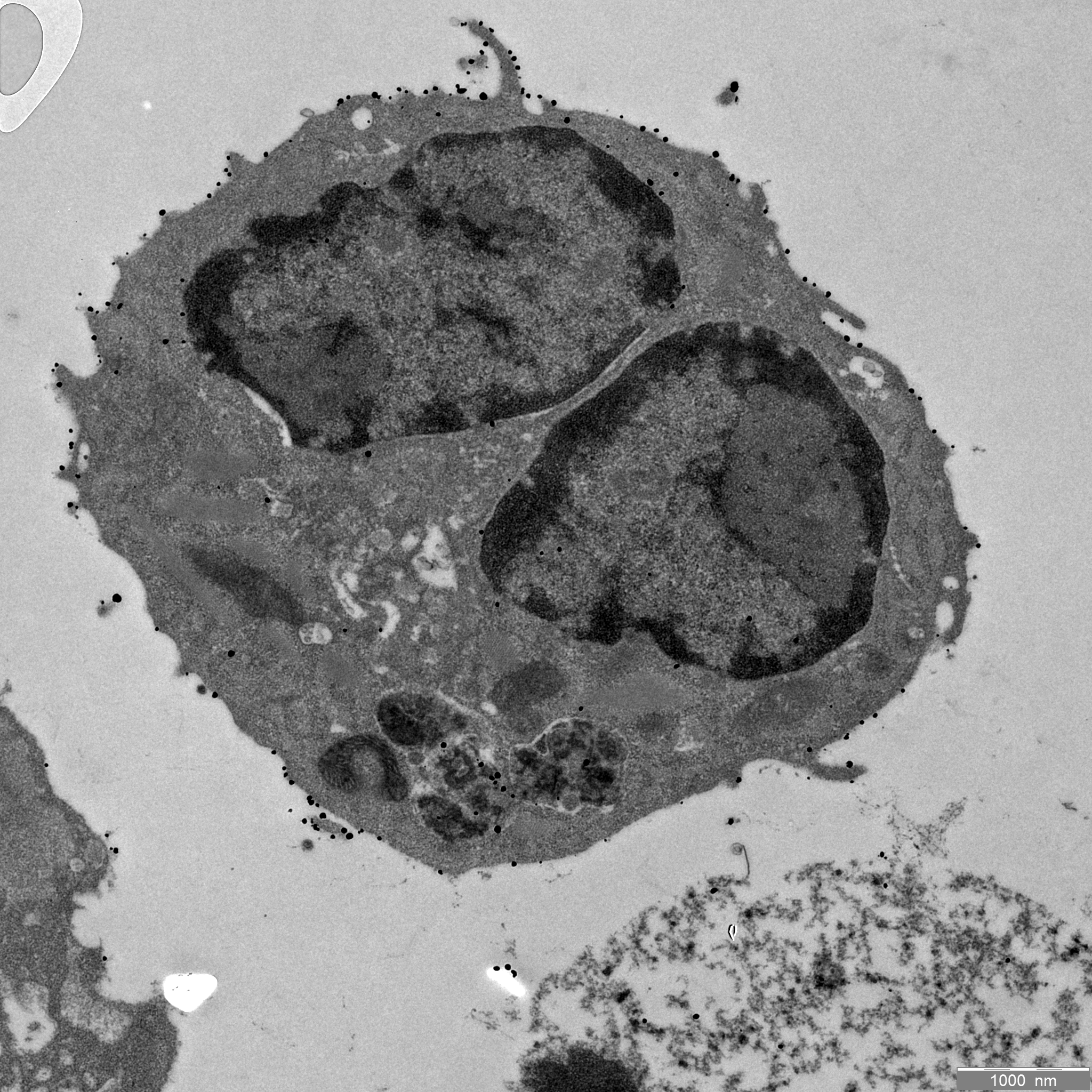

Supplement: Supplementary file 7 — Source data Fig. 4 [file 44319_2025_504_MOESM7_ESM.zip › Figure 4J/Lrba-KO_mLC3_LPS+Bafilomycin A1_1000nm.TIF]

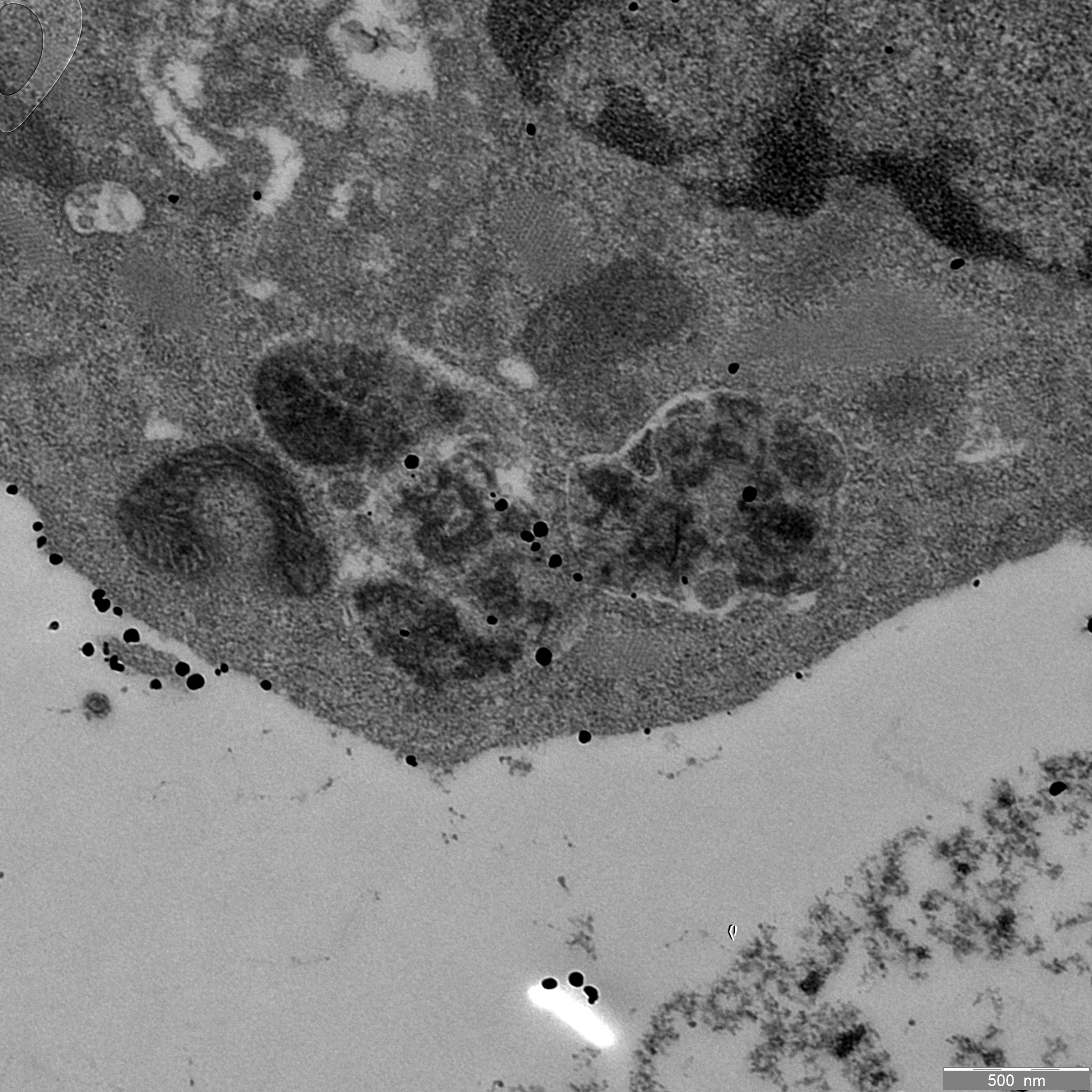

Supplement: Supplementary file 7 — Source data Fig. 4 [file 44319_2025_504_MOESM7_ESM.zip › Figure 4J/Lrba-KO_mLC3_LPS+Bafilomycin A1_500nm.TIF]

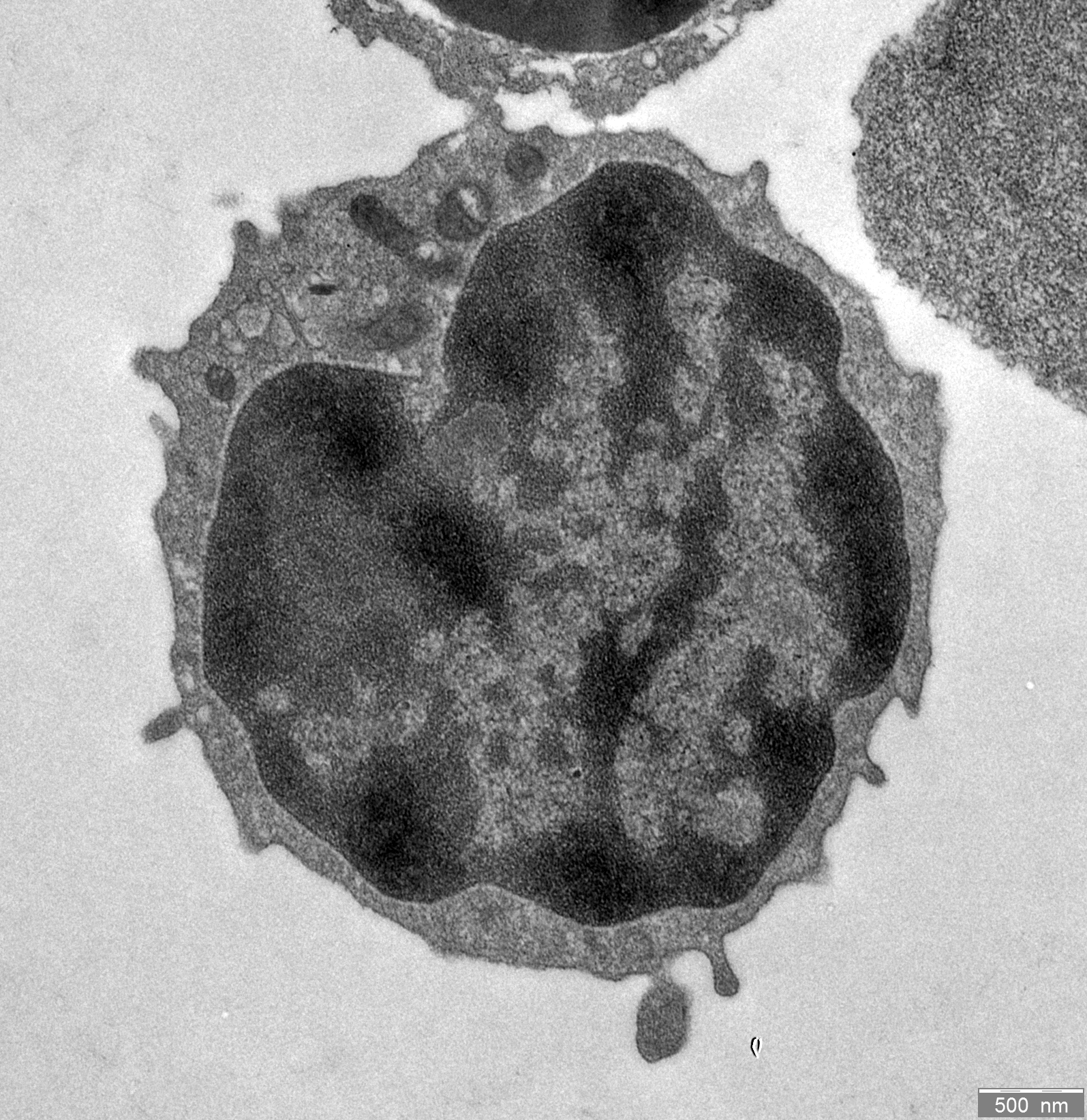

Supplement: Supplementary file 7 — Source data Fig. 4 [file 44319_2025_504_MOESM7_ESM.zip › Figure 4J/Wt_mLC3_LPS.TIF]

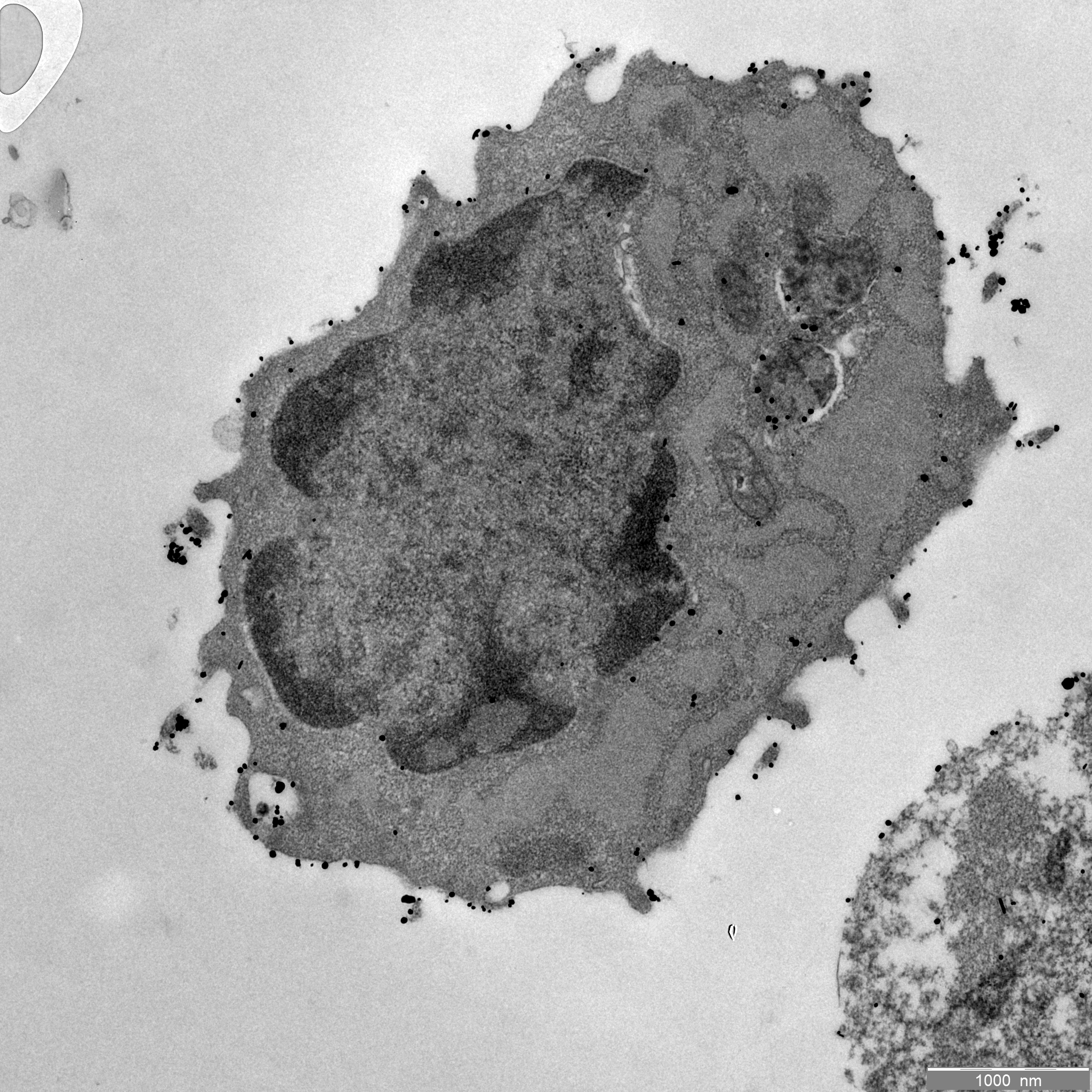

Supplement: Supplementary file 7 — Source data Fig. 4 [file 44319_2025_504_MOESM7_ESM.zip › Figure 4J/Wt_mLC3_LPS+Bafilomycin A1_1000nm.TIF]

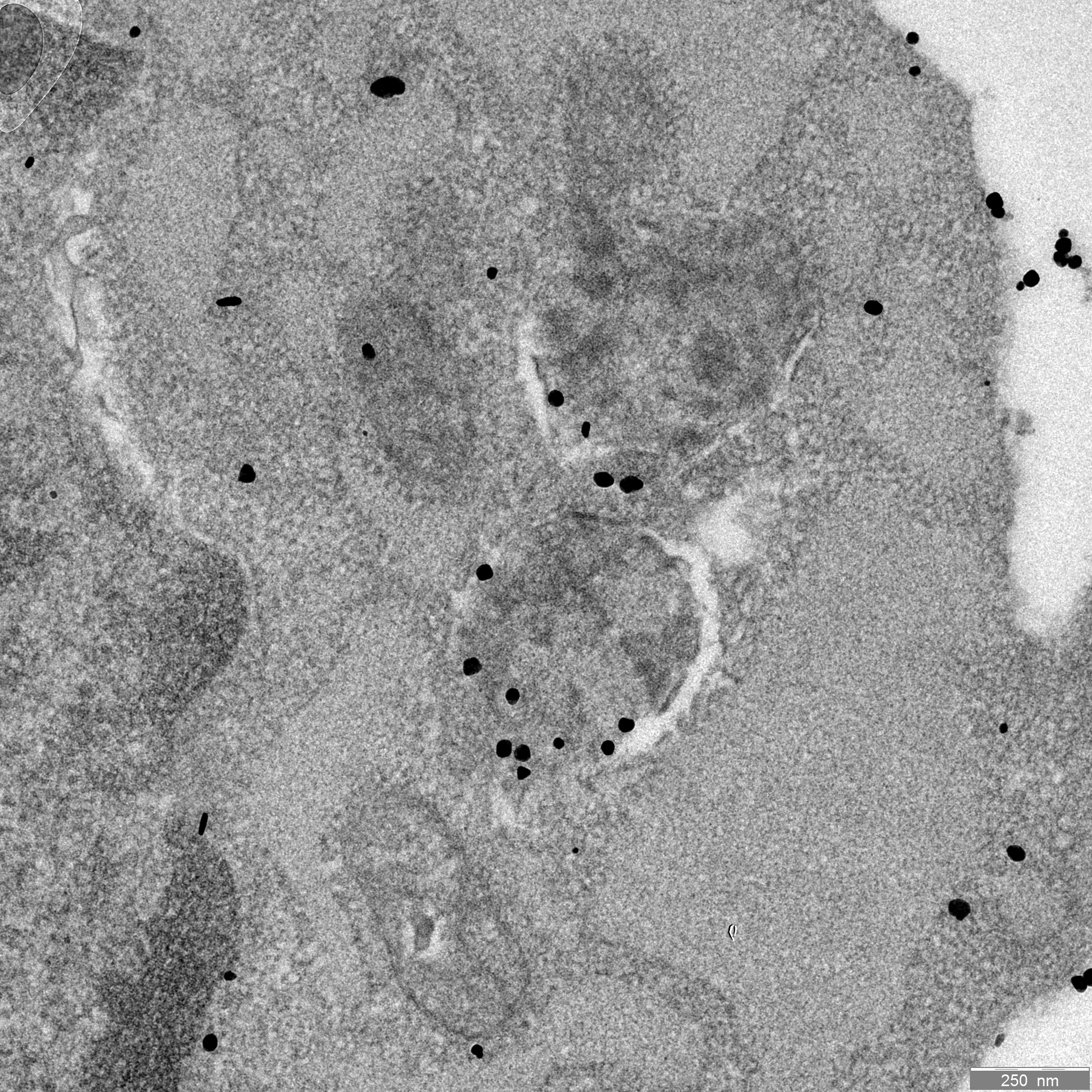

Supplement: Supplementary file 7 — Source data Fig. 4 [file 44319_2025_504_MOESM7_ESM.zip › Figure 4J/Wt_mLC3_LPS+Bafilomycin A1_250nm.TIF]

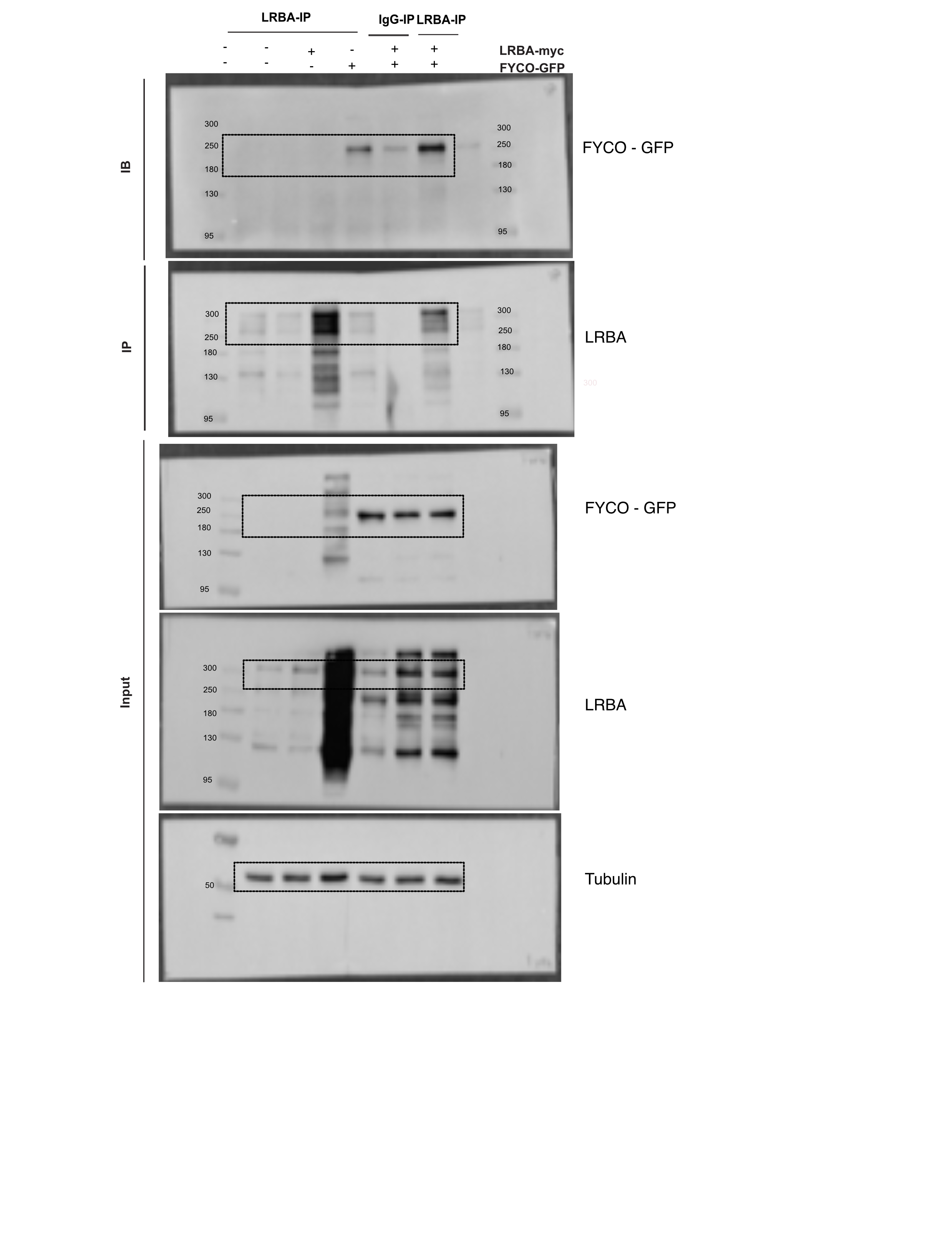

Supplement: Supplementary file 8 — Source data Fig. 5 [file 44319_2025_504_MOESM8_ESM.zip › Figure 5A/Co-IP LRBA and FYCO1.tiff]

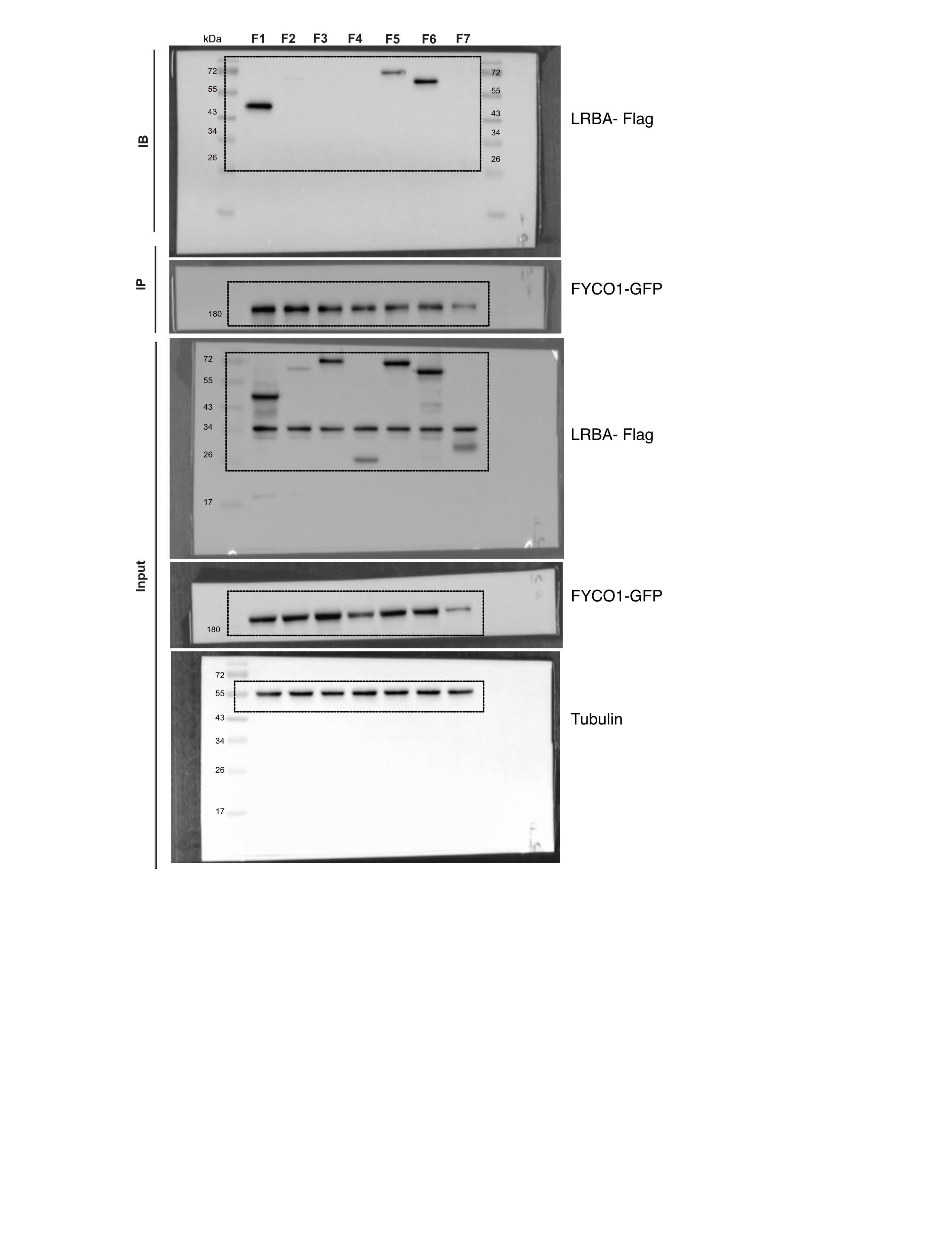

Supplement: Supplementary file 8 — Source data Fig. 5 [file 44319_2025_504_MOESM8_ESM.zip › Figure 5B/Co-IP FYCO1 and LRBA fragments.tiff]

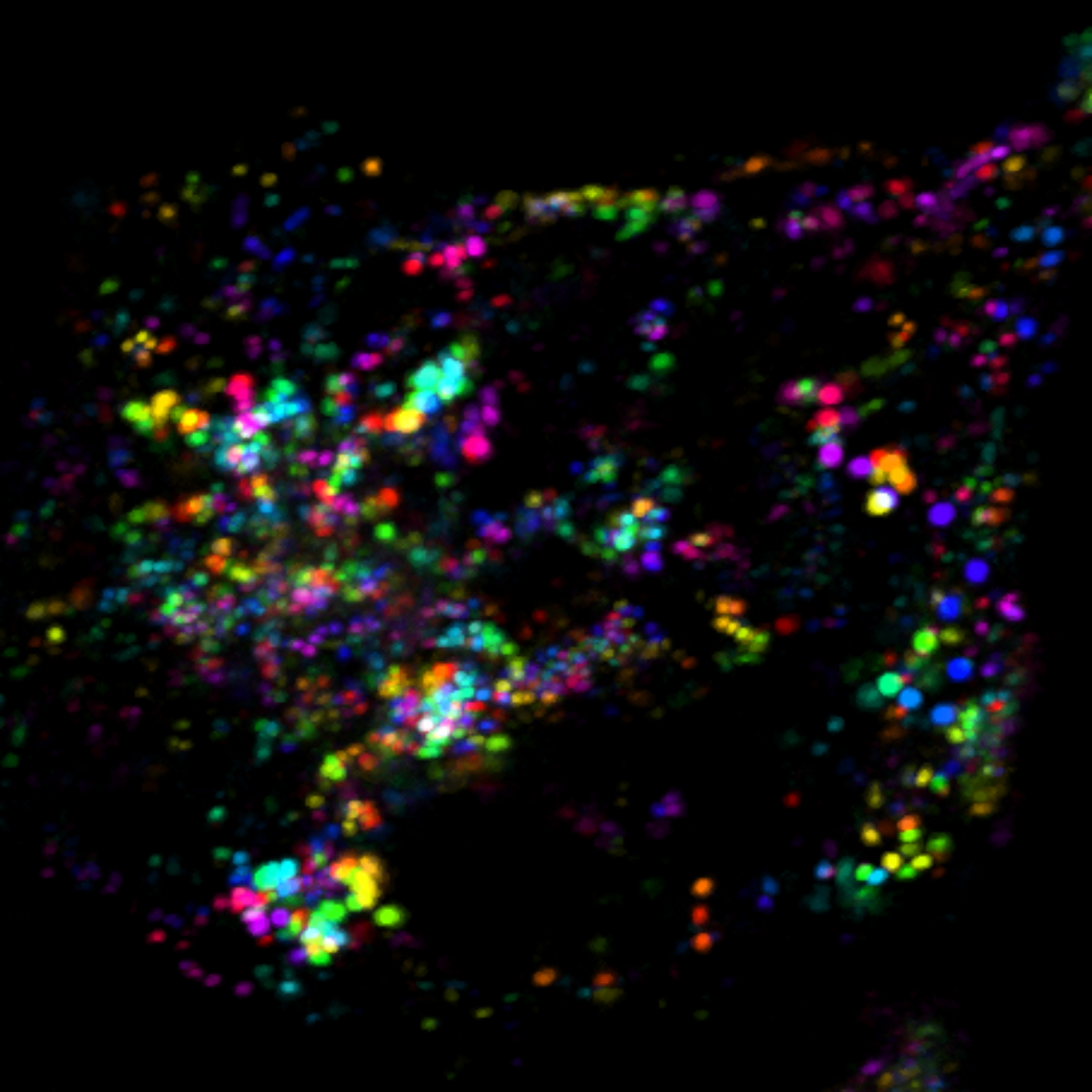

Supplement: Supplementary file 8 — Source data Fig. 5 [file 44319_2025_504_MOESM8_ESM.zip › Figure 5D/shControl_autophagosome mobility.tiff]

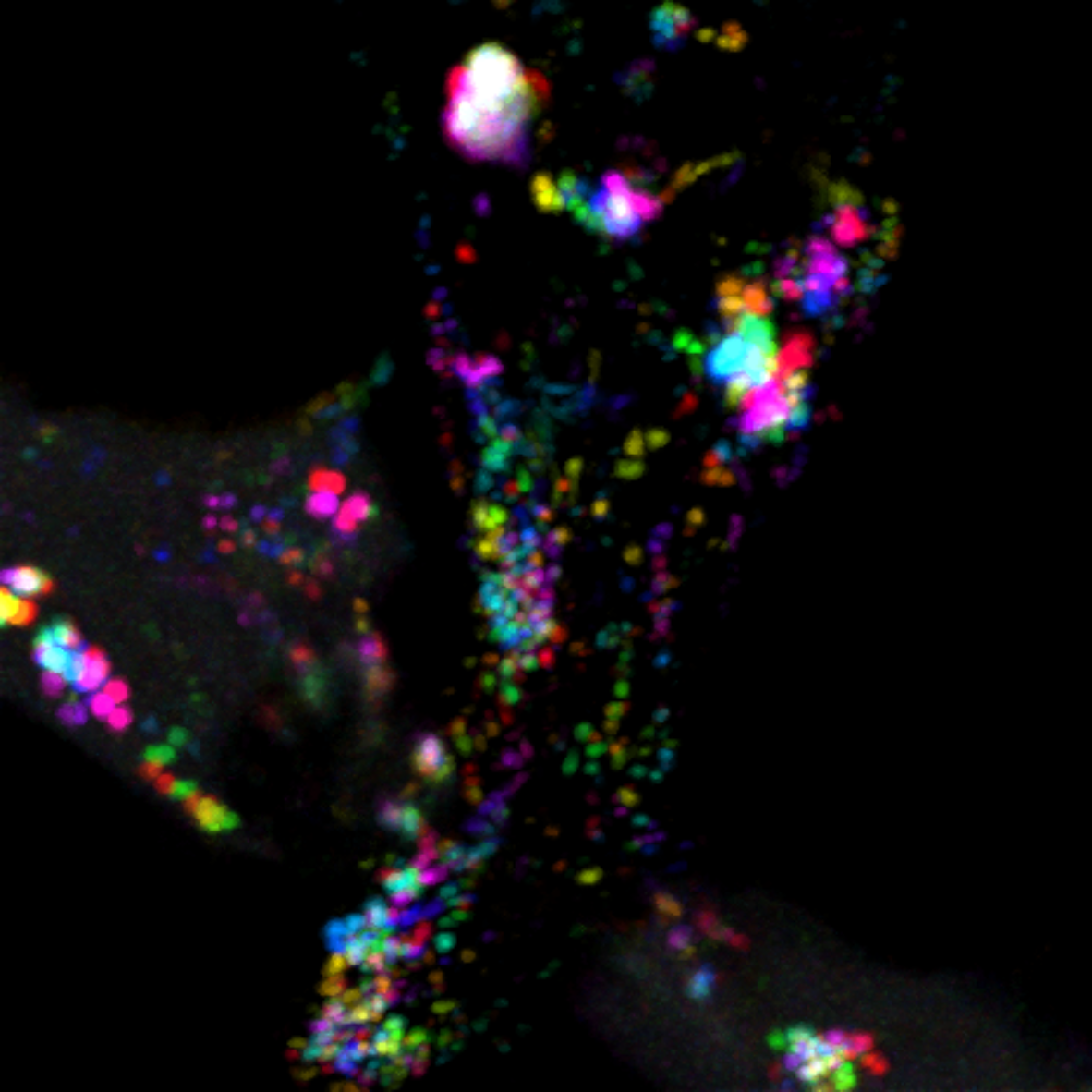

Supplement: Supplementary file 8 — Source data Fig. 5 [file 44319_2025_504_MOESM8_ESM.zip › Figure 5D/shLRBA_autophagosome mobility.tiff]

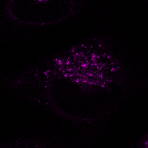

Supplement: Supplementary file 8 — Source data Fig. 5 [file 44319_2025_504_MOESM8_ESM.zip › Figure 5G/HeLa shControl_LysoTracker Red.tif]

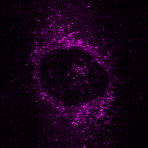

Supplement: Supplementary file 8 — Source data Fig. 5 [file 44319_2025_504_MOESM8_ESM.zip › Figure 5G/HeLa shLRBA_LysoTracker Red.tif]

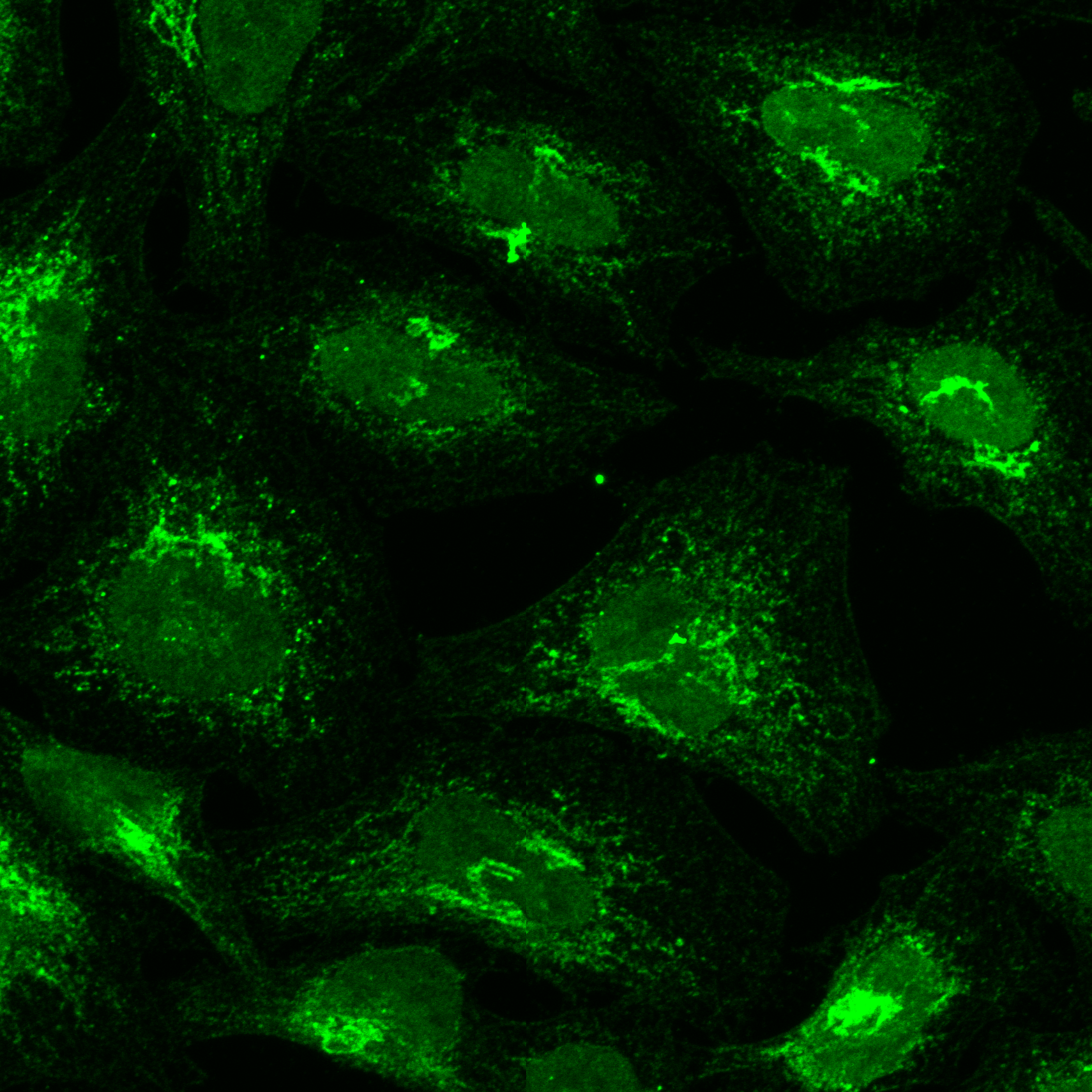

Supplement: Supplementary file 8 — Source data Fig. 5 [file 44319_2025_504_MOESM8_ESM.zip › Figure 5H/HeLa_shControl_anti-TFEB.tif]

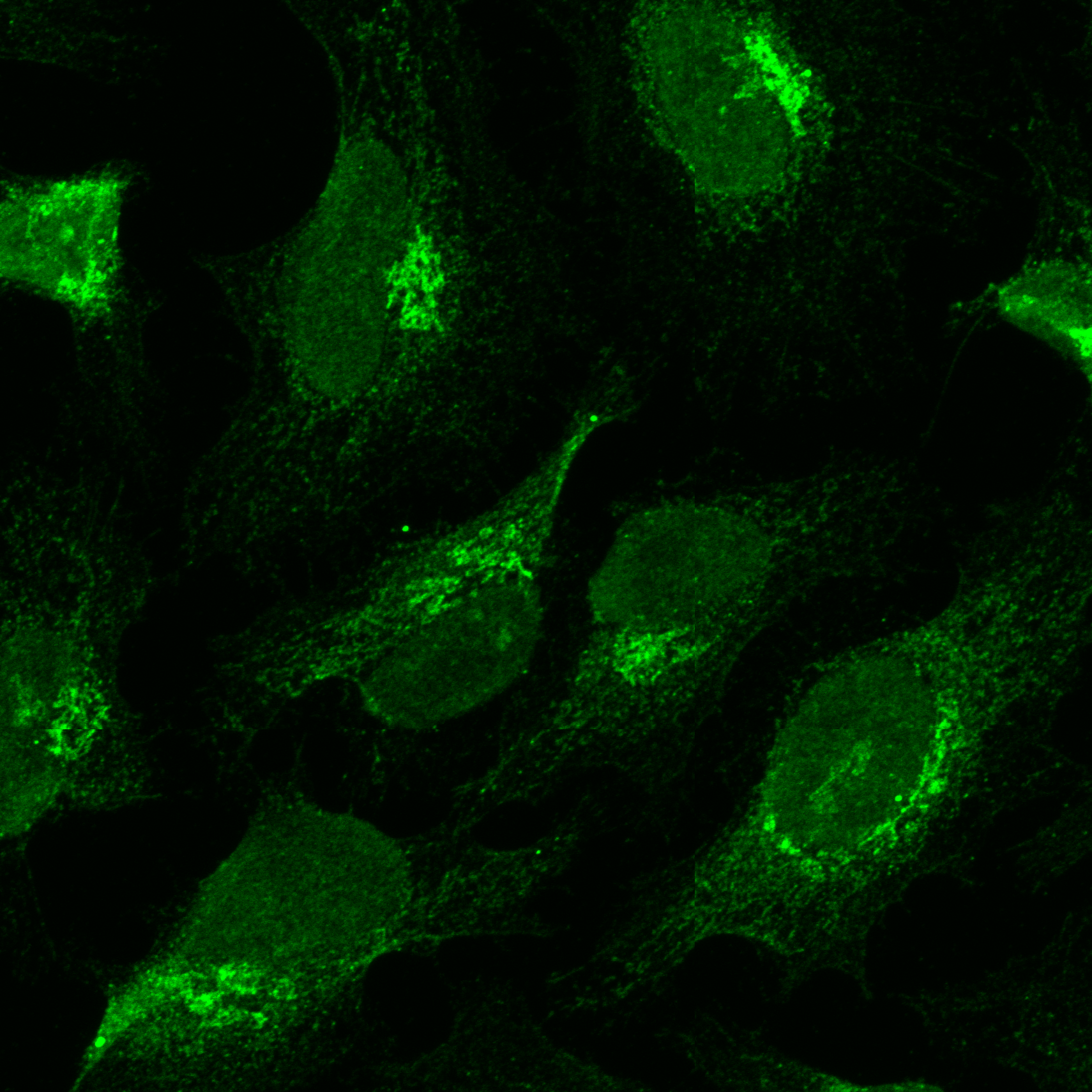

Supplement: Supplementary file 8 — Source data Fig. 5 [file 44319_2025_504_MOESM8_ESM.zip › Figure 5H/HeLa_shLRBA_anti-TFEB.tif]

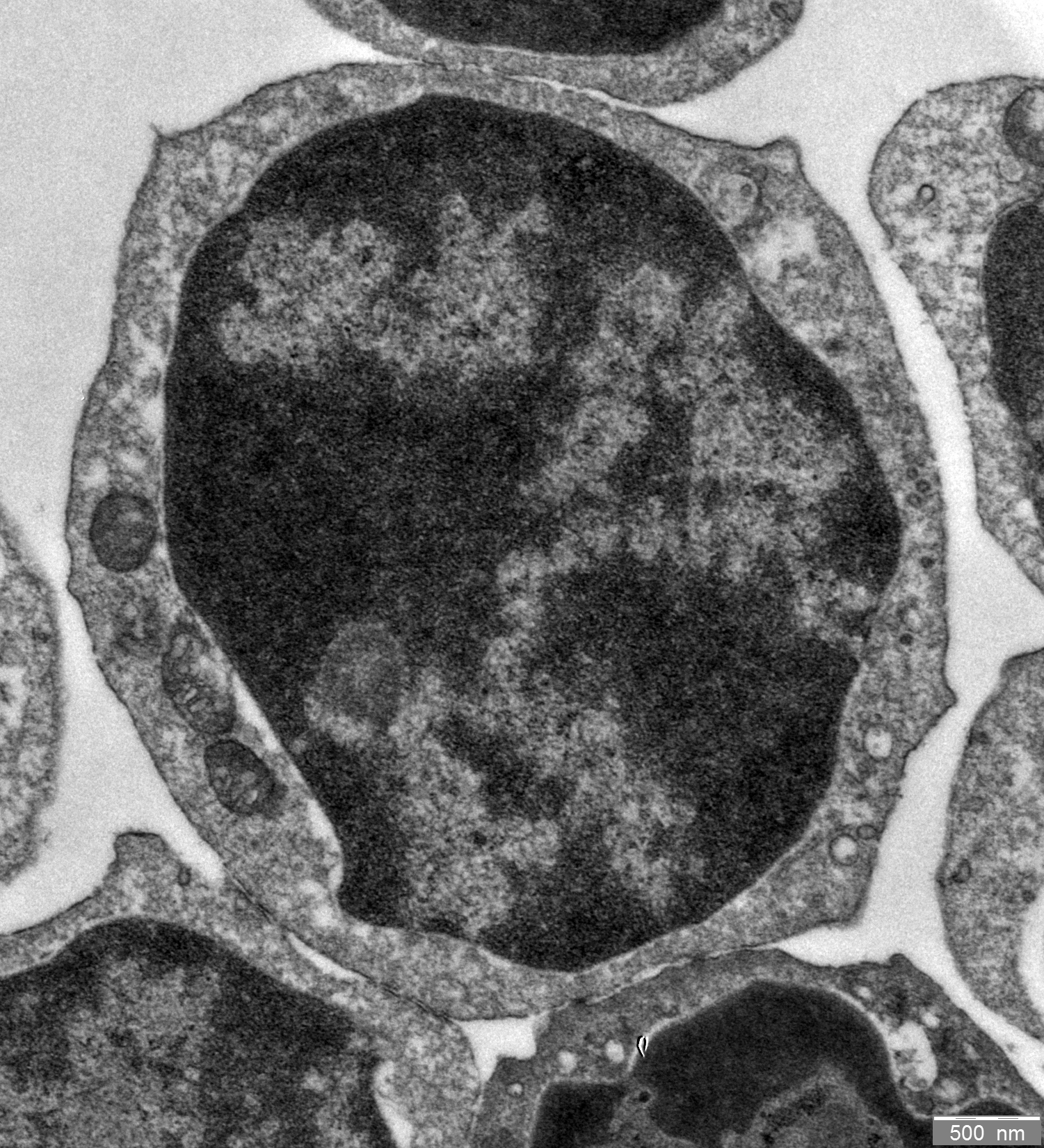

Supplement: Supplementary file 8 — Source data Fig. 5 [file 44319_2025_504_MOESM8_ESM.zip › Figure 5I/Lrba-KO_mLamp1_Basal_500nm.TIF]

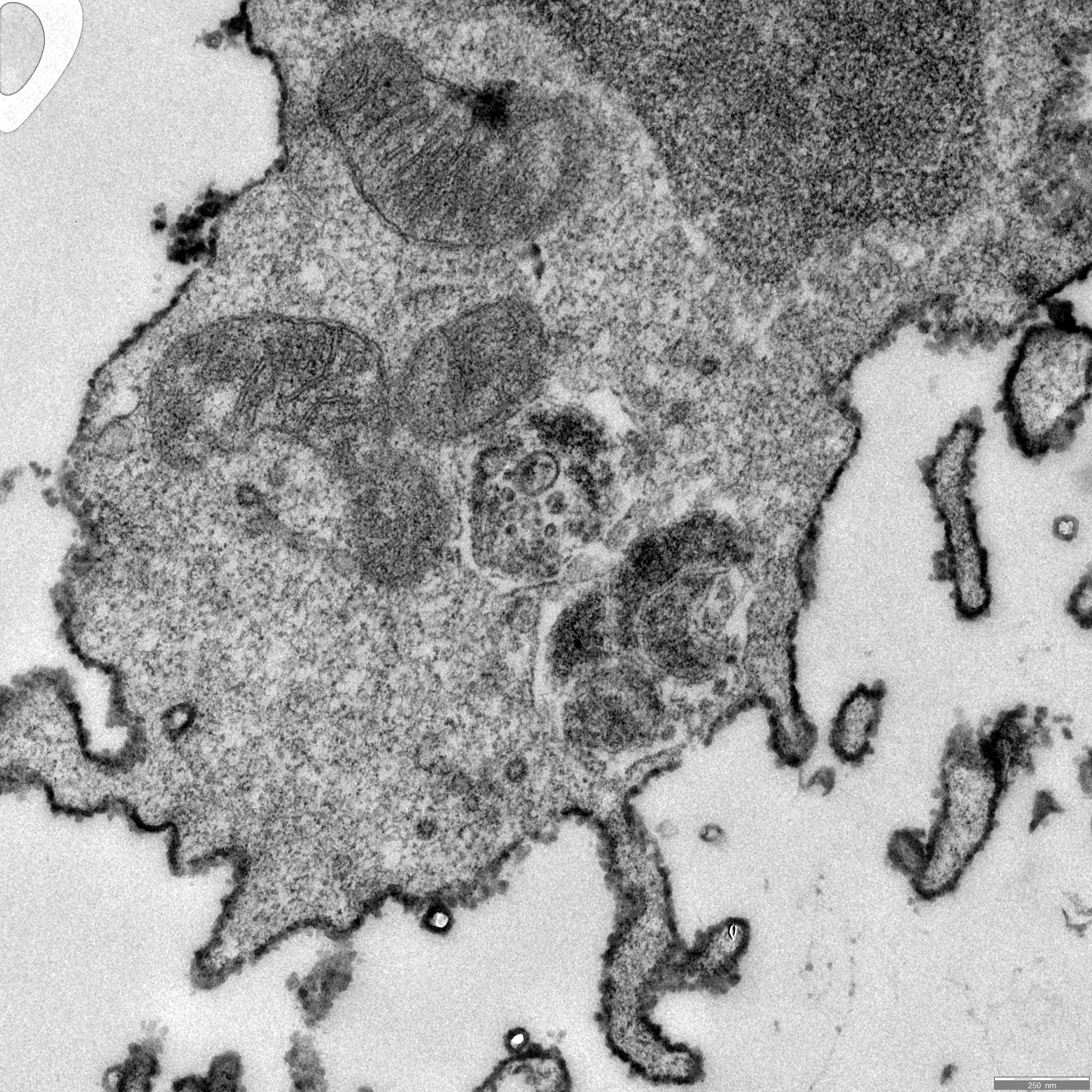

Supplement: Supplementary file 8 — Source data Fig. 5 [file 44319_2025_504_MOESM8_ESM.zip › Figure 5I/Lrba-KO_mLamp1_LPS_250nm.TIF]

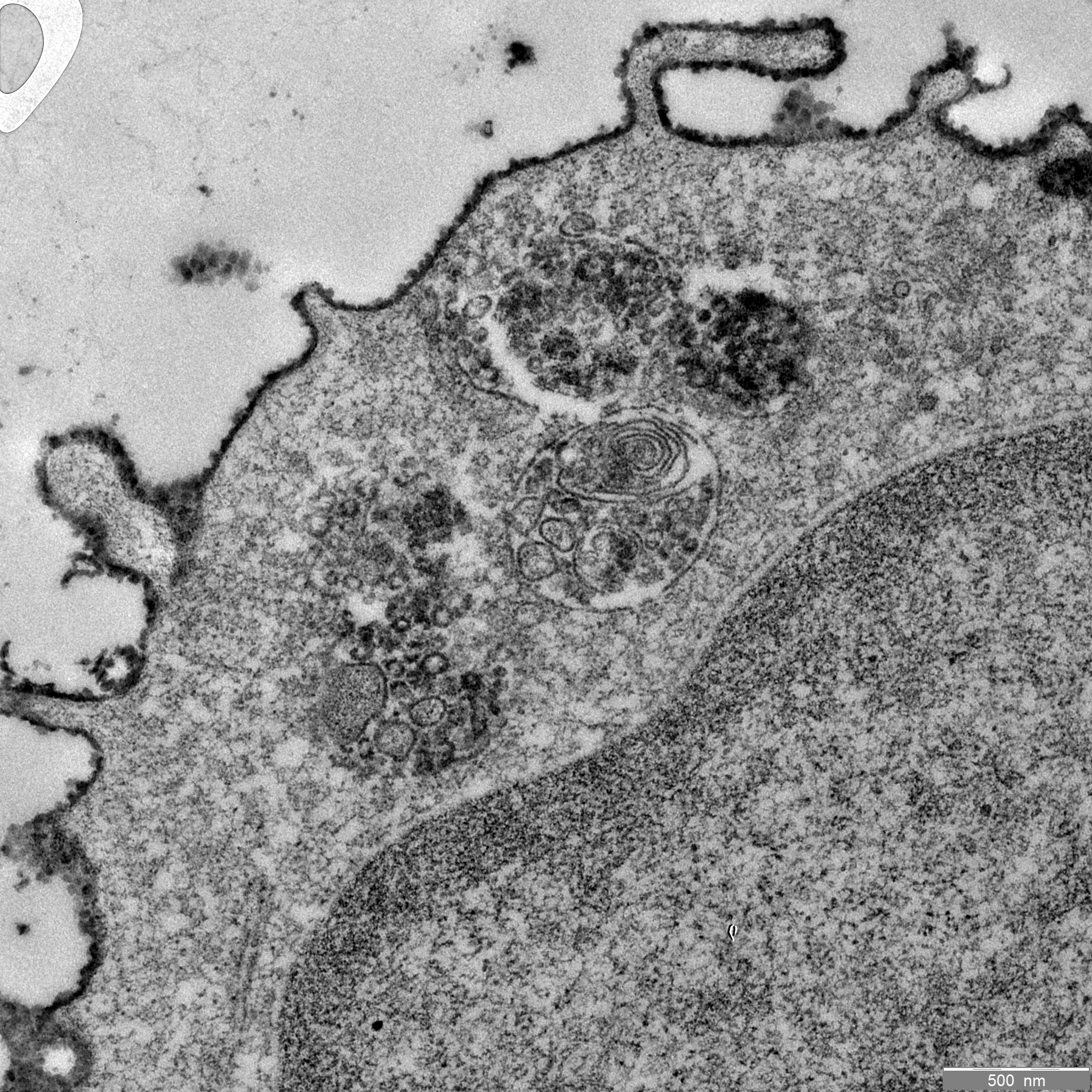

Supplement: Supplementary file 8 — Source data Fig. 5 [file 44319_2025_504_MOESM8_ESM.zip › Figure 5I/Lrba-KO_mLamp1_LPS+Bafilomycin_500nm.TIF]

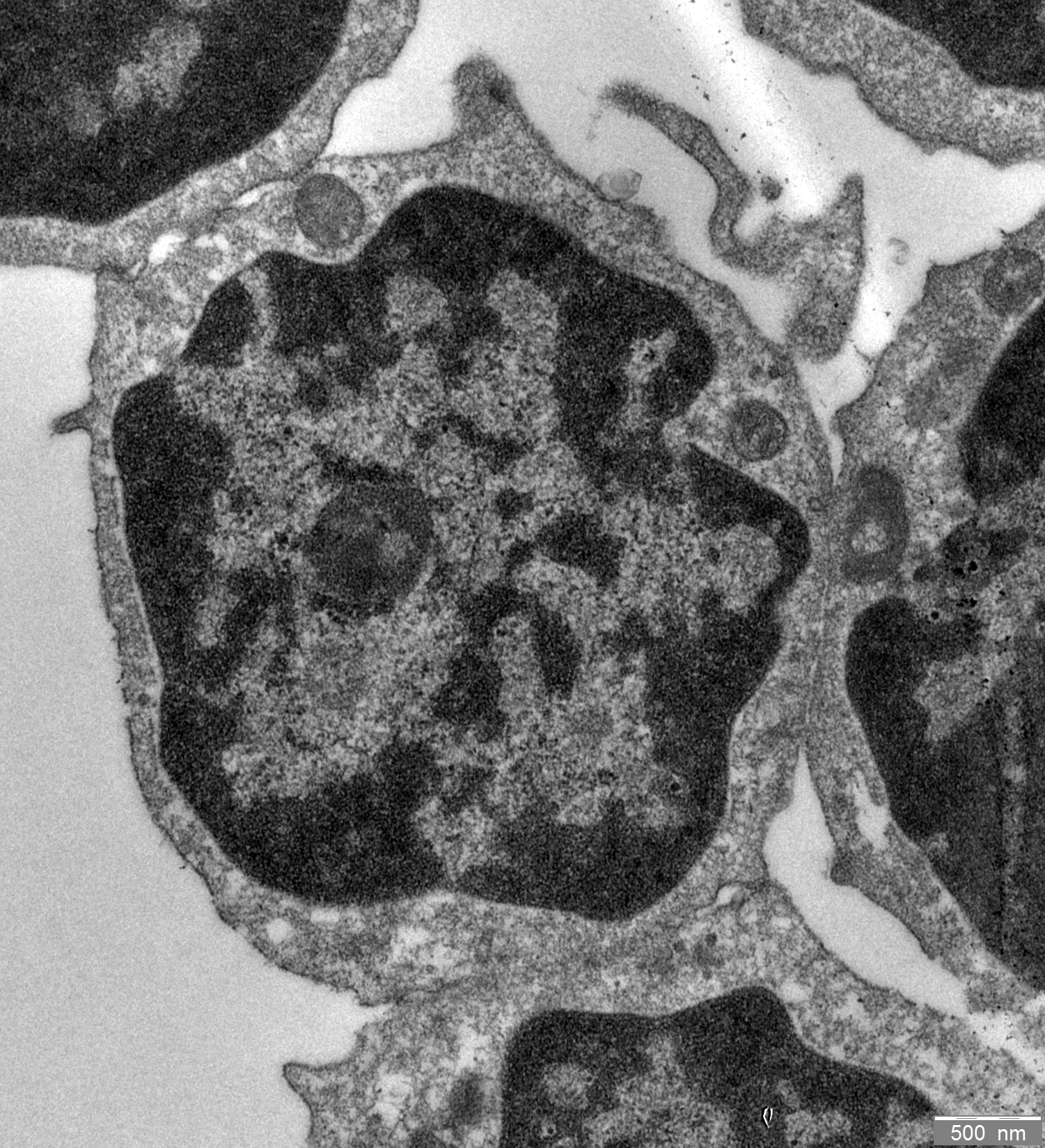

Supplement: Supplementary file 8 — Source data Fig. 5 [file 44319_2025_504_MOESM8_ESM.zip › Figure 5I/Wt_mLamp1_Basal_500nm.TIF]

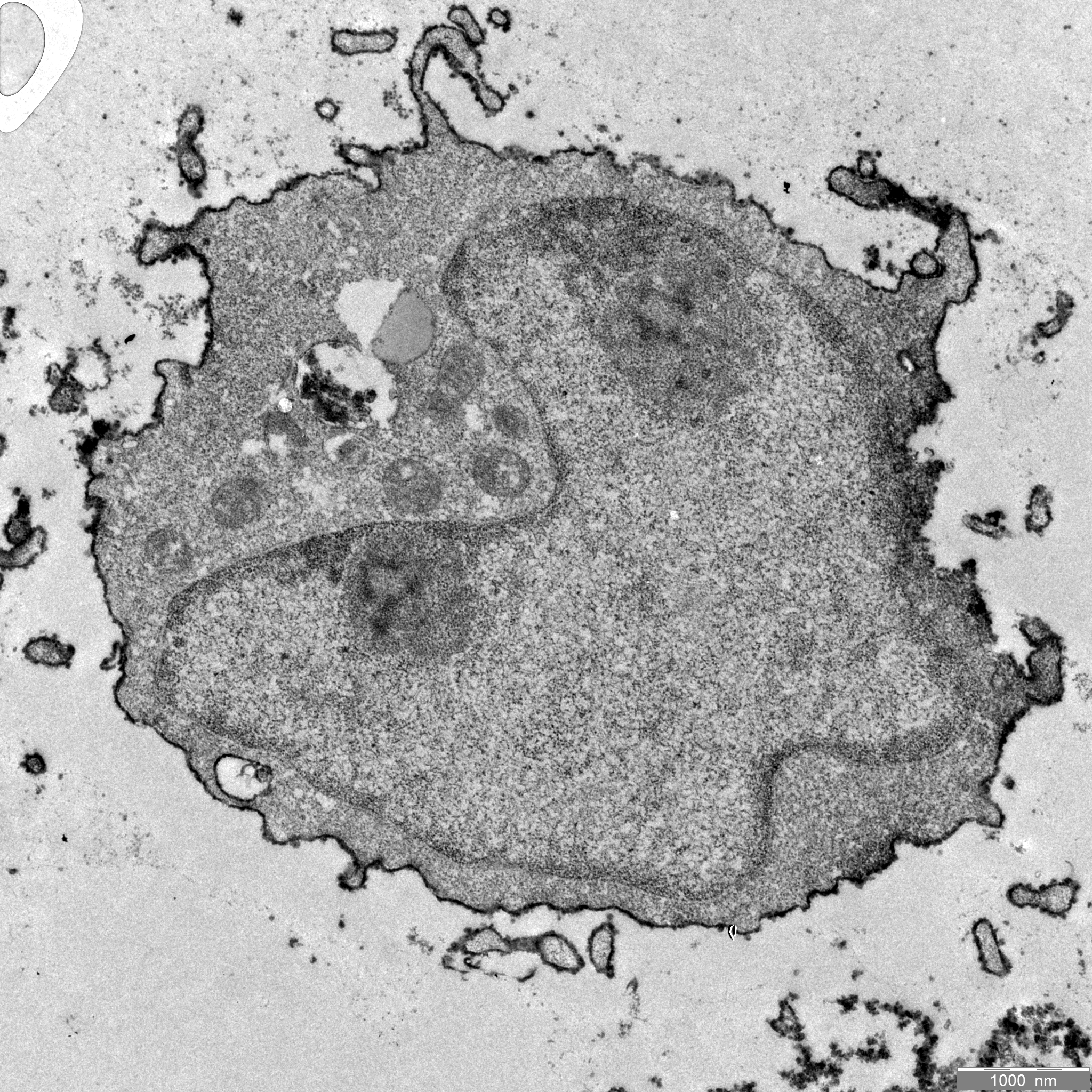

Supplement: Supplementary file 8 — Source data Fig. 5 [file 44319_2025_504_MOESM8_ESM.zip › Figure 5I/Wt_mLamp1_LPS_1000nm.TIF]

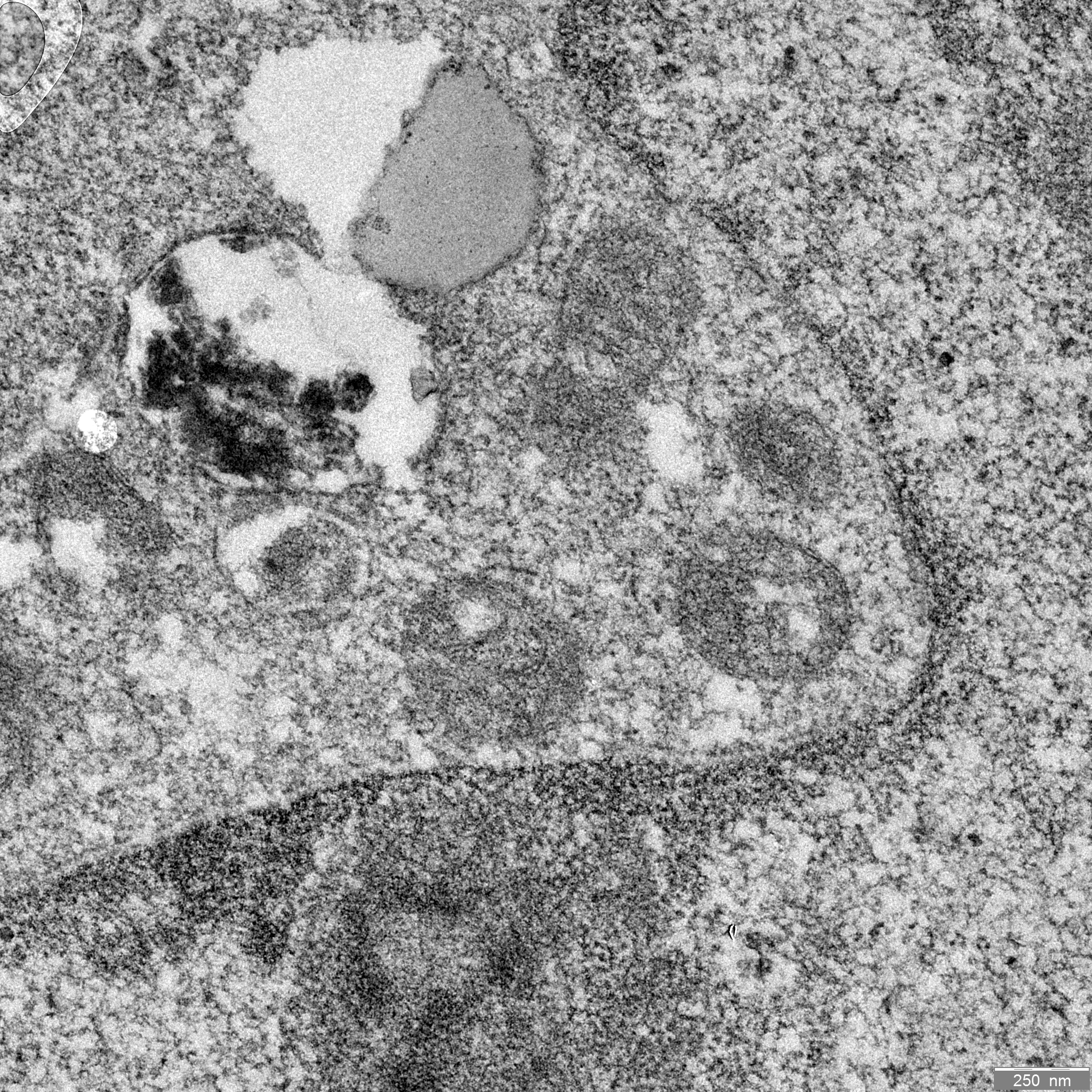

Supplement: Supplementary file 8 — Source data Fig. 5 [file 44319_2025_504_MOESM8_ESM.zip › Figure 5I/Wt_mLamp1_LPS_250nm.TIF]

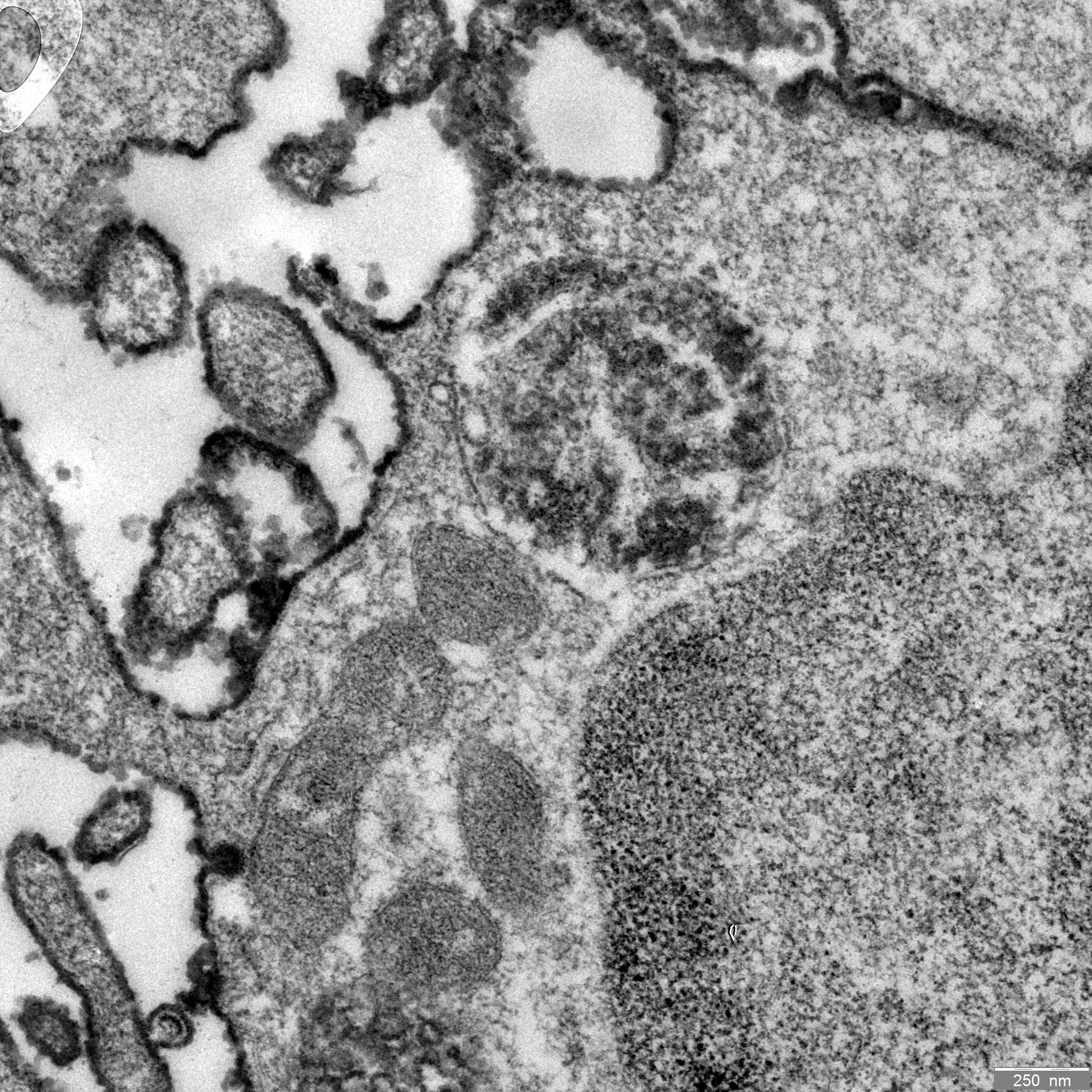

Supplement: Supplementary file 8 — Source data Fig. 5 [file 44319_2025_504_MOESM8_ESM.zip › Figure 5I/Wt_mLamp1_LPS+Bafilomycin A1_250nm.TIF]

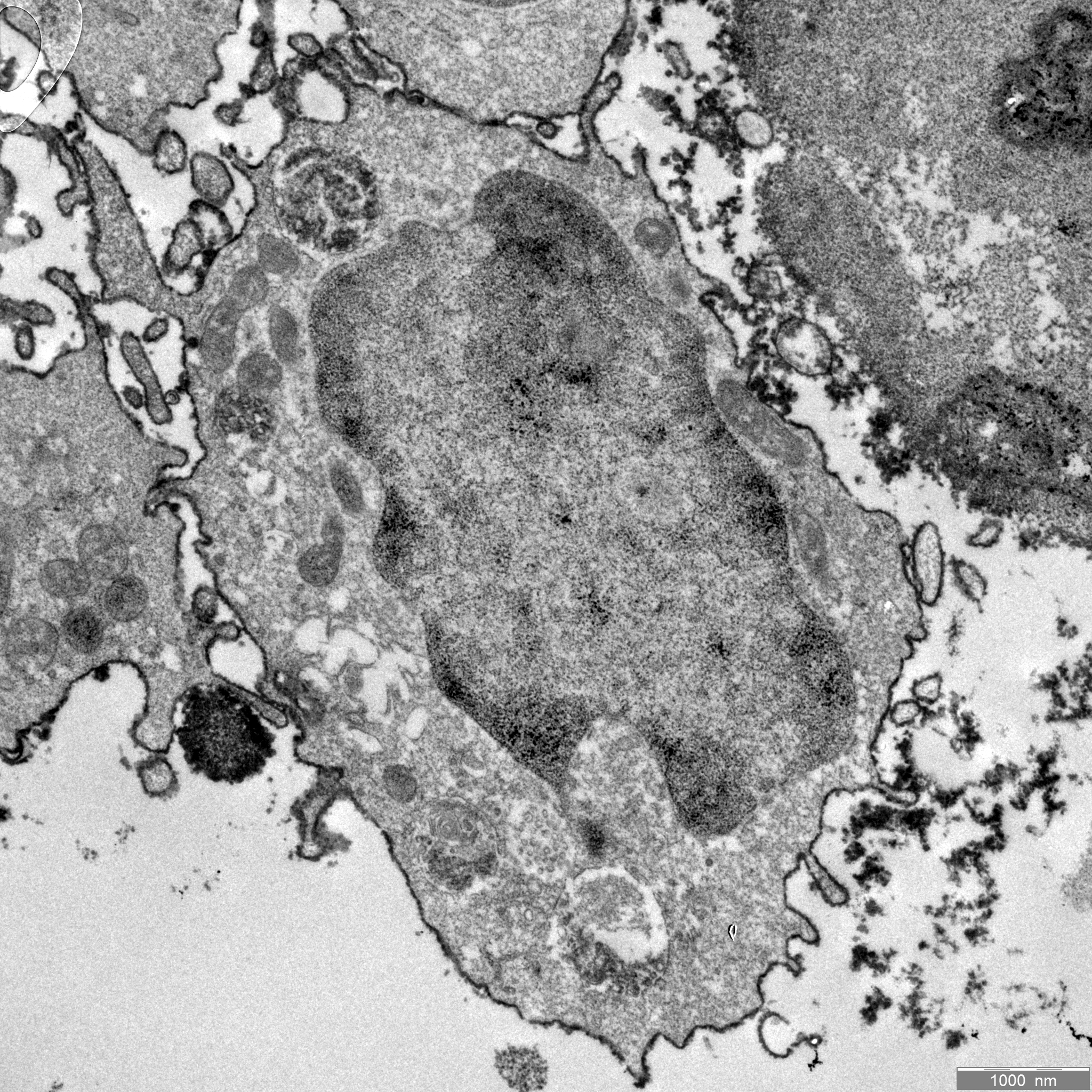

Supplement: Supplementary file 8 — Source data Fig. 5 [file 44319_2025_504_MOESM8_ESM.zip › Figure 5I/Wt_mLamp1_LPS+Bafilomycin_1000nm.TIF]

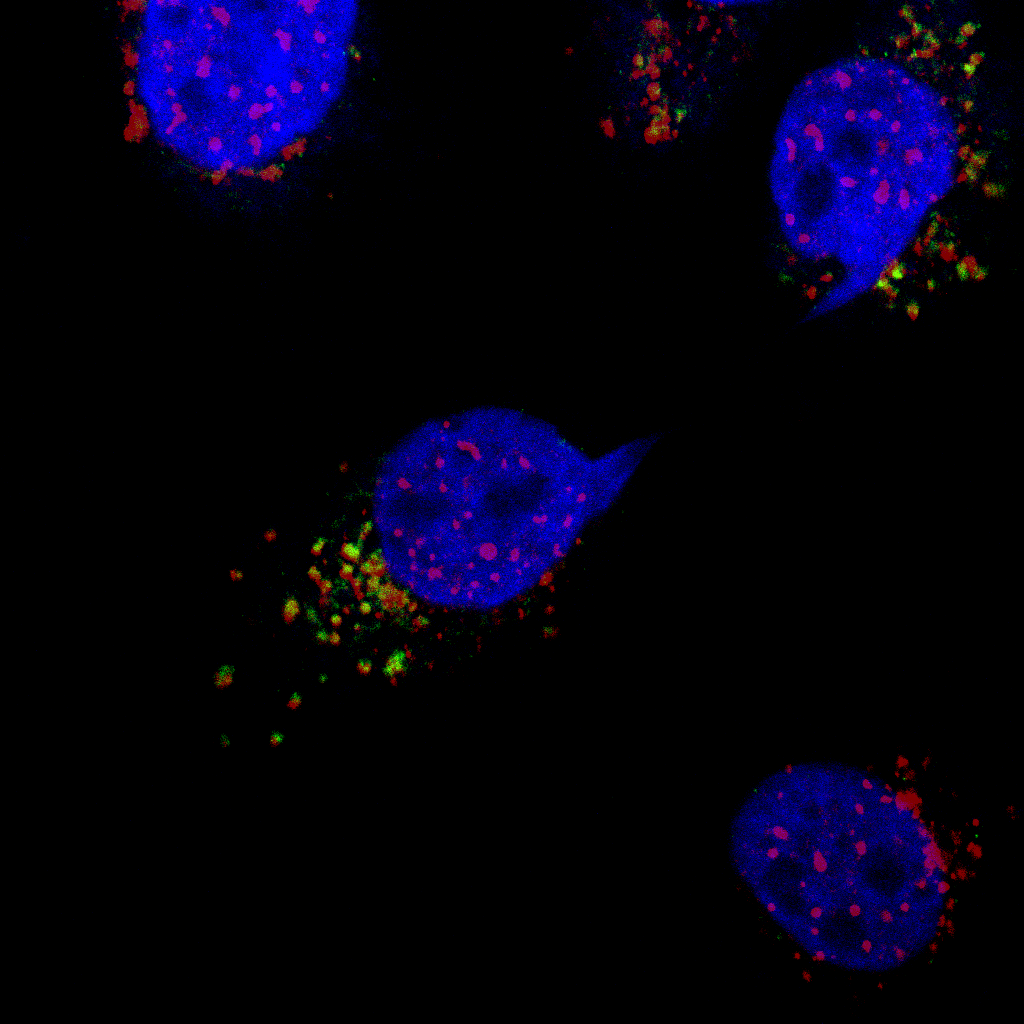

Supplement: Supplementary file 9 — Source data Fig. 6 [file 44319_2025_504_MOESM9_ESM.zip › Figure 6D/HaCat MP1-LC3 LRBA-KO DAPI-HLADR-LC3.tif]

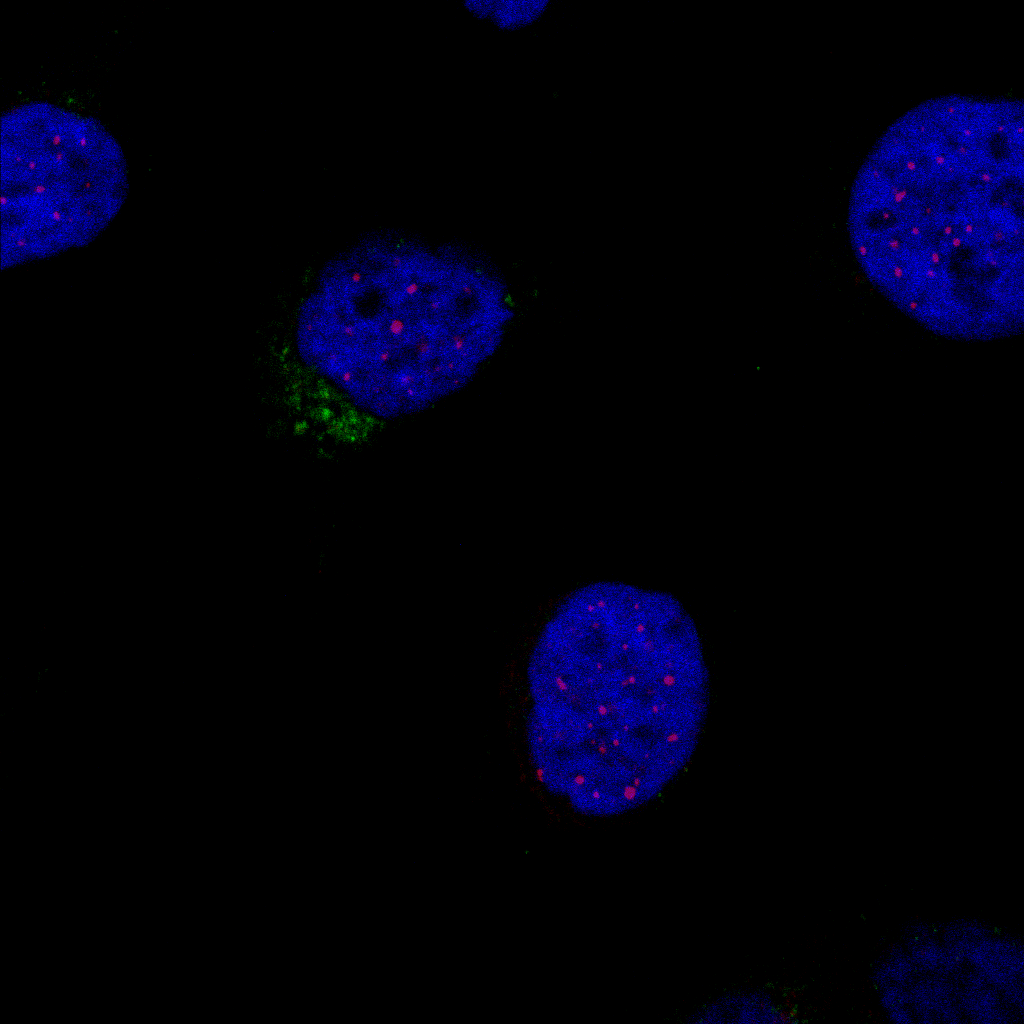

Supplement: Supplementary file 9 — Source data Fig. 6 [file 44319_2025_504_MOESM9_ESM.zip › Figure 6D/HaCat MP1-LC3 Myc-LRBA DAPI-HLADR-LC3.tif]

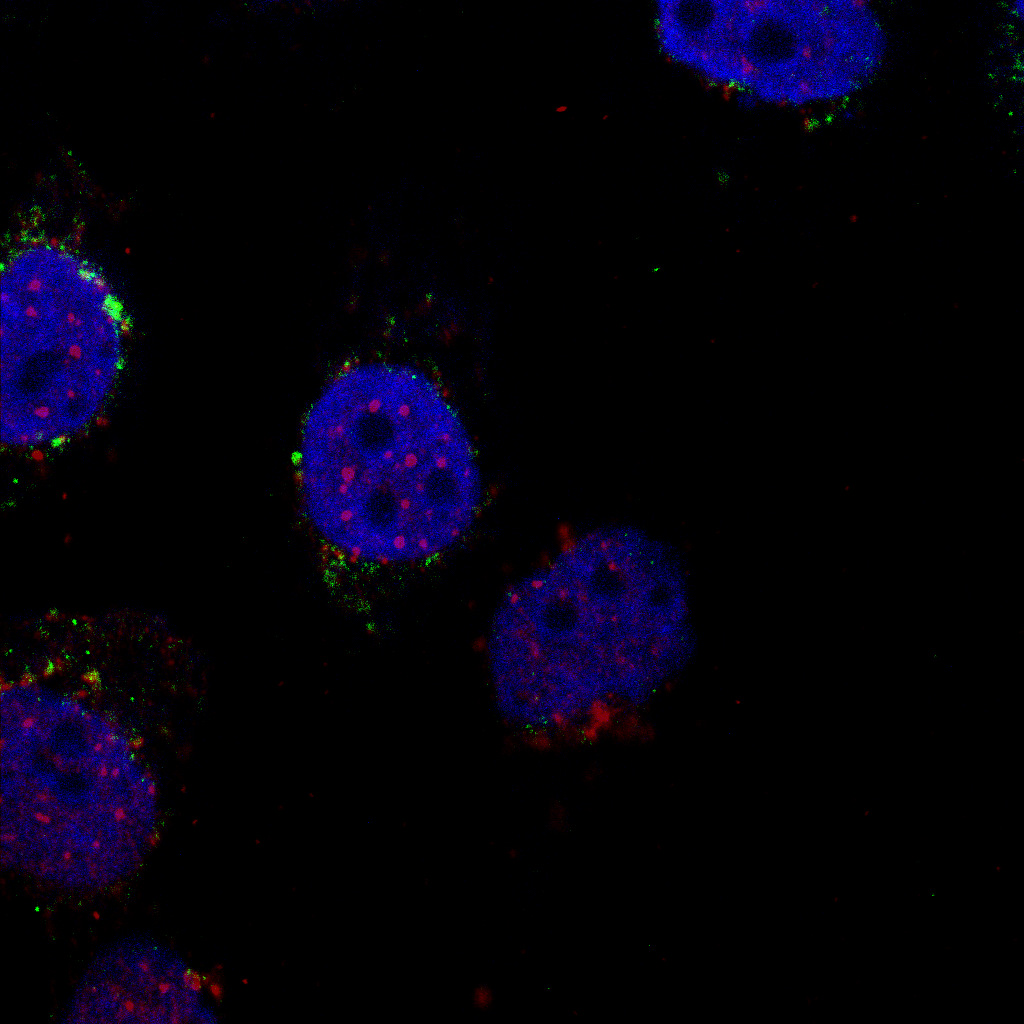

Supplement: Supplementary file 9 — Source data Fig. 6 [file 44319_2025_504_MOESM9_ESM.zip › Figure 6D/HaCat MP1-LC3 WT DAPI-HLADR-LC3.tif]
