# Supplementary material for: LRBA deficiency impairs autophagy and contributes to enhanced antigen presentation and T-cell dysregulation
Source: EMBO Rep. 2025 Jun 23;26(16):4040–71. doi: 10.1038/s44319-025-00504-7 (PMC12373796; doi:10.1038/s44319-025-00504-7)
Supplement: Supplementary file 10 — Expanded View Figures [file 44319_2025_504_MOESM10_ESM.pdf]

Expanded View Figures

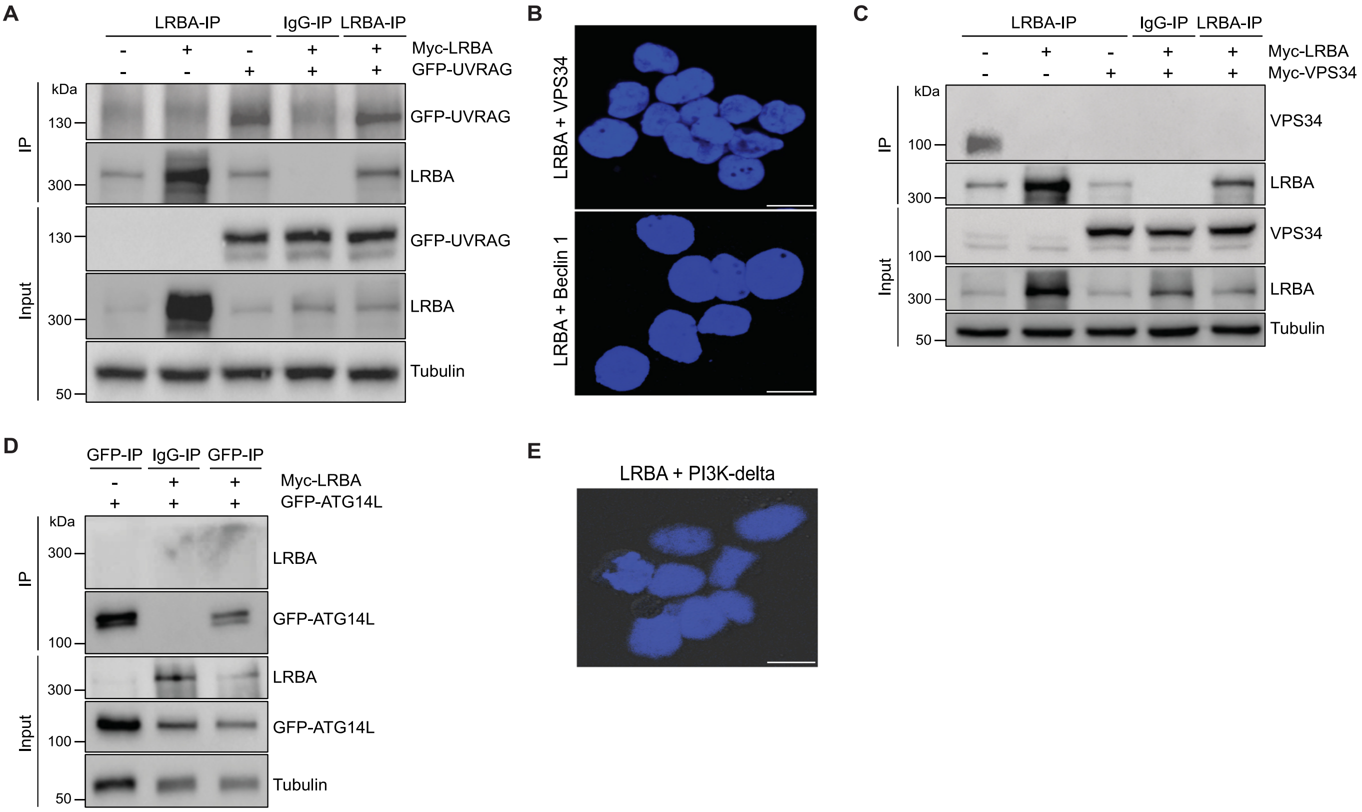

Figure EV1. LRBA interacts with UVRAG but not with other members of the PIKIII complex.

(**A**) Co-IP analysis revealed an interaction between LRBA and UVRAG in HEK293T WT cells transfected with Myc-LRBA and GFP-UVRAG plasmids. Immunoprecipitation was performed with anti-LRBA and immunoblotted for GFP. (**B**) PLA of LRBA and VPS34 (top) or Beclin-1 (bottom) in LCL cells from a healthy donor (HD) under resting conditions, revealing a lack of proximity. Nuclear DAPI staining is shown in blue. Scale bar=20 µm. (**C**) HEK293T WT cells were transfected with Myc-LRBA plasmid and/or Myc-VPS34/His-PIK3R4 plasmid (PIK3R4 blots are shown in Fig. 1E). Immunoprecipitation was performed with anti-LRBA and immunoblotted for VPS34. VPS34 and PIK3R4 were detected in the same experiment, therefore images of LRBA input and IP are similar as in Fig. 1E. (**D**) HEK293T WT cells were transfected with Myc-LRBA plasmid and GFP-ATG14L plasmid. Immunoprecipitation was performed with anti-LRBA and immunoblotted for GFP. (**E**) PLA showing the absence of proximity of LRBA with PI3Kdelta in LCL cells from a HD under resting conditions. Nuclear DAPI staining is shown in blue. Scale bar = 20 µm.

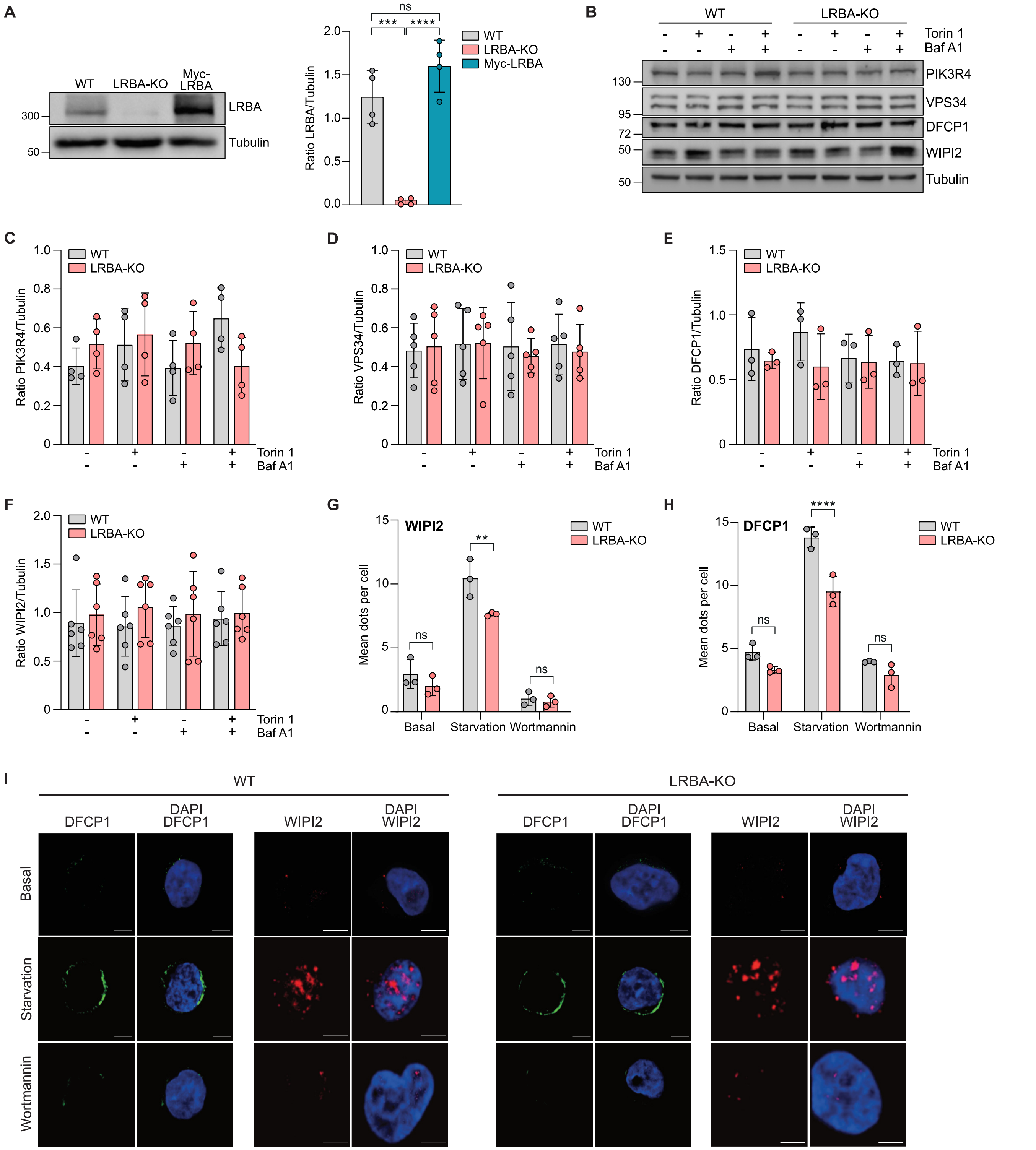
A
WT
LRBA-KO
Myc-LRBA
300
50
LRBA
Tubulin
ns
***
****
2.0
1.5
1.0
0.5
0.0
Ratio LRBA/Tubulin
WT
LRBA-KO
Myc-LRBA
B
WT
LRBA-KO
Torin 1
Baf A1
130
95
72
50
50
PIK3R4
VPS34
DFCP1
WIPI2
Tubulin
C
Ratio PIK3R4/Tubulin
WT
LRBA-KO
Torin 1
Baf A1
D
Ratio VPS34/Tubulin
WT
LRBA-KO
Torin 1
Baf A1
E
Ratio DFCP1/Tubulin
WT
LRBA-KO
Torin 1
Baf A1
F
Ratio WIPI2/Tubulin
WT
LRBA-KO
Torin 1
Baf A1
G
WIPI2
Mean dots per cell
WT
LRBA-KO
ns
**
ns
Basal
Starvation
Wortmannin
H
DFCP1
Mean dots per cell
WT
LRBA-KO
ns
****
ns
Basal
Starvation
Wortmannin
I
WT
LRBA-KO
DFCP1
DAPI DFCP1
WIPI2
DAPI WIPI2
Basal
Starvation
Wortmannin

 © The Author(s)

Figure EV2. Diminished DFCP1/WIPI2 punctae under starvation.

(**A**) Representative immunoblot (left) and densitometry analyses (right) of LRBA expression in relation to Tubulin in WT (grey), LRBA-KO (red) and Myc-LRBA reconstituted (teal) HEK293T cells. Each dot represent one blot while bars represent the mean ± SD of $n = 4$ independent biological replicates. (**B–F**) Loss of LRBA does not affect the protein expression of PIK3R4, VPS34, DFCP-1 and WIPI-2. Representative immunoblot (**B**) and densitometry analysis of the protein levels of (**C**) PIK3R4, (**D**) VPS34, (**E**) DFCP1 and (**F**) WIPI2 in relation to Tubulin in the presence and absence of Torin 1, with or without Bafilomycin A1, for WT and LRBA-KO HEK293T cells. Each dot represents the densitometry analysis of one blot while bars represent the mean ± SD from $n = 3$–6 independent biological replicates. (**G–I**) (**G**) Quantification of WIPI2 and (**H**) DFCP1 expression in WT (grey) and LRBA-KO (red) HEK293T cells cultured for 1 h with EBSS or 100 nM Wortmannin. Each dot represents the mean of one experiment while bars represent the mean ± SD of $n = 3$ independent biological replicates. Total cells analyzed (WIPI2/DFCP1) for WT EBSS: 14/60 cells and KO = 28/42 cells. For WT=Wortmannin 29/57 cells and KO = 26/83 cells. (**I**) Representative confocal microscopy images of DFCP1 (green) and WIPI2 (red) signal upon staining with anti-DFCP1 and anti-WIPI2 antibodies. Scale bar=5 µm. Statistical analyses of (**A**) was performed using a one-way ANOVA with Tukey's multiple comparisons test and for (**C–H**) a two-way ANOVA with Bonferroni's multiple comparisons test, $^{**}P < 0.01$ ((**G**): $P = 0.0052$), $^{***}P < 0.001$ ((**A**): $P = 0.0006$), $^{****}P < 0.0001$.

© The Author(s)

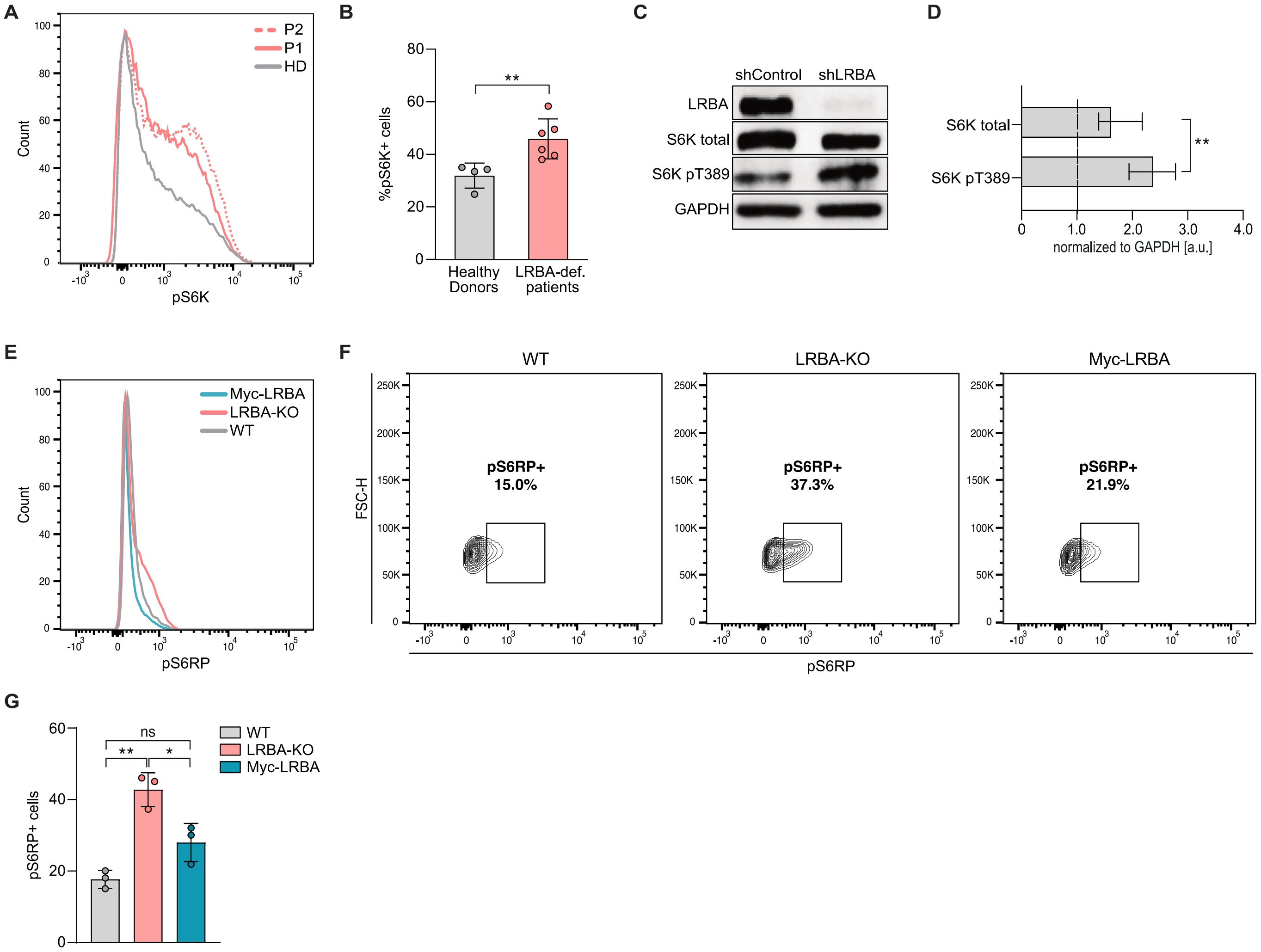

Figure EV3. Enhanced mTOR signaling in LRBA-deficient cells.

(**A**) Representative histogram of pS6K-MFI detected by flow cytometry in LCL from HD (grey) and LRBA-deficient patients (red) at basal levels. (**B**) Bar graphs representing the percentage of cells positive for pS6K in HD (grey) and two LRBA-deficient patients (red). Each dot represents the mean of $n = 2$ technical replicates while bars present the mean ± SD of $n = 4$ independent biological replicates. (**C**) Representative immunoblot analyses of LRBA, S6K total, S6K pT389 and GAPDH performed in shControl and shLRBA-HeLa cells at basal conditions. (**D**) Bar graphs represent densitometry analyses of S6K total and S6K pT389 expression from HeLa cells. Quantifications of protein expression were previously normalized to GAPDH and to shControl (dotted line). The bars represent the means ± SD from $n = 4$ independent biological replicates. (**E–G**) pS6RP levels were rescued after LRBA reconstitution. (**E**) Representative histogram of pS6RP-MFI (**F**) representative dot plot of p6RP+ cells, and (**G**) percentage of cells positive for pS6RP that was detected by flow cytometry in WT (grey), LRBA-KO (red) and Myc-LRBA (teal) at basal levels. Each dot represents the mean of $n = 2$ technical replicates while bars present the mean ± SD of $n = 3$ independent biological replicates. Statistical analyses of (**B**, **D**) was performed using a unpaired Welch's t test and for (**G**) a one-way ANOVA with Tukey's multiple comparisons test, $^{*}P < 0.05$ ((**G**): $P = 0.0144$), $^{**}P < 0.01$ ((**B**): $P = 0.0073$), ((**G**): $P = 0.001$).

 © The Author(s)

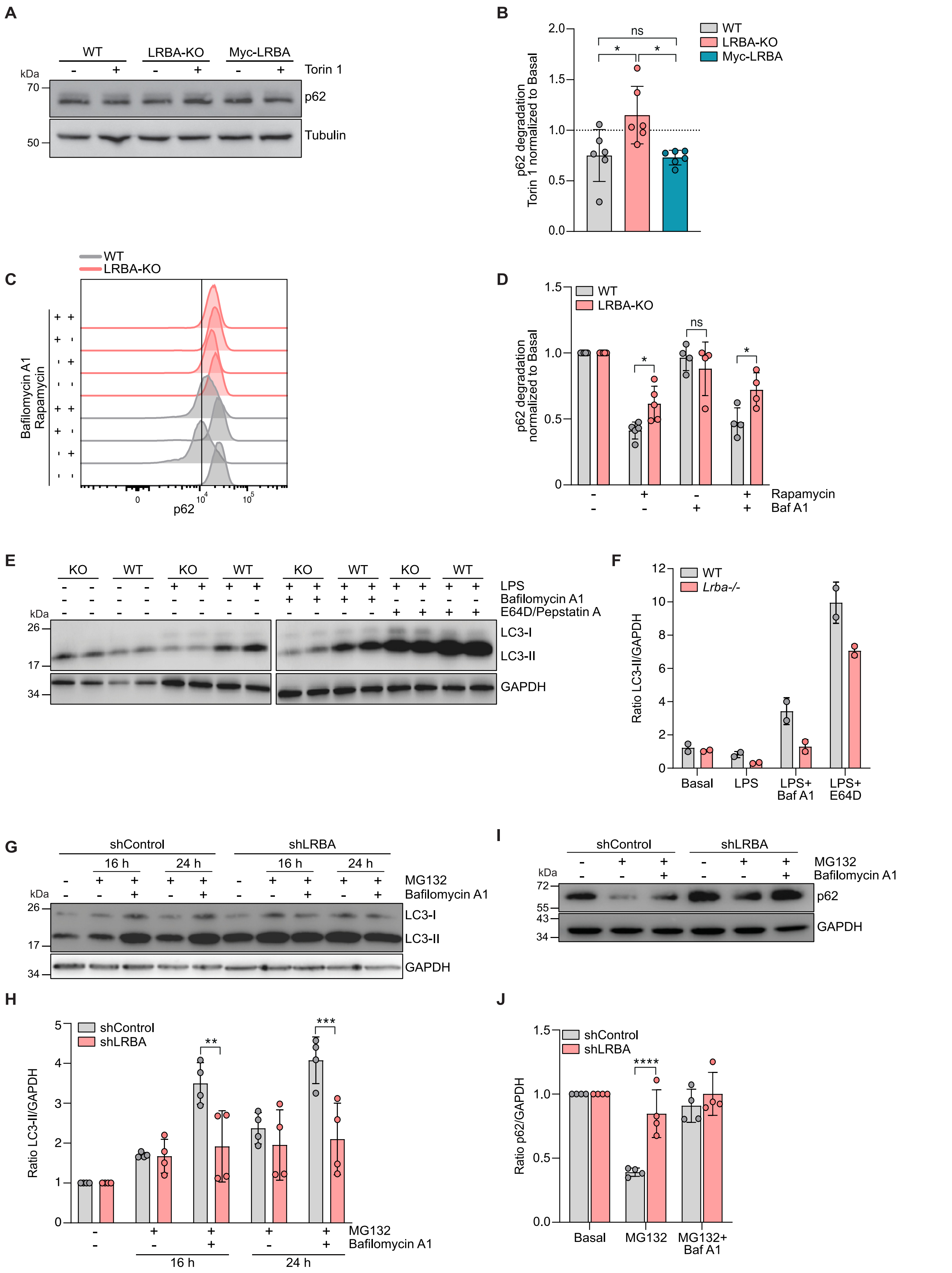
A
WT
LRBA-KO
Myc-LRBA
Torin 1
kDa
70
50
p62
Tubulin
B
p62 degradation Torin 1 normalized to Basal
WT
LRBA-KO
Myc-LRBA
ns
*
*
C
WT
LRBA-KO
Bafilomycin A1
Rapamycin
p62
D
p62 degradation normalized to Basal
WT
LRBA-KO
ns
*
*
Rapamycin
Baf A1
E
KO
WT
LPS
Bafilomycin A1
E64D/Pepstatin A
kDa
26
17
34
LC3-I
LC3-II
GAPDH
F
Ratio LC3-II/GAPDH
WT
Lrba-/-
Basal
LPS
LPS+ Baf A1
LPS+ E64D
G
shControl
shLRBA
16 h
24 h
MG132
Bafilomycin A1
kDa
26
17
34
LC3-I
LC3-II
GAPDH
H
Ratio LC3-II/GAPDH
shControl
shLRBA
**
***
MG132
Bafilomycin A1
16 h
24 h
I
shControl
shLRBA
MG132
Bafilomycin A1
kDa
72
55
43
34
p62
GAPDH
J
Ratio p62/GAPDH
shControl
shLRBA
****
Basal
MG132
MG132+ Baf A1

Figure EV4. Abnormal autophagy flux in shLRBA HeLa cells and B cells from *Lrba−/−* mice.

(**A**) Representative immunoblot analyses of p62 degradation in WT, LRBA-KO and Myc-LRBA HEK293T cells at basal conditions or after Torin 1 stimulation. (**B**) Densitometry analysis of (**A**) of WT (grey), LRBA-KO (red) and Myc-LRBA (teal) HEK293T cells. Each dot represents the densitometry analysis of one blot while bars represent the mean ± SD from $n = 6$ independent biological replicates. (**C**, **D**) Representative histograms of p62 degradation in WT (grey) and LRBA-KO (red) Ramos B cells in the presence and absence of Rapamycin, with or without Bafilomycin A1 and (**D**) fold change of p62 degradation normalized to basal levels. Each dot represents the fold change of one technical replicate while bars represent the mean ± SD from $n = 4$–5 independent biological replicates. (**E**, **F**) Reduced autophagy flux in splenic naive B cells from *Lrba*$^{-/-}$ mice. (**E**) Representative immunoblot analyses of LC3-I and LC3-II processing and Tubulin in isolated splenocytes from WT and Lrba$^{-/-}$ mice at day 0 or upon stimulation for 3 days with 20 µg/ml LPS alone or in the presence of 100 nM Bafilomycin A1 or protease inhibitors E64D and Pepstatin. (**F**) Densitometry analyses of LC3-II expression relative to Tubulin of splenic murine naive B cells from WT (grey) and *Lrba−/−* (red) mice. Each dot represents the densitometry analysis of one blot while bars represent the mean ± SD from $n = 2$ independent biological replicates. (**G**) Representative immunoblot analyses of the processing of endogenous unconjugated LC3-I to lipid-conjugated LC3-II in shControl and shLRBA HeLa cells at resting conditions or after 16 h or 24 h of MG132 alone or in the presence of 100 nM Bafilomycin A1. (**H**) Densitometry analyses of LC3-II expression relative to GAPDH of shControl (grey) and shLRBA (red) HeLa cells. Each dot represents the densitometry analysis of one blot while bars represent the mean ± SD from $n = 4$ independent biological replicates. (**I**) Representative immunoblot analyses of p62 and GAPDH in shControl and shLRBA HeLa cells at resting conditions or after 16 h treatment with MG132 alone or along with 100 nM Bafilomycin A1. (**J**) Densitometry analyses of p62 relative to GAPDH and normalized to basal conditions of shControl (grey) and shLRBA (red) HeLa cells. Each dot represents the densitometry analysis of one blot while bars represent the mean ± SD from $n = 4$ independent biological replicates. Statistical analysis for (**B**) was performed using a one-way ANOVA with Tukey's multiple comparisons test and for (**D**, **H**, **I**) a two-way ANOVA with Bonferroni's multiple comparisons test, *$P < 0.05$ (**B**: $P = 0.02$ WT vs KO and $P = 0.0146$ KO vs Myc-LRBA), (**D**: $P = 0.0237$ Rapamycin and $P = 0.0132$ Rapamycin+Bafilomycin A1), **$P < 0.01$ ((**H**): $P = 0.0027$), ***$P < 0.001$ ((**H**): $P = 0.0002$), ****$P < 0.0001$.

© The Author(s)

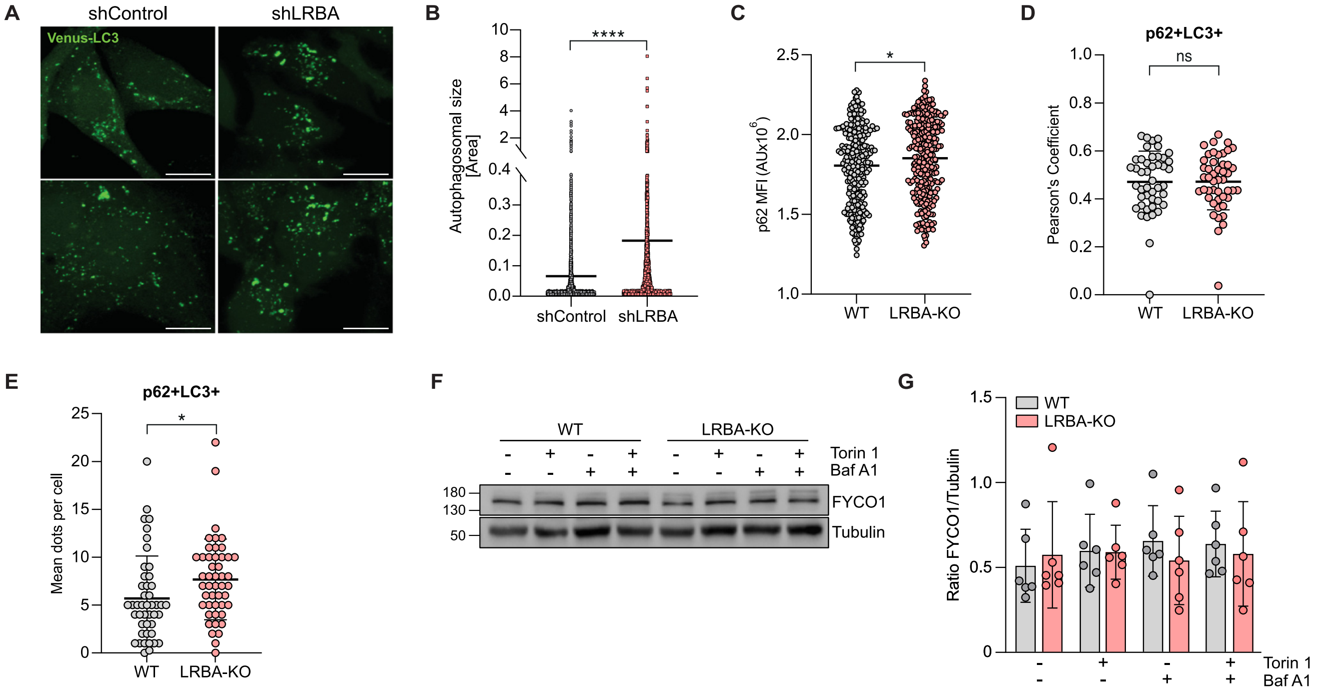

Figure EV5. Loss of LRBA leads to enlarged autophagosomes.

(**A**, **B**) shControl and shLRBA HeLa cells were transfected with GFP-LC3 plasmid and incubated for 2 h with 100 nM of Bafilomycin A1 for microscopy evaluation. (**A**) Representative confocal microscopy images of LC3-transfected shControl and shLRBA HeLa cells (green). Scale bar = 10 µm. (**B**) Size of GFP-LC3 punctae in shControl (grey) and shLRBA (red) HeLa cells was determined from binary images using the analyses particle module of FIJI, with a particle size from 0 to 20 µm^2. Each dot represents an autophagosome from $n = 3$ fields across $n = 3$ independent biological replicates. Total autophagosomes for shControl = 961 and shLRBA= 1249. (**C–E**) Scatter plots showing the (**C**) p62 MFI in p62+LC3+ dots (**D**) co-localization of LC3 and p62 and (**E**) number of LC3+p62+ dots in WT (grey) and LRBA-KO (red) HaCat cells. Each dot for (**C**) represents one autophagosome from $n = 3$ independent biological replicates with a total of $n = 262$ (WT) and $n = 225$ (LRBA-KO) analyzed and for (**D**, **E**) one cell from $n = 3$ independent biological replicates with a total of at least 43 cells analyzed per condition, mean ± SD shown by the black line and error bars. (**F**) Representative immunoblot analyses and (**G**) densitometry analysis of FYCO1 and Tubulin expression upon basal conditions and stimulation with Torin 1 in presence or absence of Bafilomycin A1. Each dot represents the densitometry analysis of one blot while bars represent the mean ± SD from $n = 6$ independent biological replicates. Statistical analysis for (**B–E**) was performed using an unpaired Welch's t test and for (**G**) two-way ANOVA with Bonferroni's multiple comparisons test, $*P < 0.05$ ((**C**): $P = 0.0258$), ((**E**): $P = 0.0304$), $****P < 0.0001$.

© The Author(s)

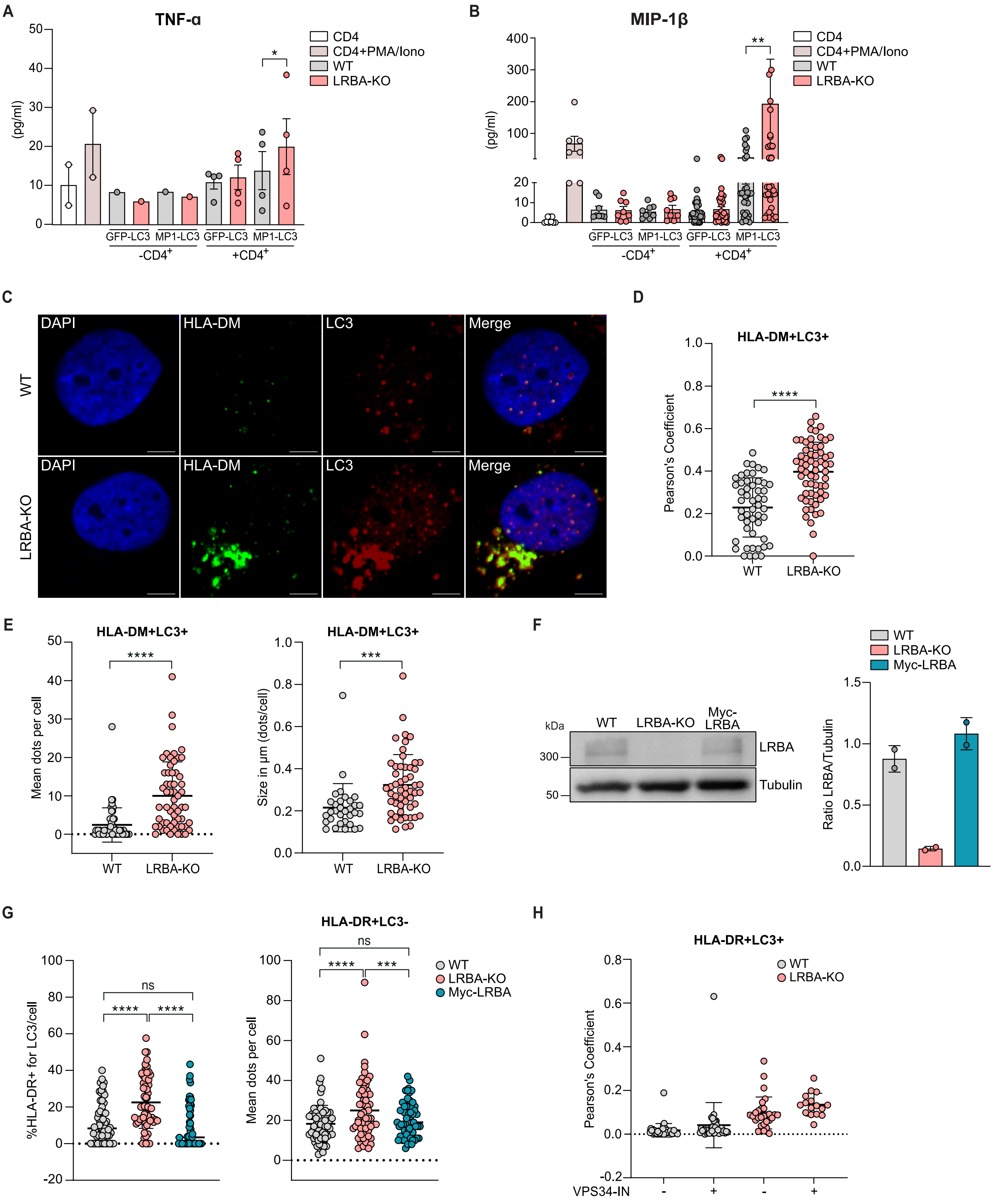
A
TNF-α
(pg/ml)
CD4
CD4+PMA/Iono
WT
LRBA-KO
GFP-LC3 MP1-LC3 GFP-LC3 MP1-LC3
-CD4+ +CD4+
B
MIP-1β
(pg/ml)
CD4
CD4+PMA/Iono
WT
LRBA-KO
GFP-LC3 MP1-LC3 GFP-LC3 MP1-LC3
-CD4+ +CD4+
C
DAPI
HLA-DM
LC3
Merge
WT
LRBA-KO
D
HLA-DM+LC3+
Pearson's Coefficient
WT LRBA-KO
E
HLA-DM+LC3+
Mean dots per cell
WT LRBA-KO
HLA-DM+LC3+
Size in µm (dots/cell)
WT LRBA-KO
F
kDa
WT LRBA-KO Myc-LRBA
LRBA
Tubulin
WT
LRBA-KO
Myc-LRBA
Ratio LRBA/Tubulin
G
%HLA-DR+ for LC3/cell
ns
HLA-DR+LC3-
Mean dots per cell
WT
LRBA-KO
Myc-LRBA
H
HLA-DR+LC3+
Pearson's Coefficient
WT
LRBA-KO
VPS34-IN

 © The Author(s)

Figure EV6. Loss of LRBA leads to increased TNF-α and MP-1β release *via* autophagy.

(**A**, **B**) WT and LRBA-KO HaCat cells stably expressing GFP-LC3 or MP1-LC3 (target cells), and pre-treated with IFN-γ for 24 h to up-regulate MHC-II were cultured with a MP1-specific $CD4^+$ T cell clone (effector cells) from a HD for 20 h. Following incubation, TNF-α and MIP-1β were measured in the culture supernatants by ELISA. Each dot represents one technical replicate while bars represent the mean ± SD of (**A**) TNF-α ($n = 4$ independent biological replicates for HaCat GFP/MP-1-LC3 cells in presence of CD4+ T cells, whereas $n = 1$ for HaCat GFP/MP-1-LC3 without CD4+ T cells, and $n = 2$ for CD4+ cells alone) and (**B**) MIP-1β ($n = 4$ independent biological replicates) secretion in WT (grey) and LRBA-KO (red) HaCat cells (**C**) Representative confocal microscopy images of WT and LRBA-KO HaCat cells stimulated overnight with IFN-γ and treated with 20 μM chloroquine for 6 h. Fixed cells were stained for HLA-DM (green), LC3 (red) and DAPI (blue) for the nuclei. Scale bar = 5 μm. (**D**, **E**) Scatter plots showing the (**D**) co-localization of LC3 and HLA-DM and (**E**) the number of dots per cell (left) and size (right) of vesicles positive for HLA-DM and LC3 in WT (grey) and LRBA-KO (red) HaCat cells. Each dot represents one cell from $n = 3$ independent biological replicates with a total of at least 35 cells analyzed per condition, mean ± SD shown by the black line and error bars. (**F**) Representative immunoblot and densitometry analyses of LRBA expression in WT (grey), LRBA-KO (red) and Myc-LRBA (teal) reconstituted HaCat cells. Each dot represents the densitometry analysis of one blot while bars represent the mean ± SD from $n = 2$ independent biological replicates (**G**) Scatter plots showing co-localization of HLA-DR and LC3 (left) and number of HLA-DR+LC3- dots (right) in WT (grey), LRBA-KO (red) and Myc-LRBA (teal) reconstituted HaCat cells. Each dot represents one cell from $n = 3$ independent biological replicates with a total of at least 30 cells analyzed per condition, mean ± SD shown by the black line and error bars. (**H**) Scatter plots showing the co-localization of HLA-DR and LC3 in unstimulated WT (grey) and KO (red) HaCat cells with or without VPS34 inhibitor (0.1 μM) treatment. Each dot represents one cell from $n = 2$ biological replicates with a total of at least 30 cells analyzed per condition, mean ± SD shown by the black line and error bars. Statistical analyses of (**A**, **B**) was performed using a ratio paired Student *t* test, for (**D**, **E**) a unpaired Welch's *t* test, for (**G**) a one-way ANOVA with Tukey's multiple comparisons test and for (**H**) and a two-way ANOVA with Bonferroni's multiple comparisons, *$P < 0.05$ ((**A**): $P = 0.0410$), **$P < 0.01$ ((**B**): $P = 0.0014$), ***$P < 0.0001$ ((**E**): $P = 0.0003$), ((**G**): $P = 0.0004$), ****$P < 0.0001$.

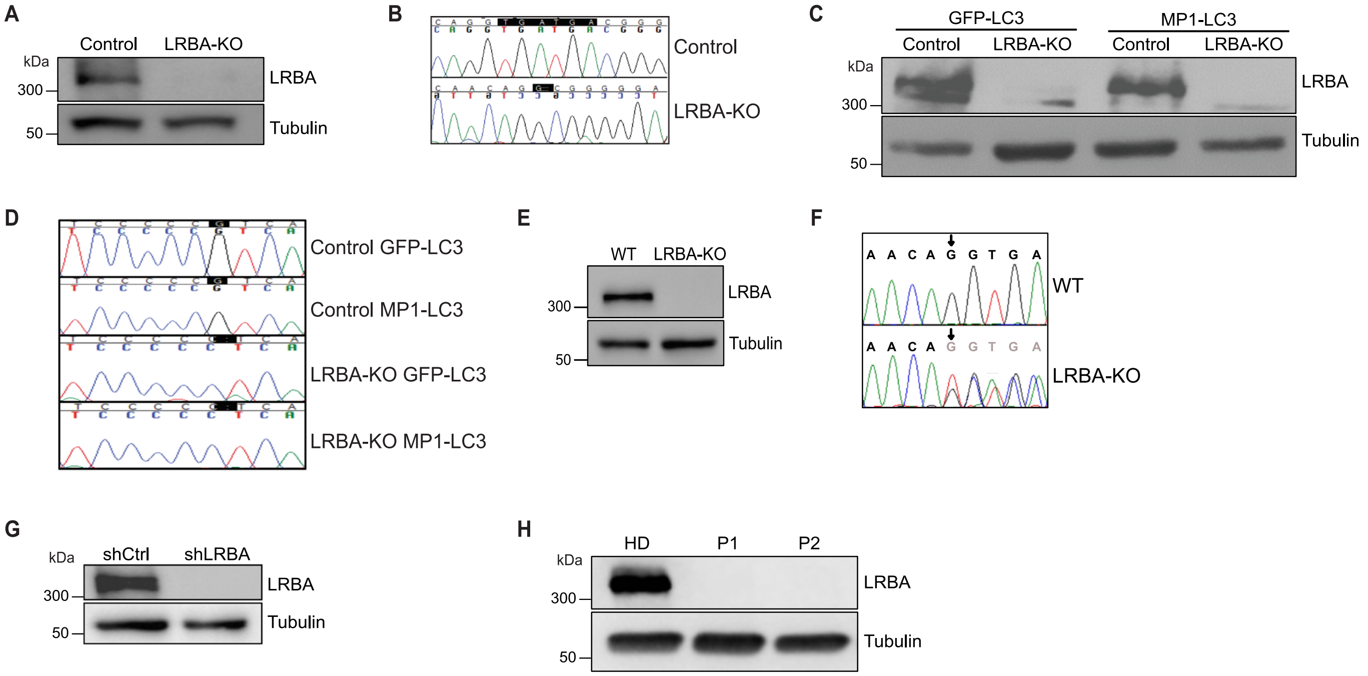

Figure EV7. Generation of LRBA-deficient cell lines.

(**A**, **C**, **E**) Immunoblot analyses of LRBA protein expression in WT and LRBA-KO (**A**) HEK293T, (**C**) HaCaT and (**E**) Ramos cells. (**B**, **D**, **F**) Sequencing analyses of *LRBA* exon 2 cells showing successful depletion of LRBA using the CRISPR-Cas9 system in (**B**) HEK293T, (**D**) HaCaT and (**F**) Ramos cells. (**G**) Immunoblot analyses of LRBA protein expression in HeLa cells shcontrol and shLRBA HeLa cells. (**H**) Immunoblot analyses of LRBA protein expression in LCL cells from a HD and two LRBA-deficient patients.

 © The Author(s)
